# Disrupting circadian control of peripheral myogenic reactivity mitigates cardiac injury following myocardial infarction

**DOI:** 10.1093/cvr/cvac174

**Published:** 2022-11-22

**Authors:** Jeffrey T Kroetsch, Darcy Lidington, Faisal J Alibhai, Cristine J Reitz, Hangjun Zhang, Danny D Dinh, Julia Hanchard, Tarak N Khatua, Scott P Heximer, Tami A Martino, Steffen-Sebastian Bolz

**Affiliations:** Department of Physiology, University of Toronto, Toronto, Canada; Toronto Centre for Microvascular Medicine at The Ted Rogers Centre for Heart Research Translational Biology and Engineering Program, University of Toronto, 661 University Avenue, 14th Floor, Toronto, Ontario M5G 1M1, Canada; Department of Physiology, University of Toronto, Toronto, Canada; Toronto Centre for Microvascular Medicine at The Ted Rogers Centre for Heart Research Translational Biology and Engineering Program, University of Toronto, 661 University Avenue, 14th Floor, Toronto, Ontario M5G 1M1, Canada; Centre for Cardiovascular Investigations, Department of Biomedical Sciences, University of Guelph, Guelph, Canada; Centre for Cardiovascular Investigations, Department of Biomedical Sciences, University of Guelph, Guelph, Canada; Department of Physiology, University of Toronto, Toronto, Canada; Department of Physiology, University of Toronto, Toronto, Canada; Toronto Centre for Microvascular Medicine at The Ted Rogers Centre for Heart Research Translational Biology and Engineering Program, University of Toronto, 661 University Avenue, 14th Floor, Toronto, Ontario M5G 1M1, Canada; Department of Physiology, University of Toronto, Toronto, Canada; Toronto Centre for Microvascular Medicine at The Ted Rogers Centre for Heart Research Translational Biology and Engineering Program, University of Toronto, 661 University Avenue, 14th Floor, Toronto, Ontario M5G 1M1, Canada; Centre for Cardiovascular Investigations, Department of Biomedical Sciences, University of Guelph, Guelph, Canada; Department of Physiology, University of Toronto, Toronto, Canada; Heart & Stroke/Richard Lewar Centre of Excellence for Cardiovascular Research, University of Toronto, Toronto, Canada; Centre for Cardiovascular Investigations, Department of Biomedical Sciences, University of Guelph, Guelph, Canada; Department of Physiology, University of Toronto, Toronto, Canada; Toronto Centre for Microvascular Medicine at The Ted Rogers Centre for Heart Research Translational Biology and Engineering Program, University of Toronto, 661 University Avenue, 14th Floor, Toronto, Ontario M5G 1M1, Canada; Heart & Stroke/Richard Lewar Centre of Excellence for Cardiovascular Research, University of Toronto, Toronto, Canada

**Keywords:** Vascular smooth muscle cells, Systemic hemodynamics, Total peripheral resistance, Casein kinase 1, Tumour necrosis factor

## Abstract

**Aims:**

Circadian rhythms orchestrate important functions in the cardiovascular system: the contribution of microvascular rhythms to cardiovascular disease progression/severity is unknown. This study hypothesized that (i) myogenic reactivity in skeletal muscle resistance arteries is rhythmic and (ii) disrupting this rhythmicity would alter cardiac injury post-myocardial infarction (MI).

**Methods and results:**

Cremaster skeletal muscle resistance arteries were isolated and assessed using standard pressure myography. Circadian rhythmicity was globally disrupted with the Clock^Δ19/Δ19^ mutation or discretely through smooth muscle cell-specific Bmal1 deletion (Sm-Bmal1 KO). Cardiac structure and function were determined by echocardiographic, hemodynamic and histological assessments. Myogenic reactivity in cremaster muscle resistance arteries is rhythmic. This rhythm is putatively mediated by the circadian modulation of a mechanosensitive signalosome incorporating tumour necrosis factor and casein kinase 1. Following left anterior descending coronary artery ligation, myogenic responsiveness is locked at the circadian maximum, although circadian molecular clock gene expression cycles normally. Disrupting the molecular clock abolishes myogenic rhythmicity: myogenic tone is suspended at the circadian minimum and is no longer augmented by MI. The reduced myogenic tone in Clock^Δ19/Δ19^ mice and Sm-Bmal1 KO mice associates with reduced total peripheral resistance (TPR), improved cardiac function and reduced infarct expansion post-MI.

**Conclusions:**

Augmented microvascular constriction aggravates cardiac injury post-MI. Following MI, skeletal muscle resistance artery myogenic reactivity increases specifically within the rest phase, when TPR would normally decline. Disrupting the circadian clock interrupts the MI-induced augmentation in myogenic reactivity: therapeutics targeting the molecular clock, therefore, may be useful for improving MI outcomes.

## Graphical Abstract

**Graphical Abstract:**
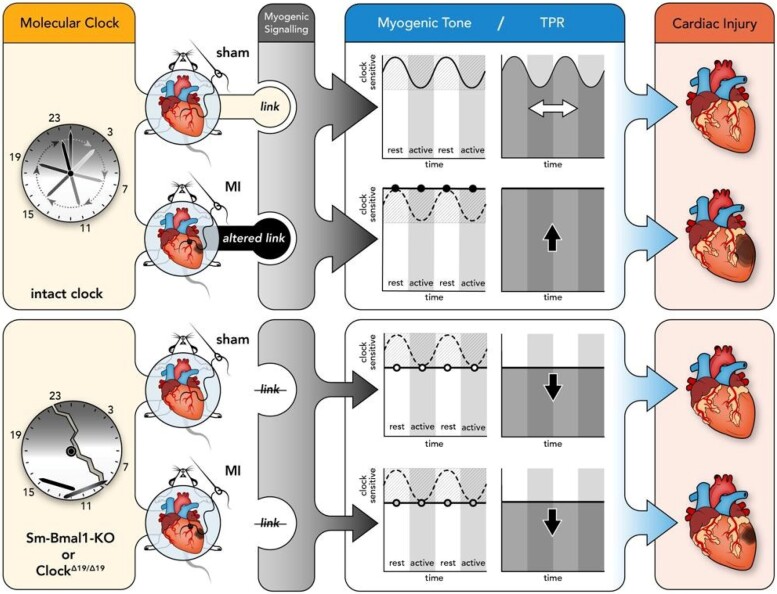

## 1. Introduction

Following myocardial infarction (MI), total peripheral resistance (TPR) increases to counteract the reduction in cardiac output. While this TPR elevation is classically described as a compensatory response that maintains mean arterial pressure (MAP),^1,2^ the resulting increase in cardiac afterload strains the injured heart and promotes both infarct expansion and deleterious ventricular remodelling.^3,4^ These processes ultimately drive a progressive decline in cardiac function and patient outcome. Consequently, current clinical interventions endeavour to reduce afterload immediately following MI.

Resistance arteries are key determinants of TPR and represent ‘functional hotspots’ in disease processes that alter systemic hemodynamics, including MI, heart failure, diabetes, and hypertension. Since the skeletal muscle vascular bed forms the body’s largest circulatory network (40% of body mass is skeletal muscle), skeletal muscle resistance arteries prominently contribute to the regulation of TPR.^5,6^ Consequently, the molecular mechanisms that regulate skeletal muscle resistance artery tone are key targets for interventions seeking to reduce TPR. In this regard, the myogenic response, an intrinsic mechanism that matches microvascular resistance to blood pressure, governs up to 75% of resistance artery tone.^7^ Yet, despite its prominent role in regulating TPR and significant therapeutic potential, the myogenic response remains an underappreciated and under-researched therapeutic target for reducing microvascular resistance. Understanding the molecular mechanisms that control myogenic reactivity could, therefore, unlock new opportunities to therapeutically control TPR and blood pressure.

Many cellular processes display circadian rhythmicity (i.e. they are entrained via recurring daily environmental cues), which allows organisms to anticipate environmental changes and optimize their physiological functions.^8^ In the cardiovascular system, the influence of circadian rhythms is illustrated by the *timed* interplay between the heart and the peripheral microvasculature, where significant anti-phasic oscillations of cardiac output and TPR yield only modest daily fluctuations in MAP. While there is clear evidence that TPR and hence, microvascular contractility are under circadian control,^9–11^ few investigations have explored whether the underlying mechanisms can be harnessed to reduce TPR or cardiac injury.

The present study hypothesized that: (i) myogenic reactivity in skeletal muscle resistance arteries is rhythmic and (ii) disrupting this circadian rhythm would alter cardiac injury post-MI. Our investigation supports both of these hypotheses. Specifically, a proportion of cremaster skeletal muscle resistance artery myogenic reactivity is under circadian control. Disrupting the key signalling effectors of membrane-bound tumour necrosis factor (mTNF) reverse signalling^5^ abolishes the circadian oscillations in myogenic tone, consistent with the conclusion that the smooth muscle cell peripheral clock governs myogenic mechanotransduction. Our previous work demonstrates that MI augments skeletal muscle resistance artery myogenic reactivity:^5,12^ the present study reveals that this augmentation relies on an uncoupling of myogenic reactivity from circadian control. Finally, disrupting the molecular clock, either globally or within vascular smooth muscle cells, suspends myogenic reactivity at its circadian minimum and prevents its augmentation following an MI. The attenuated myogenic reactivity associates with reduced TPR, reduced infarct expansion and less pathological ventricular remodelling.

## 2. Methods

### 2.1 Ethics approval

This investigation conforms to the National Research Council’s 2011 *Guide for the Care and Use of Laboratory Animals* (ISBN: 0-309-15400-6). All experimental protocols were approved by the Institutional Animal Care and Use Committee at the University of Toronto and at the University of Guelph, and conducted according to Canadian animal protection laws.

### 2.2 Animals

This study utilized 2–3 month old male mice. Wild-type (WT) C57BL/6N mice were purchased from Charles River Laboratories (Saint-Constant, Canada). Germline *tumour necrosis factor* knockout mice (TNF KO) were purchased from Taconic Biosciences (Hudson, USA).^13^ Tamoxifen inducible, smooth muscle cell-targeted Bmal1 knockout mice (Sm-Bmal1 KO) were generated by crossing floxed *Bmal1* mice^14^ with mice expressing a recombinant Cre recombinase under the control of a smooth muscle promoter (SMMHC-CreER^T2^).^15^ Deletion of the floxed *Bmal1* gene in mice was achieved with 3 days of tamoxifen treatment (1 mg/day i.p. dissolved in corn oil). Tamoxifen-treated littermates expressing CreER^T2^ and non-floxed *Bmal1* served as WT controls (Cre-WT). Mice homozygous for the CLOCK delta 19 point mutation (Clock^Δ19/Δ19^)^16^ were bred on a C57BL/6J background. Clock^Δ19/Δ19^ mice were maintained in the Central Animal Facilities at the University of Guelph;^17^ all other animals were maintained at the University of Toronto Department of Comparative Medicine animal facility. All animals were housed under 12:12 light-dark cycles, with *ad libitum* access to water and chow. Upon reaching experimental endpoints, animals were deeply anaesthetized with 5% isoflurane and euthanized by cervical dislocation.

### 2.3 Induction of murine myocardial infarction

Mice underwent a left anterior descending coronary artery ligation to produce a MI, or sham surgical procedure, as previously described.^12,18^ Briefly, mice were administered SR-buprenorphine preoperatively (single 50 μL dose of 1.0 mg/kg s.c.). The mice were then anaesthetized with isoflurane, intubated with a 20-gauge angiocatheter and ventilated, with anaesthesia maintained at 2.5% isoflurane in 100% oxygen with a flow rate of 0.5 L/min. Under sterile conditions, the thorax and pericardium were opened, and the left anterior descending coronary artery was permanently ligated with 7-0 prolene suture. In sham-operated controls, the thorax and pericardium were opened, but the left anterior descending coronary artery was not ligated. Following the procedure, the chest was closed and bupivicaine was used as a line block at the incision site (1–2 mg/kg; 0.1 ml volume infiltrating along the incision). The mice were extubated upon spontaneous respiration. Sm-Bmal1 KO and Cre-WT controls were tamoxifen treated 2 weeks prior to the MI or sham surgery.

### 2.4 Echocardiography

Echocardiographic measurements were collected in isoflurane-anaesthetized mice with a GE Healthcare (Mississauga, Canada) Vivid 7 Dimension ultrasound system (i13L 14 MHz linear-array transducer) or a VisualSonics (Toronto, Canada) Vevo 770 ultrasound system (30 MHz mechanical sector transducer), as previously described.^5,19^ Ventricular dimensions were measured in M-mode at the level of the papillary muscles. Echocardiographic measurements at week 1 assessed the initial level of cardiac injury; subsequent measurements (i.e. 4–8 weeks post-MI) assessed the decline in cardiac function due to infarct expansion.^17^

### 2.5 Pressure–volume hemodynamics

Pressure–volume hemodynamic measurements were collected in isoflurane-anaesthetized mice, as previously described.^17^ Briefly, a Transonic (Ithaca, USA) 1.2Fr pressure volume catheter was advanced into the left ventricle via an incision in the carotid artery. Left ventricular end diastolic pressure, left ventricular end systolic pressure, left ventricular end diastolic volume, left ventricular end systolic volume, stroke volume, heart rate, cardiac output, maximum and minimum first derivative of left ventricular pressure (d*P*/d*t*_max_; d*P*/d*t*_min_), systolic blood pressure (SBP) and diastolic blood pressure (DBP) were recorded using ADInstrument (Colorado Springs, USA) PowerLab and Lab Chart 7. MAP was subsequently calculated as DBP + (SBP−DBP)/3.

### 2.6 Cardiac histology

Following euthanization, hearts were perfused with saline containing 1 mol/L KCl and the fixed in 10% neutral buffered formalin for 24–48 h. Paraffin-embedded hearts were serially sectioned from apex to base, collecting ten 5 μm sections every 600 μm, and stained with Masson’s trichrome. Infarct expansion was calculated as the ratio of infarct length in relation to left ventricular circumference using Image J v1.48.^17,20^

### 2.7 Pressure myography

Mouse cremaster skeletal muscle resistance arteries were dissected from the cremaster muscle and cannulated onto micropipettes, as previously described.^5^ Myogenic responses (1–2 arteries per mouse) were elicited by step-wise 20 mmHg increases in transmural pressure from 20 to 100 mmHg. At each pressure step, vessel diameter (dia_active_) was measured once a steady state was reached. Following completion of all dia_active_ measurements, the buffer was replaced with Ca^2+^-free buffer and maximal passive diameter (dia_max_) was recorded at each pressure step. Myogenic tone was calculated as the percent constriction in relation to the maximal diameter at each transmural pressure: tone (% of dia_max_) = [(dia_max_−dia_active_)/dia_max_] × 100, where dia_active_ is the vessel diameter in Ca^2+^-containing buffer and dia_max_ is the diameter in Ca^2+^-free buffer. Analyses of KCl and phenylephrine-stimulated responses (measured at 60 mmHg transmural pressure) used the same calculation, only in this case, dia_active_ represents the vessel diameter at steady state following application of the given agent. Additional details are found in the Supplementary material online.

### 2.8 Reverse transcription-polymerase chain reaction

Cremaster skeletal muscle resistance artery RNA isolation, cDNA preparation, and quantitative PCR were completed as previously described.^21^ An analysis of hydroxymethylbilane synthase (HMBS) mRNA expression normalized to the geometric mean of glyceraldehyde 3-phosphate dehydrogenase (GAPDH) and glucose-6-phosphate dehydrogenase (G6PD) mRNA expression indicated that this housekeeping gene’s expression is stable over the circadian cycle and does not change following MI. Target genes, therefore, were normalized to HMBS. PCR primer information can be found in Supplementary material online, *Table S1*.

### 2.9 Western blotting

Western blots measuring pressure-stimulated ERK phosphorylation were conducted as previously described.^5^ Additional details and uncropped blots for all experiments are found in the Supplementary material online.

### 2.10 Statistics

All data are expressed as means ± standard error of the mean, where *n* represents the number of independent measures (e.g. arteries). Only male mice were used in this study to ensure appropriate comparability, as the Cre transgene is located on the Y-chromosome.^15^ Mice were randomly assigned into groups prior to interventions, without the use of an explicit randomization procedure. Using previous data as guidance, we calculated the experimental group sizes necessary to ensure that all data sets provide an 80% power level for the detection of the anticipated differences between groups with a two-tailed alpha level of 0.05. Data were statistically analysed with Graphpad Prism 9 software (Graphpad; San Diego, USA). Prior to completing statistical comparisons, we determined whether data were normally distributed (Shapiro–Wilk test) and had equal variances (*F*-test): data meeting both criteria were compared with parametric statistical tests (unpaired or paired Student’s *t*-test). In instances where data sets failed either the Shapiro–Wilk test or *F*-test, non-parametric statistical tests were utilized (Mann-Whitney or Wilcoxon tests). Supplementary material online, *Tables S2–S5* comprehensively summarize the statistical comparisons made in the present study and include mean and error measurements, group sizes, statistical test information, and *P* values. Circadian rhythms were identified using JTK_CYCLE (version 3.1) with R software (version 3.4.0), a non-parametric test designed to reliably identify rhythmicity.^22^ For all statistical tests utilized, differences were considered significant at *P* < 0.05.

## 3. Results

### 3.1 Cremaster skeletal muscle resistance artery myogenic reactivity is rhythmic

**Figure 1:**
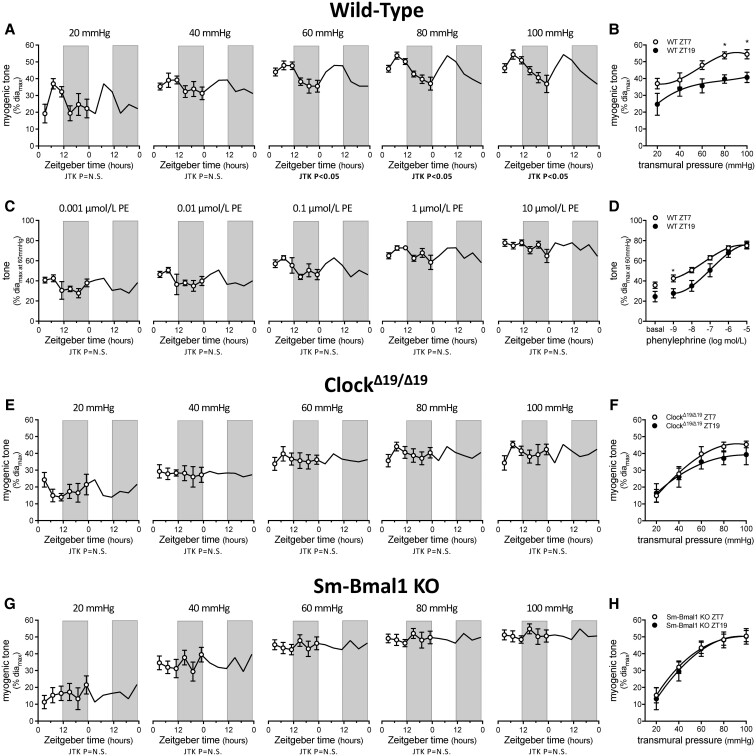
Myogenic responsiveness displays circadian rhythmicity. (*A*) Myogenic tone in wild-type (WT) cremaster arteries plotted over Zeitgeber time (*n* = 4–6) and (*B*) at ZT7 and ZT19 (*n* = 4–6). (*C*) Phenylephrine (PE)-stimulated vasoconstriction in WT cremaster arteries plotted over Zeitgeber time (*n* = 4–6) and (*D*) at ZT7 and ZT19 (*n* = 4–6). (*E*) Myogenic tone in Clock^Δ19/Δ19^ mutant cremaster arteries plotted over Zeitgeber time (*n* = 5–7) and (*F*) at ZT7 and ZT19 (*n* = 5–6). (*G*) Myogenic tone in Sm-Bmal1 KO cremaster arteries plotted over Zeitgeber time (*n* = 5–6) and (*H*) at ZT7 and ZT19 (*n* = 5–6). Data in *Panels A, C, E,* and *G* are double-plotted for visualization purposes and are statistically analysed for a circadian rhythm with JTK_Cycle. *Panels B, D, F,* and *H* are statistically analysed with *t*-tests or Mann–Whitney tests (unpaired single comparisons), with * denoting *P* < 0.05 at the respective transmural pressure or phenylephrine concentration.

Cremaster artery myogenic vasoconstriction is rhythmic at physiological pressures (60 mmHg and above), with a peak and trough at ZT7 and ZT19, respectively (*Figure 1A*, Supplementary material online, *Table S6*). This rhythm drives significant differences in myogenic tone at ZT7 vs. ZT19 (i.e. at the maximal and minimal reactivity observed, respectively; *Figure 1B*; passive diameters in Supplementary material online, *Figure S1A*). In contrast, neither phenylephrine-stimulated nor KCl-stimulated vasoconstriction possesses a significant rhythm (*Figure 1C*, Supplementary material online, *Figure S2* and Supplementary material online, *Table S6*). Of note, the phenylephrine dose-response relationship at ZT7 and ZT19 separates at the lower phenylephrine concentrations, due to higher, myogenically driven basal tone (*Figure 1D*).

To substantiate our observation that myogenic reactivity possesses a circadian rhythm, we recruited both global (Clock^Δ19/Δ19^ mutant)^16^ and smooth muscle cell-specific Bmal1 gene knockout (Sm-Bmal1 KO) molecular clock disruption models. Myogenic vasoconstriction is not rhythmic in cremaster arteries isolated from Clock^Δ19/Δ19^ or Sm-Bmal1 KO mice and consequently, no differences at ZT7 and ZT19 are observed (*Figure 1E–H*, Supplementary material online, *Table S7*; passive diameters in Supplementary material online, *Figure 1B* and *C*). Phenylephrine-stimulated vasoconstriction remains non-rhythmic in the clock disruption models and does not differ at ZT7 and ZT19 (Supplementary material online, *Figure S3* and Supplementary material online, *Table S7*). As expected, the companion assessments utilizing arteries isolated from tamoxifen-treated, non-floxed littermates expressing the Cre transgene (Cre-WT; controls for tamoxifen treatment and Cre activation in the Sm-Bmal1 KO model) recapitulated the observations in wild-type (WT) arteries (Supplementary material online, *Figure S4*, Supplementary material online, *Table S7*).

### 3.2 The molecular clock controls myogenic tone via the TNF mechanosensor signalosome

**Figure 2:**
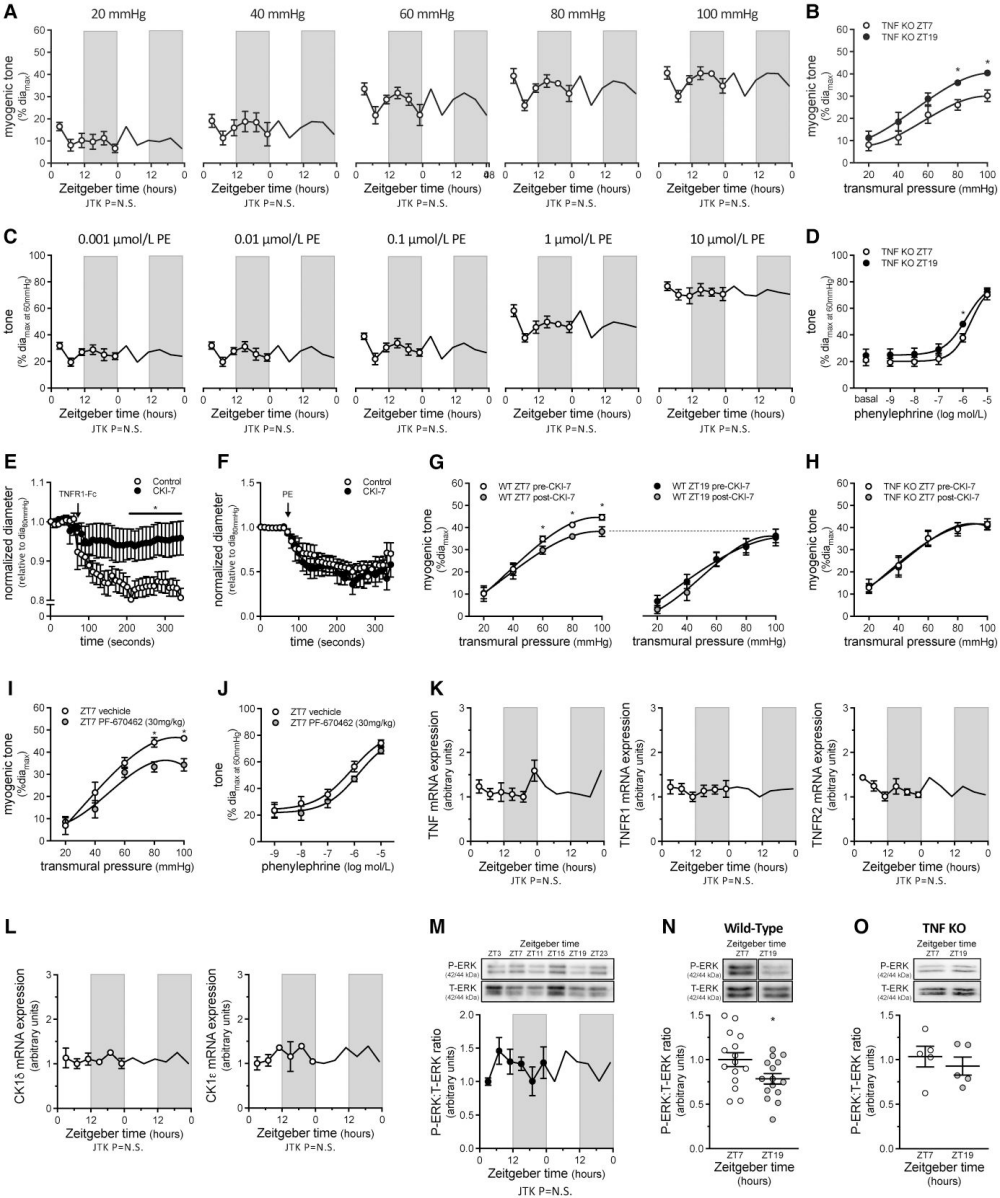
Rhythmic myogenic reactivity depends on TNF reverse signalling. (*A*) Myogenic tone in tumour necrosis factor knockout (TNF KO) cremaster arteries plotted over Zeitgeber time (*n* = 5–6) and (*B*) at ZT7 and ZT19 (*n* = 5–6). (*C*) Phenylephrine (PE)-stimulated vasoconstriction in TNF KO cremaster arteries plotted over Zeitgeber time (*n* = 5–6) and (*D*) at ZT7 and ZT19 (*n* = 5–6). (*E–G*) Effect of *in vitro* CK1 inhibition (10 nmol/L CKI-7, 30 min) on (*E*) sTNFR1-Fc stimulated vasoconstriction (*n* = 4–5), (*F*) phenylephrine-stimulated vasoconstriction (*n* = 5) and (*G*) myogenic tone at ZT7 (*n* = 6) and ZT19 (*n* = 5) in wild-type cremaster arteries. (*H*) Effect of *in vitro* CK1 inhibition on myogenic tone in TNF KO cremaster arteries at ZT7 (*n* = 5). (*I–J*) Effect of *in vivo* CK1 inhibition (30 mg/kg PF670462 i.p.; 24 h) on cremaster artery (*I*) myogenic tone and (*J*) phenylephrine-stimulated vasoconstriction (both *n* = 5; both at ZT7). (*K*) TNF, TNFR1, and TNFR2 mRNA expression (normalized to HMBS mRNA expression) in cremaster arteries plotted over Zeitgeber time (*n* = 5–7 for each point). (*L*) CK1 delta and CK1 epsilon mRNA expression (normalized to HMBS mRNA expression) in cremaster arteries plotted over Zeitgeber time (*n* = 2 for each point). (*M*) pressure-stimulated (40–100 mmHg) ERK1/2 phosphorylation levels in wild-type cremaster arteries plotted over Zeitgeber time (*n* = 5). (*N* and *O*) ERK1/2 phosphorylation levels at ZT7 and ZT19 in (*N*) wild-type (*n* = 15) and (*O*) TNF KO cremaster arteries (*n* = 5). Data in *Panels A, C, K, L,* and *M* are double-plotted for visualization purposes and are statistically analysed for a circadian rhythm with JTK_Cycle. *Panels B, D, E, F, I,* and *J* are statistically analysed as unpaired single comparisons using *t*-tests or Mann–Whitney tests, with * denoting *P* < 0.05. *Panels G, H, N,* and *O* are statistically analysed as paired single comparisons using paired *t*-tests or Wilcoxon tests, with * denoting *P* < 0.05.

Our previous work demonstrates that mTNF is the primary mechanosensor mediating myogenic reactivity in cremaster skeletal muscle resistance arteries.^5^ In arteries isolated from TNF KO mice, neither myogenic nor phenylephrine-stimulated vasoconstriction is rhythmic (*Figure 2A–D*; Supplementary material online, *Table S7*; passive diameters in Supplementary material online, *Figure 1D*). Intriguingly, ZT7 displays markedly lower myogenic tone than at the other time points, yielding an apparent ‘inversion’ of the normal ZT7/ZT19 myogenic tone relationship (*Figure 2B*). The molecular clock genes Bmal1, Per2 and Clock all cycle in TNF KO cremaster arteries (Supplementary material online, *Figure S5*; Supplementary material online, *Table S8*). Intriguingly, Bmal1 and Per2 exhibit a small (∼2 h) phase shift and an increase in rhythm amplitude, while Clock exhibits a phase shift that pushes it out of alignment with Bmal1 (Supplementary material online, *Figure S5*; Supplementary material online, *Table S8*). While these modest alterations to the molecular clock probably do not explain the ‘inversion’ of myogenic reactivity at ZT7/ZT19 in TNF KO arteries, they do suggest that TNF may be involved in vascular smooth muscle cell peripheral clock entrainment, which is consistent with a previous report documenting a TNF-dependent phase shift and amplitude change in astrocyte Per2 expression.^23^

The signalosome constituents that mediate mTNF reverse signalling are not well defined: to date, casein kinase 1 (CK1) is the only signalling entity postulated to interact mTNF’s intracellular domain in this context.^24,25^ In WT cremaster skeletal muscle resistance arteries, the pan-CK1 inhibitor CKI-7 (10 nmol/L; 30 min) abolishes mTNF reverse signalling-dependent vasoconstriction stimulated using the intrinsically active soluble TNF-receptor (TNFR) 1 fusion protein construct (sTNFR1-Fc; *Figure 2E*);^5^ phenylephrine-stimulated vasoconstriction, which is mTNF-independent, is not affected (*Figure 2F*). At ZT7, CKI-7 attenuates pressure-stimulated myogenic vasoconstriction, reducing it to a level comparable to that at ZT19 (*Figure 2G*); increasing the CKI-7 concentration 100-fold (i.e. to 1 μM) does not further attenuate myogenic reactivity (Supplementary material online, *Figure S6*). In contrast, 10 nmol/L CKI-7 does not affect myogenic reactivity at ZT19 in WT arteries (*Figure 2G*), nor does it attenuate myogenic tone at ZT7 in TNF KO cremaster arteries (*Figure 2H*).

*In vivo*, CK1 inhibition (single dose of 30 mg/kg PF670462 i.p. administered at ZT10–ZT10.5 the previous day; PF670462 primarily inhibits the CK1 delta and epsilon isoforms) attenuates myogenic vasoconstriction at ZT7, reducing it to a level comparable to that of ZT19 (*Figure 2I*; compare to *Figure 2G*); *in vivo* PF670462 treatment does not alter phenylephrine-stimulated vasoconstriction (*Figure 2J*). Escalating the PF670462 dose to 50 mg/kg does not further attenuate of myogenic reactivity (Supplementary material online, *Figure S7*). Based on the attenuating effects of TNF gene deletion and PF670462 *in vivo*, we assessed TNF, TNFR1, TNFR2 CK1 delta and CK1 epsilon mRNA expression for rhythmicity: none of these elements are rhythmic at the mRNA level in WT cremaster arteries (*Figure 2K and L*, Supplementary material online, *Table S8*), suggesting that the time-of-day variation in myogenic tone may be mediated through post-transcriptional mechanisms.

In skeletal muscle resistance arteries, ERK1/2 is an important myogenic signalling element that connects the mechanically stimulated signals to calcium-sensitization processes via the activation sphingosine kinase 1.^5,26^ Although it is not known whether ERK1/2 directly complexes within the mTNF signalosome, the attenuating effects of ERK inhibition on myogenic tone and sTNFR1-Fc-stimulated constriction clearly demonstrate its important role within the overall myogenic signalling cascade.^5^ Consistent with our previous observations,^5^ pressure elevation clearly stimulates ERK1/2 phosphorylation in cremaster arteries (Supplementary material online, *Figure S8*). When assessed over the diurnal cycle (*Figure 2M*), pressure-stimulated ERK1/2 phosphorylation is significantly higher at ZT7 compared to ZT19 (*Figure 2N*); however, the pattern does not possess a statistically significant 24 h rhythm (*Figure 2M*; JTK_Cycle Bonferroni adjusted *P* = 1.000). In TNF KO cremaster arteries, no difference in ERK1/2 phosphorylation is observed between ZT7 and Z19 (*Figure 2O*). CK1 inhibition also eliminates the time-of-day difference in ERK1/2 phosphorylation, although our experiments did not control for potential baseline effects of PF-670462 at 40 mmHg (Supplementary material online, *Figure S9*).

Collectively, our mechanistic data show that: (i) based on CK1 inhibitor sensitivity, only the oscillatory proportion of myogenic tone is mediated by mTNF reverse signalling and (ii) post-translational mechanisms likely underlie the functional rhythm in myogenic reactivity. mTNF reverse signalling appears to modulate ERK1/2 phosphorylation; however, ERK1/2 phosphorylation does not align with vasoconstriction levels in the TNF KO model.

### 3.3 Myocardial infarction disrupts the circadian rhythmicity in myogenic reactivity

**Figure 3:**
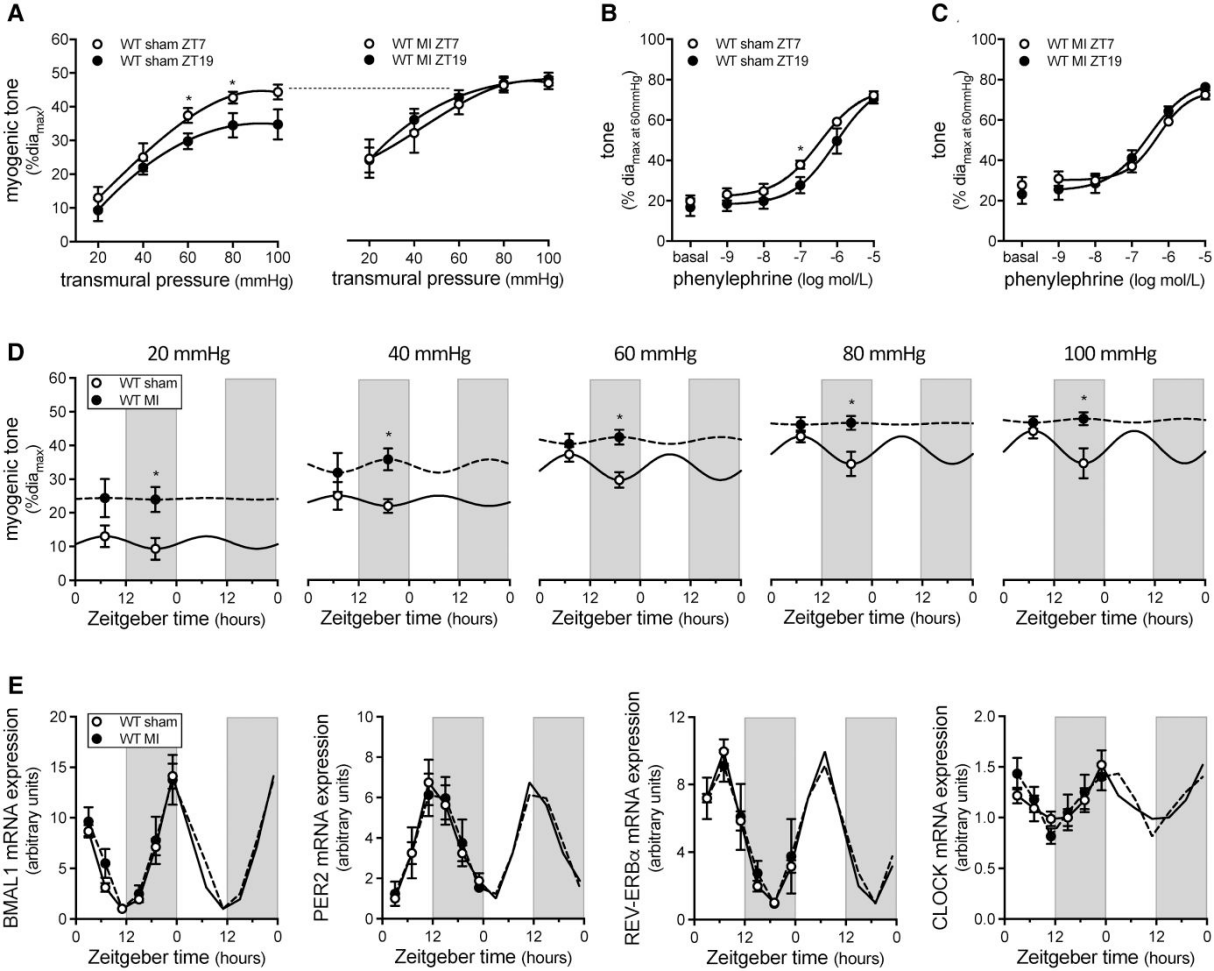
Myogenic rhythmicity is altered following myocardial infarction. (*A*) Myogenic tone in wild-type (WT) cremaster arteries isolated at ZT7 and ZT19 from sham-operated mice (*n* = 6–9) and mice with a myocardial infarction (MI; *n* = 7). Phenylephrine-stimulated vasoconstriction in cremaster arteries isolated at ZT7 and ZT19 from (*B*) sham-operated mice (*n* = 6–9) and (*C*) MI mice (*n* = 7). (*D*) Myogenic tone in sham-operated and MI mice plotted over Zeitgeber time (*n* = 6–9). (*E*) Bmal1, Per2, Rev-Erbα and Clock mRNA expression (normalized to HMBS mRNA expression) in cremaster arteries from sham and MI mice plotted over Zeitgeber time (*n* = 3–4). *Panels A-D* are statistically analysed as unpaired single comparisons using *t*-tests or Mann–Whitney tests, with * denoting *P* < 0.05. Data in *Panels D and E* are double-plotted for visualization purposes; data in *Panel E* are statistically analysed for a circadian rhythm with JTK_Cycle.

We have previously shown that resistance arteries from mice with a MI possess augmented cremaster artery myogenic tone that correlates with increased TPR.^5,12^ We therefore hypothesized that the MI ‘locks’ myogenic tone at an elevated level, thereby eliminating rhythmicity. Indeed, while myogenic vasoconstriction at ZT7 vs. ZT19 differs in arteries from sham mice, it is similar for both time points in arteries from mice with a MI (*Figure 3A*, passive diameters in Supplementary material online, *Figure S10*). Phenylephrine-stimulated vasoconstriction is similar at ZT7 and ZT19 in arteries from sham and MI mice (*Figure 3B and C*); the curves separate slightly in sham arteries, due to differences in basal tone (*Figure 3B*). Cremaster arteries from sham and MI mice display similar myogenic tone at ZT7: thus, the MI appears to specifically affect myogenic tone at ZT19 and augment it to the ZT7 level (*Figure 3A and D*). The loss of myogenic rhythmicity in the MI model is not attributable to perturbed molecular clock function, as *Bmal1*, *Per2*, *Rev-Erbα* and *Clock* gene mRNA expression rhythms are similar in cremaster arteries from sham and MI mice (*Figure 3E*, Supplementary material online, *Table S9*), as well as naïve mice (Supplementary material online, *Figure S5A–C* and Supplementary material online, *Table S8*). Furthermore, MI does not alter the mRNA expression of known mTNF reverse signalling components (mTNF, TNF receptors 1 and 2 and CK1; Supplementary material online, *Figure S11*). Thus, MI affects the link between the molecular clock and the myogenic mechanism, resulting in a myogenic tone level that is ‘locked’ at the circadian maximum.

### 3.4 Global Clock^Δ19/Δ19^ mutation improves cardiac function in heart failure

**Figure 4:**
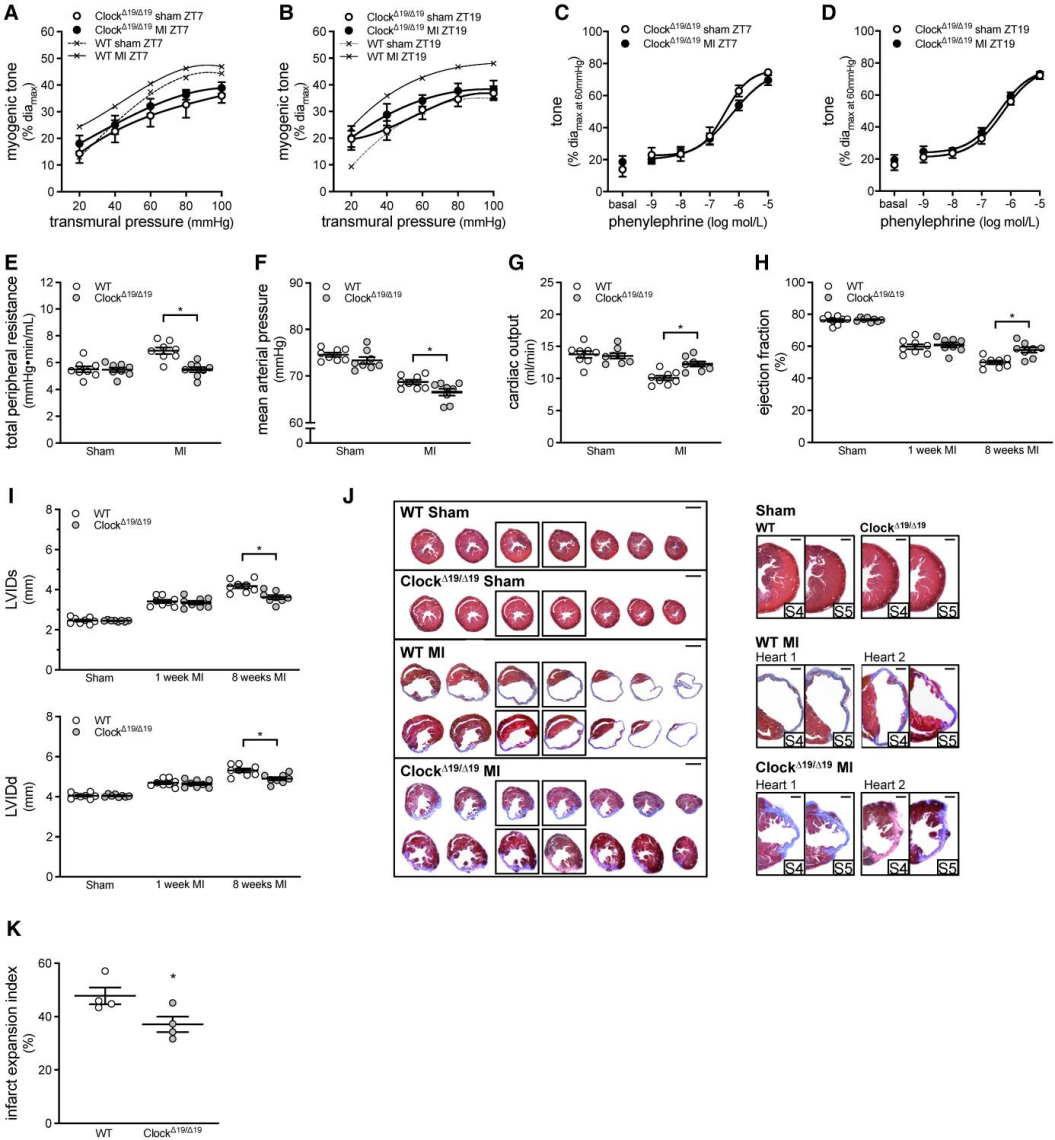
Global Clock^Δ19/Δ19^ mutation attenuates myogenic responsiveness and improves cardiac function following myocardial infarction. Myogenic tone at (*A*) ZT7 (*n* = 7–8) and (*B*) ZT19 (*n* = 7–8) in Clock^Δ19/Δ19^ cremaster arteries isolated from sham-operated mice and mice with a myocardial infarction (MI). Traces from sham and MI wild-type (WT) mice are reproduced from *Figure 3A* for qualitative comparison purposes. Phenylephrine-stimulated vasoconstriction at (*C*) ZT7 (*n* = 7–8) and (*D*) ZT19 (*n* = 7–8) in Clock^Δ19/Δ19^ cremaster arteries isolated from sham-operated and MI mice. Pressure–volume hemodynamic assessments of (*E*) total peripheral resistance, (*F*) mean arterial pressure, and (*G*) cardiac output in WT and Clock^Δ19/Δ19^ mutant mice with sham or MI procedure (*n* = 8). Echocardiographic assessments of (*H*) ejection fraction and (*I*) systolic and diastolic left ventricular internal diameter (LVIDs and LVIDd) in WT and Clock^Δ19/Δ19^ mutant mice with sham or MI procedure (*n* = 8). (*J*) Cardiac histology was processed at 8 weeks post-MI. Shown are representative histological images of cardiac infarcts in WT and Clock^Δ19/Δ19^ mutant mice with sham or MI procedure; serial sections are shown on the left (bar = 2 mm) and close-ups of the infarct region in serial sections 4–5 are shown on the right (bar = 500 µm). (*K*) Quantification of infarct expansion from the histological images (*n* = 4). All data are statistically analysed as unpaired single comparisons using *t*-tests or Mann–Whitney tests, with * denoting *P* < 0.05.

In Clock^Δ19/Δ19^ mutant mice, a model that globally disrupts the circadian clock (Supplementary material online, *Figure S12*),^16^ myogenic tone is suspended at the circadian minimum (i.e. similar sham levels of myogenic tone at ZT7 and ZT19 *Figure 4A and B*). MI fails to augment cremaster artery myogenic tone at either ZT7 or ZT19 in Clock^Δ19/Δ19^ mutant mice; phenylephrine responses are unaffected (*Figure 4A–D*, passive diameters in Supplementary material online, *Figure S13*). TPR is reduced in Clock^Δ19/Δ19^MI mice relative to WT MI controls, with a concomitant improvement in cardiac output; MAP is marginally, but significantly reduced (*Figure 4E–G* and Supplementary material online, *Table S10*). The improved cardiac output is attributable to increased stroke volume, rather than a higher heart rate (Supplementary material online, *Table S10*).

Since global clock disruption potentially influences cardiac cell responses to injury,^27^ we confirmed that ejection fraction and fractional shortening are similarly affected in WT and Clock^Δ19/Δ19^ mice at 1 week post-MI (*Figure 4H* and Supplementary material online, *Table S10*). By 8 weeks post-MI, these parameters have diverged, declining in WT, but not in Clock^Δ19/Δ19^ mice (*Figure 4H* and Supplementary material online, *Table S10*). Structural observations paralleled these functional parameters: systolic and diastolic left ventricle diameters/volumes at 1 week post-MI are comparable in WT and Clock^Δ19/Δ19^ hearts, but these parameters also diverge by 8 weeks post-MI, indicating that further ventricle enlargement is strongly attenuated in Clock^Δ19/Δ19^ hearts (*Figure 4I* and Supplementary material online, *Table S10*). At the histological level, infarct expansion is significantly less in Clock^Δ19/Δ19^ hearts relative to WT controls (*Figure 4J–K*).

### 3.5 Smooth muscle-specific Bmal1 deletion improves cardiac function in heart failure

**Figure 5:**
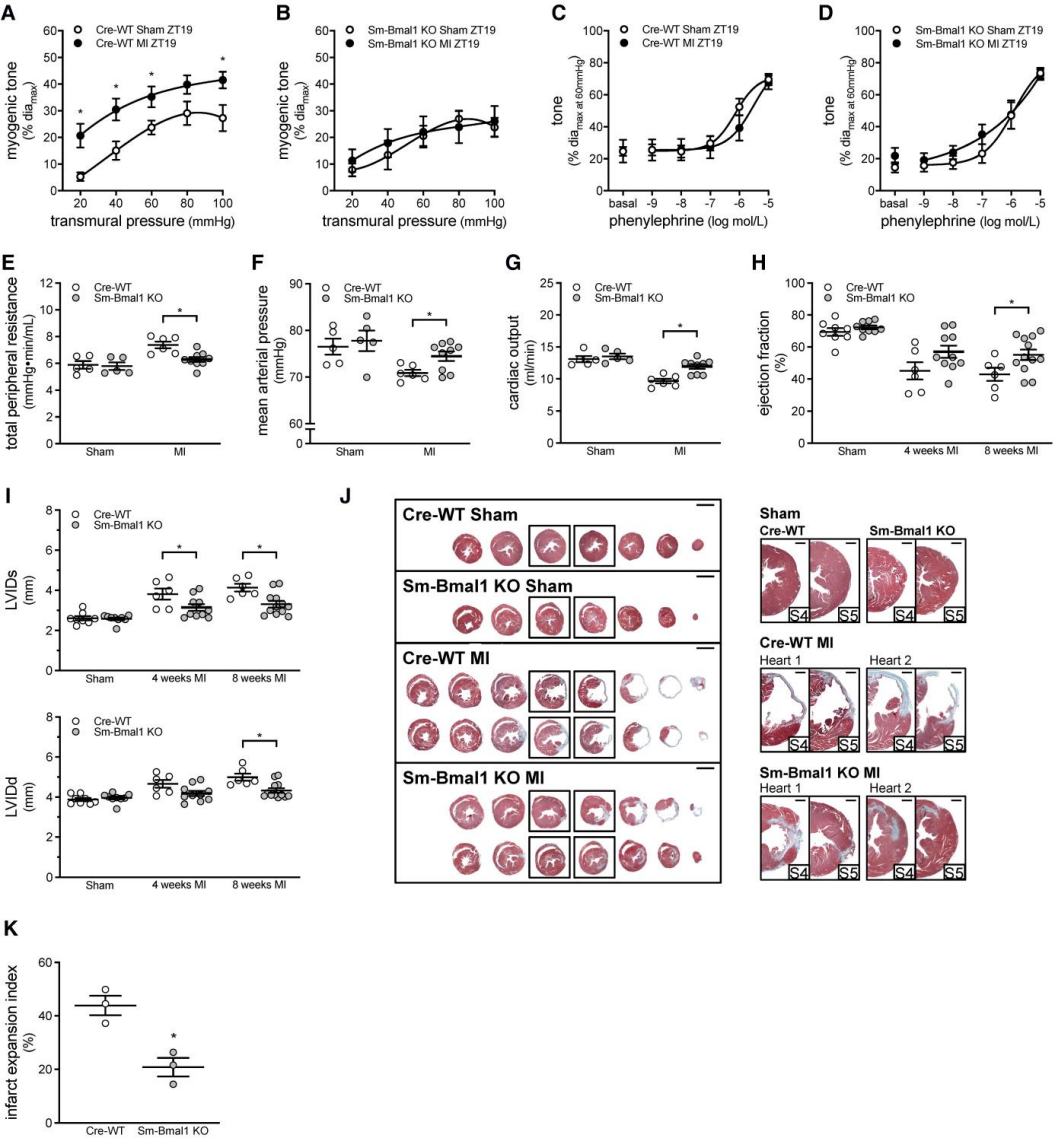
Smooth muscle Bmal1 deletion attenuates myogenic responsiveness and improves cardiac function following myocardial infarction. Myogenic tone at ZT19 in (*A*) Cre-WT (*n* = 7) and (*B*) Sm-Bmal1 KO (*n* = 7) cremaster arteries isolated from sham-operated mice and mice with a myocardial infarction (MI). Phenylephrine-stimulated vasoconstriction at ZT19 in (*C*) Cre-WT (*n* = 7) and (*D*) Sm-Bmal1 KO (*n* = 7) cremaster arteries isolated from sham-operated and MI mice. Pressure–volume hemodynamic assessments of (*E*) total peripheral resistance, (*F*) mean arterial pressure and (*G*) cardiac output in Cre-WT and Sm-Bmal1 KO mice with sham or MI procedure (*n* = 5–9). Echocardiographic assessments of (*H*) ejection fraction and (*I*) systolic and diastolic left ventricular internal diameter (LVIDs and LVIDd) in Cre-WT and Sm-Bmal1 KO mice with sham or MI procedure (*n* = 6–12). (*J*) Cardiac histology was processed at 8 weeks post-MI. Shown are representative histological images of cardiac infarcts in Cre-WT and Sm-Bmal1 KO mice with sham or MI procedure; serial sections are shown on the left (bar = 2 mm) and close-ups of the infarct region in serial sections 4–5 are shown on the right (bar = 500 µm). (*K*) Quantification of infarct expansion from the histological images (*n* = 3). All data are statistically analysed as unpaired single comparisons using *t*-tests or Mann–Whitney tests, with * denoting *P* < 0.05.

As in Clock^Δ19/Δ19^ mutant mice, the MI-induced myogenic tone augmentation at ZT19 is absent in cremaster arteries isolated from Sm-Bmal1 KO mice; phenylephrine responses are unaffected (*Figure 5A–D*; passive diameters in Supplementary material online, *Figure S14*). This indicates that smooth muscle molecular clock disruption eliminates the mechanism underlying the augmented myogenic tone at ZT19 post-MI. TPR is reduced in Sm-Bmal1 KO MI mice relative to Cre-WT controls, with concomitant increases in MAP and cardiac output (*Figure 5E–G* and Supplementary material online, *Table S11*). The higher cardiac output is attributable to enhanced ventricular function, as stroke volume, ejection fraction, and fractional shortening are increased, while heart rate is unaltered (*Figure 5H* and Supplementary material online, *Table S11*). At the structural level, both systolic and diastolic left ventricular diameters/volumes are reduced in Sm-Bmal1 KO hearts at 8 weeks post-MI, compared to Cre-WT sham controls (*Figure 5I* and Supplementary material online, *Table S11*). Improved ventricular remodelling is evident at the histological level (*Figure 5J*): compared to Cre-WT controls, Sm-Bmal1 KO hearts display reduced left ventricular size and decreased infarct expansion (by 53%, *Figure 5K*). Together, these data confirm that the Sm-Bmal1 KO vascular phenotype exerts beneficial effects on cardiac performance and remodelling following infarction.

## 4. Discussion

This investigation demonstrates that myogenic reactivity in skeletal muscle resistance arteries is rhythmic and aligns with the well-known rhythm in TPR.^9^ Following MI, myogenic responsiveness is uncoupled from circadian control and locked at its circadian maximum, although the core circadian clock appears to function normally. Disrupting the forward loop of the core circadian clock (Clock Δ19 mutation or Sm-Bmal1 KO) has two key effects in addition to abolishing myogenic rhythmicity: it suspends myogenic tone at its circadian minimum and prevents the augmentation of myogenic tone post-MI. This latter effect associates with reduced TPR and cardiac injury. We conclude that the circadian mechanism modulating myogenic reactivity in skeletal muscle resistance arteries represents a potential therapeutic target for improving cardiac outcome post-MI.

The circadian rhythms in myogenic reactivity and TPR peak during the rest/sleep phase and trough during the active/awake phase, an arrangement that is anti-phase to the circadian rhythm in cardiac output.^9^ This ensures that higher levels of blood flow are delivered when metabolic activity is generally high and it mitigates the reduction in MAP when cardiac output drops during sleep. Our data show that at physiological pressures (80 and 100 mmHg), only ∼25% of myogenic tone is rhythmic (i.e. the amplitude of the rhythm in WT arteries is ∼25% of total myogenic tone; *Figure 1A and B*). The fact that only a portion of myogenic reactivity is under circadian control has important implications: it defines a dynamic range of myogenic tone modulation and implies that a constitutive level of myogenic reactivity (i.e. the remaining 75%) is maintained for acceptable hemodynamic control.

**Figure 6:**
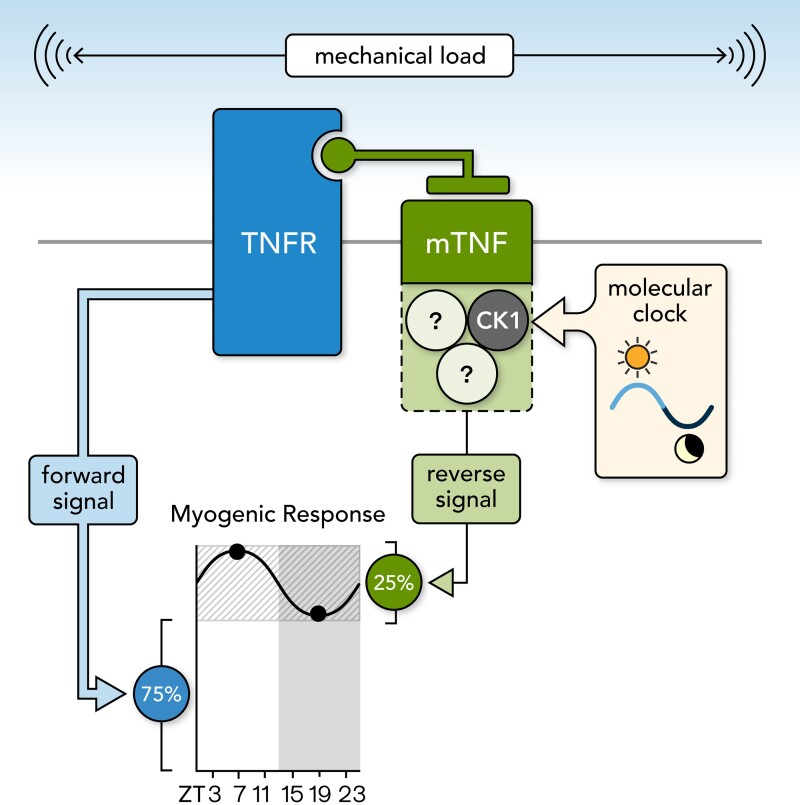
Proposed mechanotransduction mechanism mediating myogenic reactivity in skeletal muscle resistance arteries. Shown is the proposed molecular mechanism regulating myogenic responsiveness in skeletal muscle resistance arteries. Membrane-bound tumour necrosis factor (mTNF) and a tumour necrosis factor receptor (TNFR) form a mechanosensitive pair. Upon mechanical stimulation, both mTNF and TNFR generate signals; the signal generated by TNFR regulates a larger proportion of myogenic tone than mTNF (∼75% vs. 25%) and is not rhythmic. The mTNF signalosome incorporates CK1 as a pivotal element for the propagation of the mTNF-dependent reverse signal; the mTNF signalosome also incorporates a yet undefined element that is under circadian control. Accordingly, the mTNF reverse signal and the ∼25% of the myogenic response that it controls display circadian rhythmicity.

Our previous work demonstrates that mTNF is the critical mechanosensor initiating myogenic reactivity in cremaster skeletal muscle resistance arteries.^5^ Our original model presumed that the mTNF reverse signal was the sole contributor to the myogenic signal;^5^ however, our present data reveal a discrepancy that can only be explained by the addition of a second component to the model (*Figure 6*). Specifically, mTNF reverse signals stimulated by sTNFR1-Fc are abolished following CK1 inhibition, while myogenic responses are only modestly affected (∼25% attenuation). The most reasonable explanation for this discrepancy is the presence of a CK1 insensitive, TNFR-dependent signal. By extension, our data suggest that (i) only the mTNF reverse signal is under circadian control and (ii) the mTNF reverse signal is not ‘an equal partner’ in the overall myogenic signal: it only mediates the variable ∼25% proportion of myogenic tone (*Figure 6*). This latter aspect would explain why the genetic manipulation of the molecular clock and the pharmacological inhibition of CK1 attenuated myogenic tone, without abolishing it.

TPR elevation is considered a critical compensatory response that maintains MAP following cardiac injury.^1,2^ It is not surprising, therefore, that augmented microvascular constriction is observed in multiple vascular beds in this pathological setting.^12,28,29^ The present study focused on skeletal muscle resistance arteries, because collectively, the skeletal muscle microcirculation is a prominent contributor to TPR. To our surprise, myogenic reactivity following MI is not universally augmented throughout the circadian cycle: instead, myogenic tone is ‘locked’ at its circadian maximum, yielding a TPR profile that is presumably normal in the rest/sleep phase and elevated in the active/awake phase. This focuses the pathological TPR elevation to the timeframe when cardiac output is at its highest levels.^10,11^ Non-myogenic constriction mechanisms in skeletal muscle resistance arteries (i.e. phenylephrine and KCl) are not under circadian control and are not enhanced following MI. Thus, the molecular clock in skeletal muscle resistance arteries modulates an element unique to myogenic signalling and MI pathologically overrides this control mechanism.

TNF’s cytoplasmic domain does not possess discernible enzymatic function and hence, almost certainly signals through associated proteins. However, the signalosome constituents that mediate mTNF reverse signalling are not well defined: CK1 is the only presently known interactor within the mTNF/TNFR complex.^24,25^ Our analyses indicate that none of these signalling elements display a rhythm in mRNA expression, nor are they altered in response to MI. Thus, either (i) an unknown signalosome constituent is under circadian transcriptional control or (ii) the activities and/or localization of critical kinases/phosphatases that regulate signalosome components possess a circadian pattern.^24^ On this note, our data favour the latter mechanism, as CK1 inhibition exhibits a clear phase sensitivity (i.e. attenuates myogenic reactivity at ZT7, but not at ZT19), despite neither CK1 delta nor CK1 epsilon oscillating at the mRNA level. Both CK1 delta and CK1 epsilon have well-known circadian rhythms in subcellular localization:^30^ it is therefore tempting to speculate that oscillations in subcellular localization dictate CK1’s availability to complex with the mTNF signalosome at the plasma membrane, thereby yielding a ‘functional rhythm’.

We have previously shown that mTNF reverse signalling stimulates ERK1/2 phosphorylation.^5^ Since ERK is an important mediator of myogenic signalling,^26^ we hypothesized that pressure-stimulated ERK1/2 phosphorylation would be rhythmic and align with the magnitude of myogenic tone. Indeed, a clear ZT7/ZT19 differential in pressure-stimulated ERK phosphorylation is evident, despite its failure to display a statistically significant 24 h rhythm according to JTK_Cycle analysis. While the ZT7/ZT19 phosphorylation differential is lost following CK1 inhibition and in the TNF KO model, the comparison of WT and TNF KO arteries demonstrates that ERK phosphorylation does not always associate with myogenic tone levels. Thus, the relationship between ERK phosphorylation and vasoconstriction is more complicated than a simple cause/effect model, presumably because it integrates unrelated inputs in addition to myogenic signals.

The MI-induced myogenic tone augmentation is absent in both Sm-Bmal1 KO and Clock^Δ19/Δ19^ mice, indicating that the mechanism underlying the MI-associated increase requires an intact molecular clock. In fact, molecular clock disruption has the opposite vascular effect of the MI pathology: it suspends myogenic reactivity at its circadian minimum, rather than at the circadian maximum. This vascular phenotype associates with reduced TPR in mice with an MI, as well as beneficial effects on cardiac morphology and function. Reduced cardiac afterload plausibly explains the association between TPR and cardiac benefit; however, this is not the only possible vascular explanation. For example, if coronary artery myogenic tone would parallel the skeletal muscle artery phenotype, then the improved outcome could, at least in part, be attributable to better cardiac perfusion, presumably in at-risk regions (i.e. the infarct penumbra). We do not favour this latter explanation, as Karam *et al.* provide evidence that (i) coronary resistance *decreases*, (ii) coronary blood flow *increases,* and (iii) the coronary flow reserve is not exhausted, indicating that perfusion can further increase.^31^

Our investigation identifies a fundamentally new means of modulating vascular resistance. This mechanism incorporates two potential signalling systems that could be targeted to reduce TPR following MI: mTNF reverse signalling, which is under circadian control, and the smooth muscle cell peripheral molecular clock itself. Targeting these signalling mechanisms would preserve the residual, clock-independent component of myogenic responsiveness that maintains adequate levels of autoregulatory capacity and TPR regulation: this would confer a degree of safety to the intervention, since the therapeutic maximum does not threaten essential hemodynamic control. Presently, there is no means to target the molecular clock or TNF reverse signalling in smooth muscle cells without systemic, off-target effects.

There are several caveats to the present study that must be acknowledged. Animal models and experimental conditions are tightly controlled in order to generate clear conclusions; however, the translational application to humans is far more heterogenous and complex. It is also important to recognize that our investigation only demonstrates *an association* between skeletal muscle vascular tone and infarct severity and not a causal relationship. Therefore, future studies will need to contribute additional confirmatory evidence to this association. It is very likely that the circadian control of skeletal muscle resistance arteries in humans is conserved: (i) as observed in mice, TPR in humans displays a circadian rhythm that peaks in the rest/sleep phase^32^ and (ii) the mTNF reverse signalling mechanism is present in human skeletal muscle resistance arteries.^5^ Given the ubiquitous and conserved nature of the molecular clock, targeting the mTNF reverse signalling mechanism has a much better prospect yielding more specific effects; however, mTNF reverse signalling mechanism is not defined sufficiently enough to allow this. Thus, the pharmacological approaches to modulate this mechanism are limited at present. It may be possible to clinically target the molecular clock or CK1 in the near future, as agents manipulating these entities are currently in clinical trials: in this regard, both we (global Clock mutant) and others^33^ show that pharmacologically targeting the circadian system appears to be well-tolerated.

In summary, the smooth muscle cell circadian clock controls a discrete portion of myogenic tone in resistance arteries that prominently regulate TPR. Pathological signalling following MI commandeers the link between the molecular clock and myogenic signalling and locks myogenic tone at its circadian peak. Circadian clock disruption prevents the MI-induced increase in myogenic reactivity, TPR and cardiac afterload that associates with a substantial positive impact on cardiac function, infarct expansion and remodelling. These results identify the molecular intersection between the circadian clock and myogenic signalling as a possible therapeutic target for minimizing cardiac injury following infarction. Our data also confirm that altered microvascular reactivity drives significant infarct expansion post-MI and consequently, meaningfully impacts clinical outcome.

## Supplementary material

Supplementary material is available at *Cardiovascular Research* online.

## Author contributions

J.T.K., D.L., and S.S.B. designed the experimental plan. J.T.K., D.L., F.J.A., C.J.R., H.Z., D.D.D., J.H., and T.N.K. collected and analysed data. S.P.H., T.A.M., and S.S.B. supervised data collection and provided intellectual expertise. J.T.K., D.L., and S.S.B. prepared the manuscript; all authors reviewed and edited the manuscript.

## Supplementary Material

cvac174_Supplementary_Data

## Data Availability

The data that support the findings of this study are available within the article and its supplemental information files. All data can be made available by the corresponding author upon request.

## Notes

Translational perspective A portion of peripheral vascular tone and hence, TPR, is under circadian control. MI preferentially augments vascular tone in the active phase, when the heart works hardest to provide adequate tissue perfusion. Disrupting the circadian clock reduces peripheral resistance and is associated with improved cardiac function and reduced infarct expansion post-MI. The intersection between the circadian clock and myogenic signalling could be a potential therapeutic target to manage peripheral resistance and improve cardiac outcome following an infarction. This mechanism may be safer than current vasodilators, since the therapeutic maximum does not threaten essential hemodynamic control.

## Abbreviations

CK1: casein kinase 1
MAP: mean arterial pressure
MI: myocardial infarction
TPR: total peripheral resistance
TNF: tumour necrosis factor
TNFR: tumour necrosis factor receptor
WT: wild-type
ZT: Zeitgeber time
