# Supplementary material for: Disrupting circadian control of peripheral myogenic reactivity mitigates cardiac injury following myocardial infarction
Source: Cardiovasc Res. 2022 Nov 22;119(6):1403–15. doi: 10.1093/cvr/cvac174 (PMC10262184; doi:10.1093/cvr/cvac174)
Supplement: cvac174_Supplementary_Data [file cvac174_supplementary_data.pdf]

# Supplemental Material

To accompany the manuscript:

## Disrupting circadian control of peripheral myogenic reactivity mitigates cardiac injury following myocardial infarction

Supplemental Methods ........ Page 2

Supplemental Figure 1 ......... Page 3
Supplemental Figure 2 ......... Page 4
Supplemental Figure 3 ......... Page 5
Supplemental Figure 4 ......... Page 6
Supplemental Figure 5 ......... Page 7
Supplemental Figure 6 ......... Page 8
Supplemental Figure 7 ......... Page 9
Supplemental Figure 8 ......... Page 10
Supplemental Figure 9 ......... Page 11
Supplemental Figure 10 ....... Page 12
Supplemental Figure 11 ....... Page 13
Supplemental Figure 12 ....... Page 14
Supplemental Figure 13 ....... Page 15
Supplemental Figure 14 ....... Page 16
Supplemental Figure 15 ....... Page 17
Supplemental Figure 16 ....... Page 18
Supplemental Figure 17 ....... Page 19

Supplemental Table 1 .......... Page 20
Supplemental Table 2 .......... Pages 21-22
Supplemental Table 3 .......... Page 23
Supplemental Table 4 .......... Page 24-25
Supplemental Table 5 .......... Page 26
Supplemental Table 6 .......... Page 27
Supplemental Table 7 .......... Page 28
Supplemental Table 8 .......... Page 29
Supplemental Table 9 .......... Page 30
Supplemental Table 10......... Page 31
Supplemental Table 11......... Page 32

## Supplemental Methods

***Pressure myography.*** Mouse cremaster skeletal muscle resistance arteries were dissected from the cremaster muscle and cannulated onto micropipettes. The isolation procedure and myography experiments utilized calcium-containing 3-morpholinopropanesulfonic acid (MOPS) buffered saline containing [mmol/L]: NaCl 147.0, KCl 4.7, $CaCl_2$ 1.5, $MgSO_4$ 1.2, $NaH_2PO_4$ 1.2, pyruvate 2.0, EDTA 0.02, MOPS 3.0 and glucose 5.0. Following cannulation, arteries were heated to 37°C and stretched to their *in vivo* lengths. Vessel viability as assessed with 10 μmol/L phenylephrine: vessels that developed >50% tone were considered viable. Prior to myogenic tone measurements, vasoconstriction to 60 mmol/L KCl was assessed, followed by a phenylephrine dose-response assessment (1 nmol/L to 10 μmol/L).

Myogenic responses were elicited by step-wise 20 mmHg increases in transmural pressure from 20 mmHg to 100 mmHg. At each pressure step, vessel diameter ($dia_{active}$) was measured once a steady state was reached. Following completion of all $dia_{active}$ measurements, the MOPS buffer was replaced with a $Ca^{2+}$-free version and maximal passive diameter ($dia_{max}$) was recorded at each pressure step. Myogenic tone was calculated as the percent constriction in relation to the maximal diameter at each transmural pressure: tone (% of $dia_{max}$) = [($dia_{max}$-$dia_{active}$)/$dia_{max}$]x100, where $dia_{active}$ is the vessel diameter in MOPS containing $Ca^{2+}$ and $dia_{max}$ is the diameter in $Ca^{2+}$-free MOPS. Analyses of KCl and phenylephrine-stimulated responses (measured at 60 mmHg transmural pressure) used the same calculation, only in this case, $dia_{active}$ represents the vessel diameter at steady state following application of the given agent.

The majority of *in vitro* inhibitor studies utilized a pre/post experimental design: myogenic and phenylephrine responses were assessed following viability assessment; vessels were then incubated with 10 nmol/L - 1 μmol/L CKI-7 for 30 minutes (Cayman Chemical; distributed by Cedarlane Laboratories, Burlington, Canada); and myogenic and phenylephrine responses were reassessed in the presence of CKI-7. For **Figures 2E-F** in the main article, vessels were pre-treated with 10 nmol/L CKI-7 for 30 minutes following viability assessment, followed by response assessment in the presence of CKI-7.

For the *in vivo* inhibitor studies, wild-type mice were administered a single i.p. injection of PF670462 (30-50 mg/kg in 200 μl sterile water; Cayman Chemical) or vehicle (200 μl sterile water) between Zeitgeber time 10 (ZT10) and ZT10.5. Cremaster arteries were isolated the following day for functional assessment at ZT7.

***Western blotting.*** Cremaster arteries were isolated, cannulated and tested for viability; after a 30-minute incubation at 40 mmHg, arteries were subjected to either a 40-100 mmHg pressure step or no pressure step. After 5 minutes, vessels were snap-frozen in liquid nitrogen. Lysates were prepared by pooling and mechanically homogenizing 2 cremaster arteries in lysis buffer. Proteins were electrophoretically separated on a standard 9% SDS-PAGE gels and transferred to polyvinyl difluoride (PVDF) membranes. The PVDF membranes were fixed in 0.5% glutaraldehyde in PBS for 40 minutes and blocked with 5% milk in PBS for 1 hour. Blots were incubated with rabbit anti-phospho-p44/42 MAPK (ERK1/2) antibody (1:1000 in 2% bovine serum albumin; cat #4377S, Cell Signaling Technology Canada; Whitby Canada) or rabbit anti-p44/42 MAPK (ERK1/2) antibody (1:1000 in 2% bovine serum albumin; cat #4695S, Cell Signaling Technology) overnight at 4°C; conjugated with biotinylated donkey anti-rabbit IgG for 2 hours (1:20,000 in 2% bovine serum albumin; cat# AP182B; Millipore Sigma, Oakville Canada); and then high sensitivity peroxidase-conjugated streptavidin for 2 hours (1:500 in 2% bovine serum albumin; cat# 21130; ThermoFisher Scientific; Mississauga, Canada). Using standard chemiluminescence procedures, digital images were captured with a Bio-Rad Laboratories (Mississauga, Canada) Chemidoc Touch system and quantified using Bio-Rad Laboratories Image Lab software. Uncropped blots for all western blot experiments are displayed in **Supplemental Figures 15-17**.

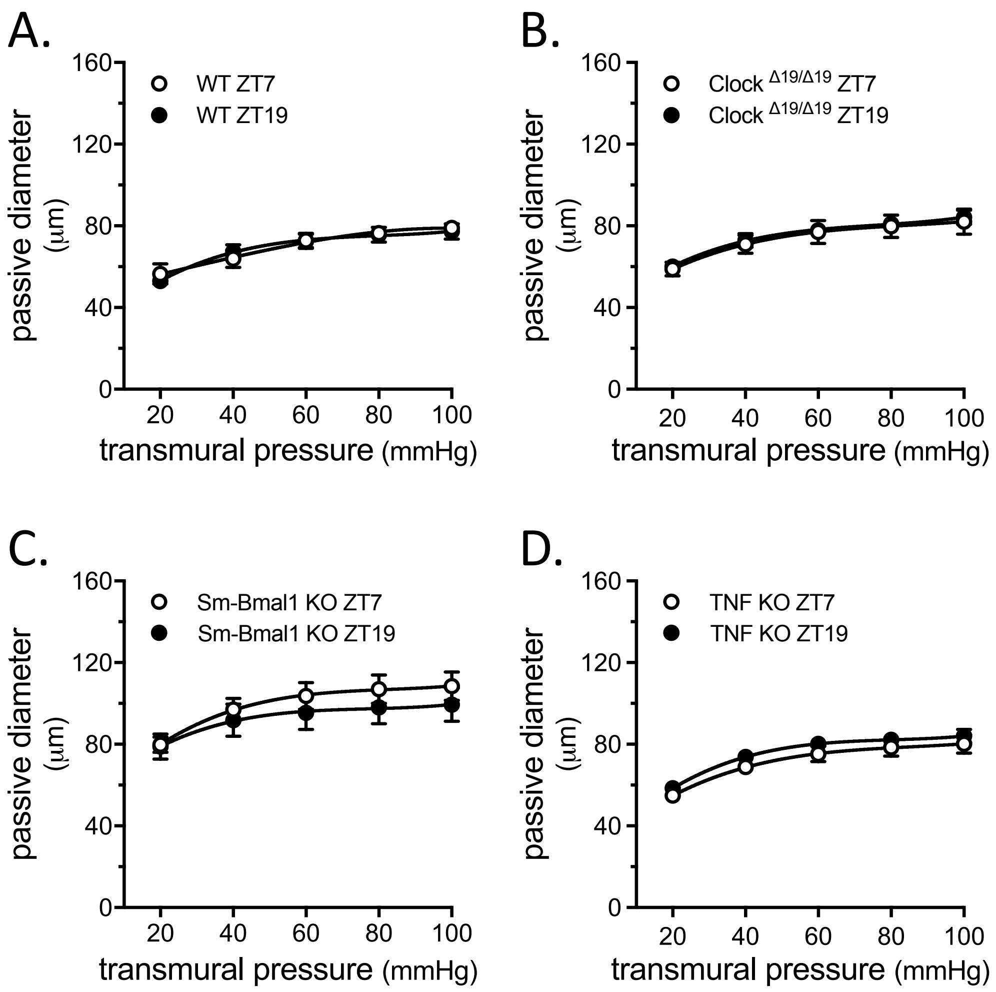


**Supplemental Figure 1: Passive diameter measurements for ZT7 / ZT19 comparisons.**

Passive diameter measurements for cremaster arteries isolated from (**A**) wild-type (WT; n=4-6) mice, **(B)** Clock$^{\Delta 19/\Delta 19}$ mutant mice (n=5-6), **(C)** smooth muscle cell-specific Bmal1 knockout mice (Sm-Bmal1 KO; n=5-6) and **(D)** tumor necrosis factor knockout mice (TNF KO; n=5) at Zeitgeber time 7 (ZT7) and Zeitgeber time 19 (ZT19). For all panels, P=N.S. for ZT7 versus ZT19 at a given transmural pressure.

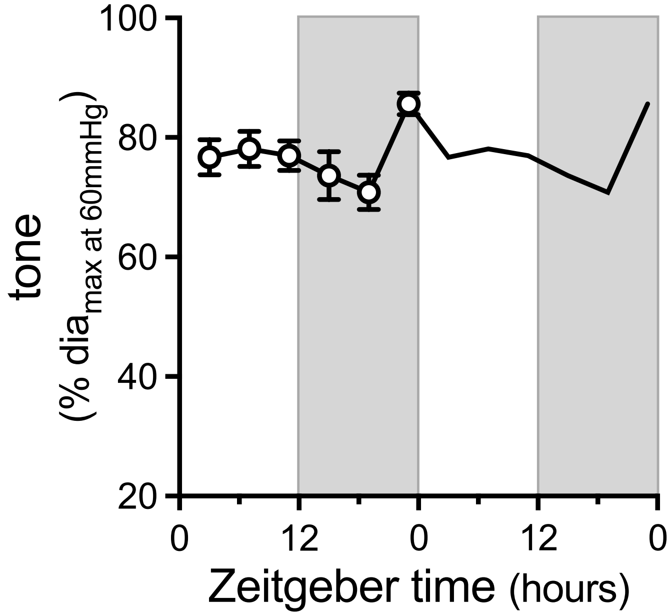


**Supplemental Figure 2: Depolarization-stimulated vasoconstriction in circadian time.**

Depolarization-induced vasoconstriction (60 mmol/L KCl) in wild-type cremaster arteries is plotted over Zeitgeber time (n=4-6); data are “double-plotted” for visualization purposes. P=N.S. for a circadian rhythm by JTK_CYCLE (see **Supplemental Table 6**).

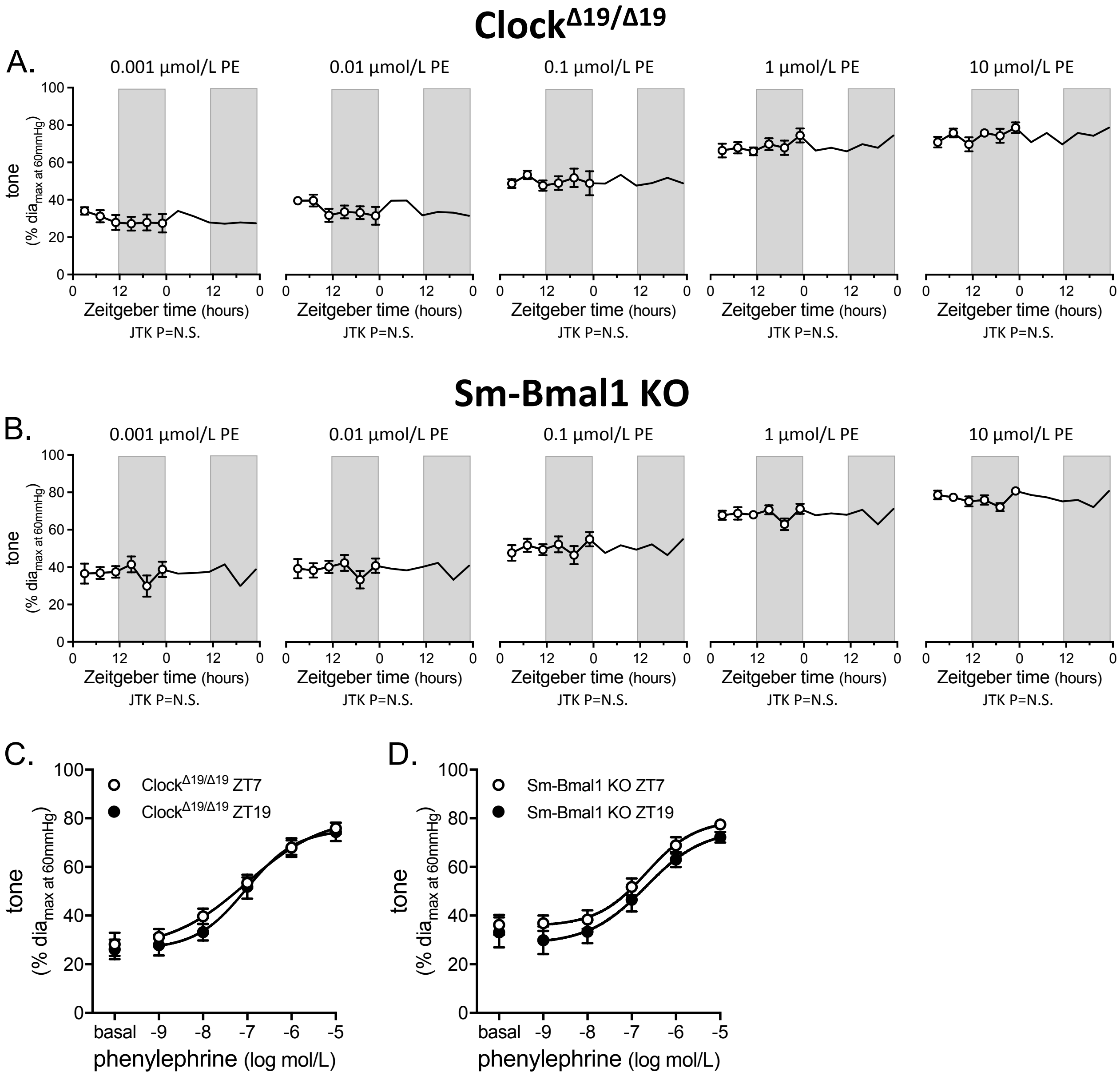


**Supplemental Figure 3: Cremaster artery phenylephrine responses in molecular clock disruption models**

Phenylephrine (PE)-stimulated vasoconstriction in cremaster arteries isolated from **(A)** Clock$^{\Delta19/\Delta19}$ mutant mice (n=5-7) and **(B)** smooth muscle cell-specific Bmal1 knockout mice (Sm-Bmal1 KO; n=5-6) plotted over Zeitgeber time; data are “double-plotted” for visualization purposes. In both mutant models, P=N.S. for a circadian rhythm at all phenylephrine concentrations by JTK_CYCLE (see **Supplemental Table 7**). In *Panels C-D*, phenylephrine responses at Zeitgeber time 7 (ZT7) and Zeitgeber time 19 (ZT19) are compared for **(C)** Clock$^{\Delta19/\Delta19}$ cremaster arteries (n=5-6) and **(D)** Sm-Bmal1 KO cremaster arteries (n=5-6). In both mutant models, P=N.S. for ZT7 versus ZT19 at all phenylephrine concentrations.

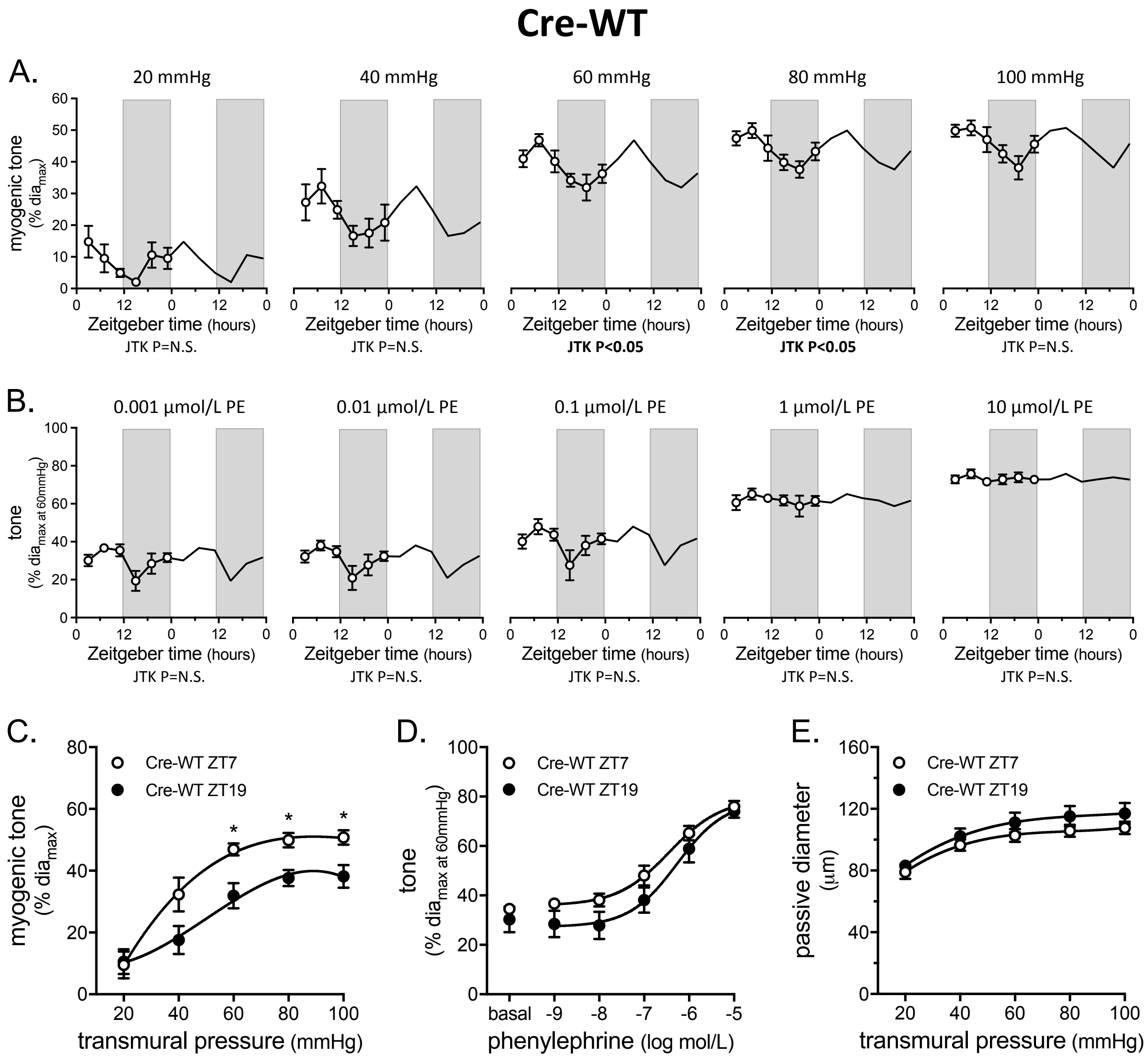


**Supplemental Figure 4: Control measurements in Cre-WT cremaster arteries**

**(A)** Myogenic- and **(B)** phenylephrine (PE)-stimulated vasoconstriction in cremaster arteries isolated from tamoxifen-treated, Cre-expressing wild-type control mice (Cre-WT) plotted over Zeitgeber time; data are "double-plotted" for visualization purposes (n=5-6). Myogenic vasoconstriction displays a statistically significant circadian rhythm (P<0.05 by JTK_CYCLE; see **Supplemental Table 7**), while phenylephrine responses do not (P=N.S. at all concentrations for a circadian rhythm by JTK_CYCLE; see **Supplemental Table 7**). **(C)** Myogenic vasoconstriction (n=6), **(D)** phenylephrine responses (n=6) and **(E)** passive diameters (n=6) are compared at Zeitgeber time 7 (ZT7) and Zeitgeber time 19 (ZT19). For *Panels C-E*, * denotes P<0.05 for ZT7 versus ZT19 at a given transmural pressure or phenylephrine concentration.

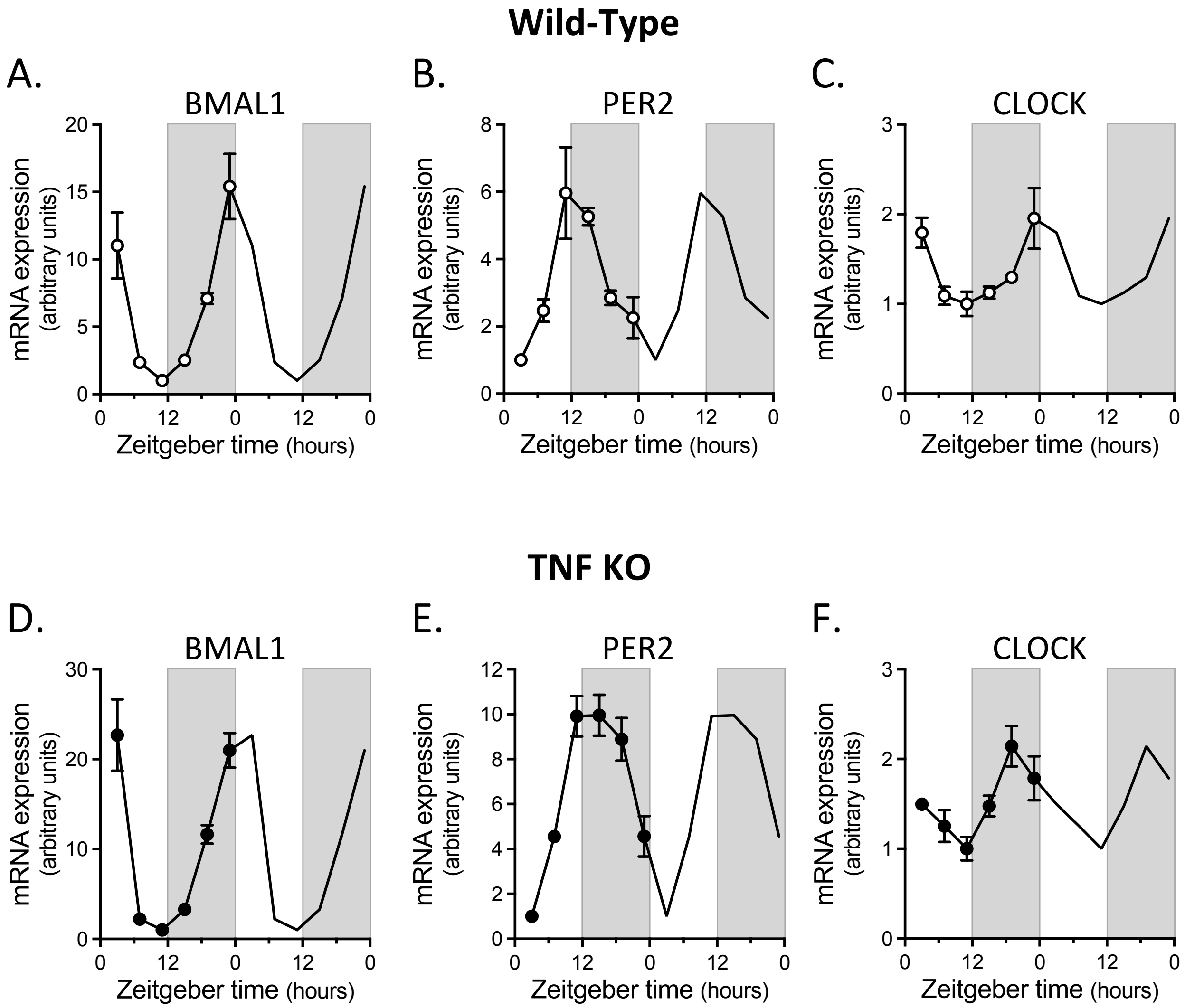


**Supplemental Figure 5: Circadian clock gene expression in wild-type and TNF KO mouse cremaster arteries.**

Wild-type cremaster artery **(A)** *Bmal1*, (**B**) *Per2* and (**C**) *Clock* mRNA expression oscillate with a circadian rhythm (n=3 for all panels). Likewise, tumor necrosis factor knockout (TNF KO) cremaster artery **(D)** *Bmal1*, (**E**) *Per2* and (**F**) *Clock* mRNA expression oscillate with a comparable circadian rhythm (n=3 for all panels). A statistical analysis for circadian rhythmicity is provided in **Supplemental Table 8**.

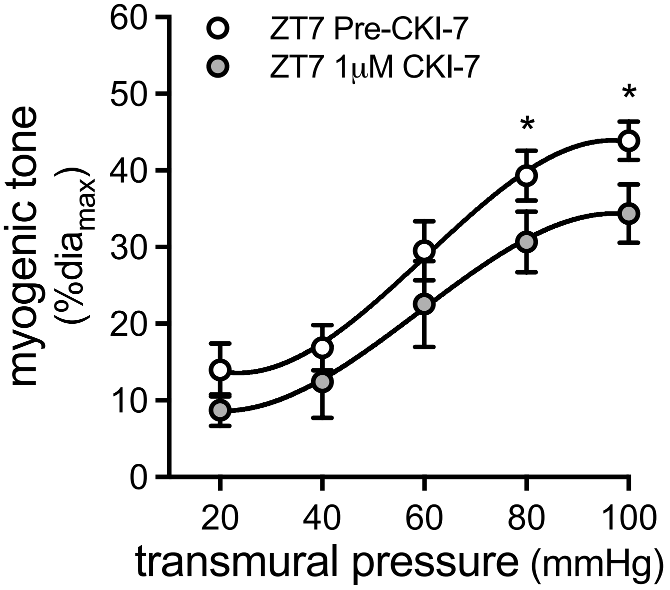


**Supplemental Figure 6: Myogenic tone following 1µmol/L CKI-7 treatment *in vitro*.**

Myogenic tone was assessed in cremaster arteries isolated from naïve wild-type mice at ZT7 (white circles). The arteries were then incubated with 1µmol/L CKI-7 for 30 minutes and myogenic tone was re-assessed (grey circles). * denotes $p<0.05$ (paired t-test comparisons).

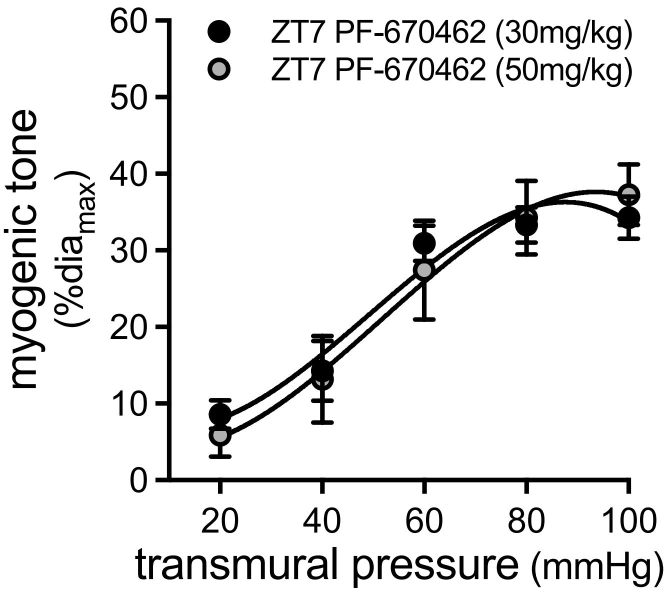


**Supplemental Figure 7: Myogenic tone following 50mg/kg PF670462.**

Naïve wild-type mice were treated with a single dose of PF670462 (50mg/kg i.p. within 2 hours of ZT7); cremaster arteries were harvested at ZT7 the following day. For comparison purposes, the 30mg/kg PF670462 is reproduced from **Figure 2I** of the main manuscript. Compared to the 30mg/kg dosage, 50mg/kg PF670462 has no appreciable effect.

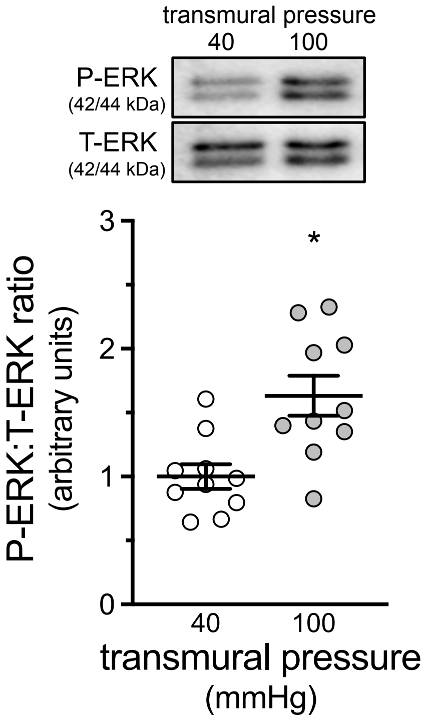


**Supplemental Figure 8: Pressure-stimulated ERK1/2 phosphorylation in cremaster arteries.**

Western blot assessment of ERK1/2 phosphorylation in naïve wild-type cremaster skeletal muscle resistance arteries maintained at 40 mmHg (n=10) or 5 minutes following a 40 mmHg to 100 mmHg pressure step (n=10). A representative western blot image is displayed above. A total of 10 independent experiments were performed, each with 1 vessel sample from each group. After pooling, the data were normalized to the mean of the 40 mmHg group. To account for differences in western blot conditions, the data are analyzed with a paired t-test. * denotes $p<0.05$.

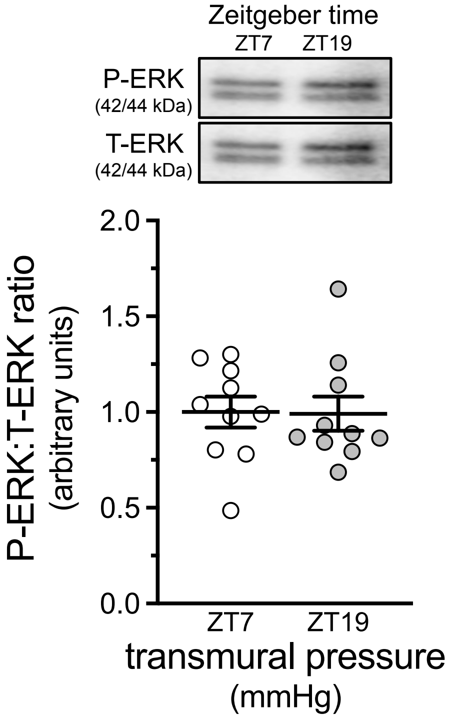


**Supplemental Figure 9: Pressure-stimulated ERK1/2 phosphorylation in cremaster arteries following casein kinase 1 inhibition.**

Western blot assessment of ERK1/2 phosphorylation in naïve wild-type cremaster skeletal muscle resistance arteries pre-treated with 10 µmol/L PF670462 (30 minutes) and then subjected to a pressure step from 40 mmHg to100 mmHg (lysates prepared 5 minutes post-pressure step; n=10). A representative western blot image is displayed above. A total of 10 independent experiments were performed, each with 1 vessel sample from each group. To account for differences in western blot conditions, the data are analyzed with paired statistical test (Wilcoxon test). P=N.S. for ZT7 versus ZT19.

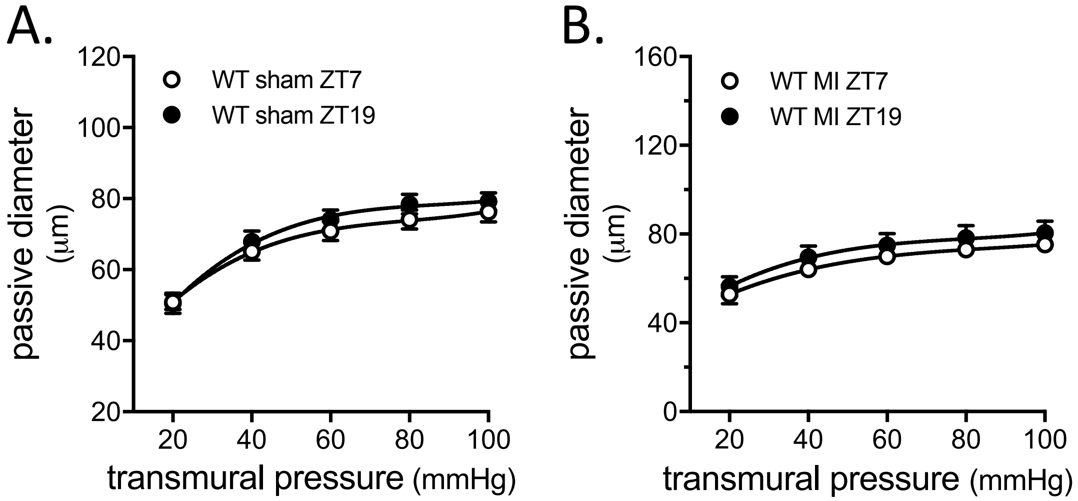


**Supplemental Figure 10: Passive diameter measurements for cremaster arteries from wild-type mice with myocardial infarction.**

*Panels A-B* display passive diameter measurements for wild-type (WT) cremaster arteries isolated at Zeitgeber time 7 (ZT7) and Zeitgeber time 19 (ZT19) from **(A)** sham-operated mice (n=6-9) and **(B)** mice with a myocardial infarction (MI; n=7). For both panels, P=N.S. for all ZT7 versus ZT19 comparisons at a given transmural pressure.

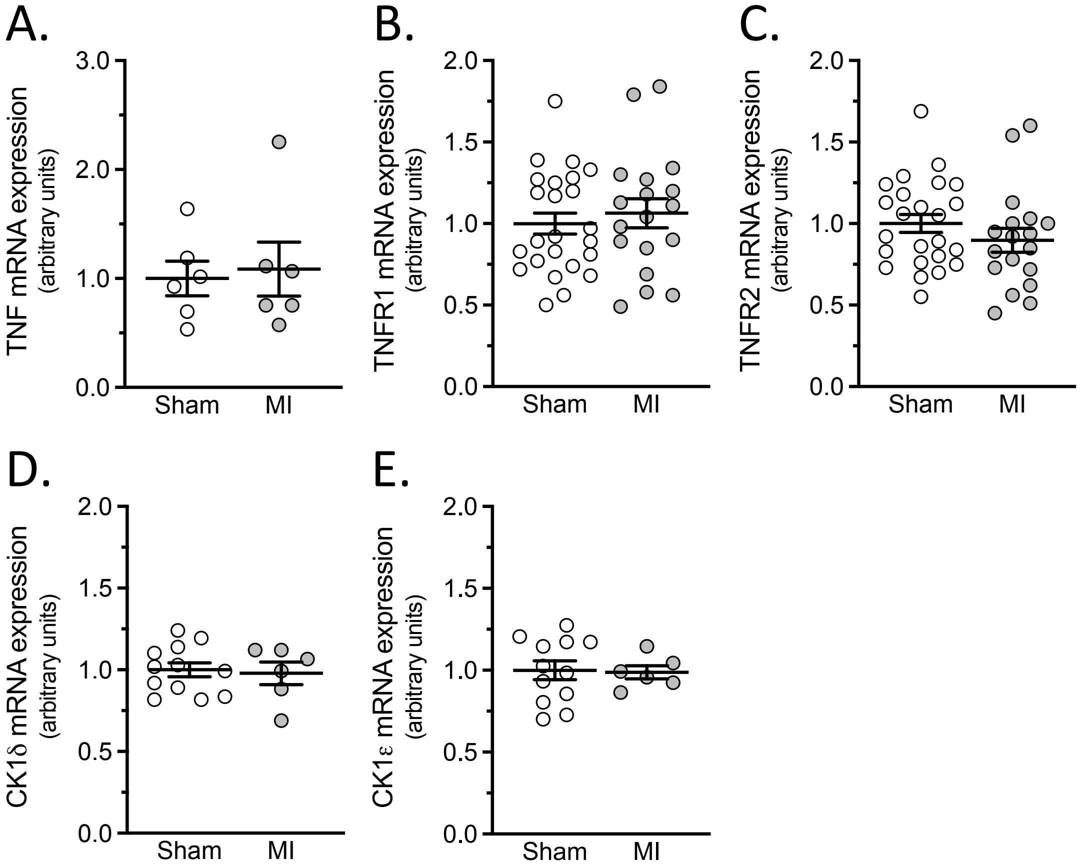


**Supplemental Figure 11: mTNF reverse signaling component mRNA expression in cremaster arteries isolated from wild-type mice with myocardial infarction.**

**(A)** Tumor necrosis factor (TNF; n=6), **(B)** tumor necrosis factor receptor 1 (TNFR1; n=24-18), **(C)** tumor necrosis factor receptor 2 (TNFR2; n=24-18), **(D)** Casein kinase 1 delta (CK1$\delta$; n=6-12) and **(E)** Casein kinase 1 epsilon (CK1$\varepsilon$; n=6-12) mRNA expression in cremaster arteries isolated from mice with a myocardial infarction (MI) and sham-operated controls. For all panels, P=N.S. for sham versus MI.

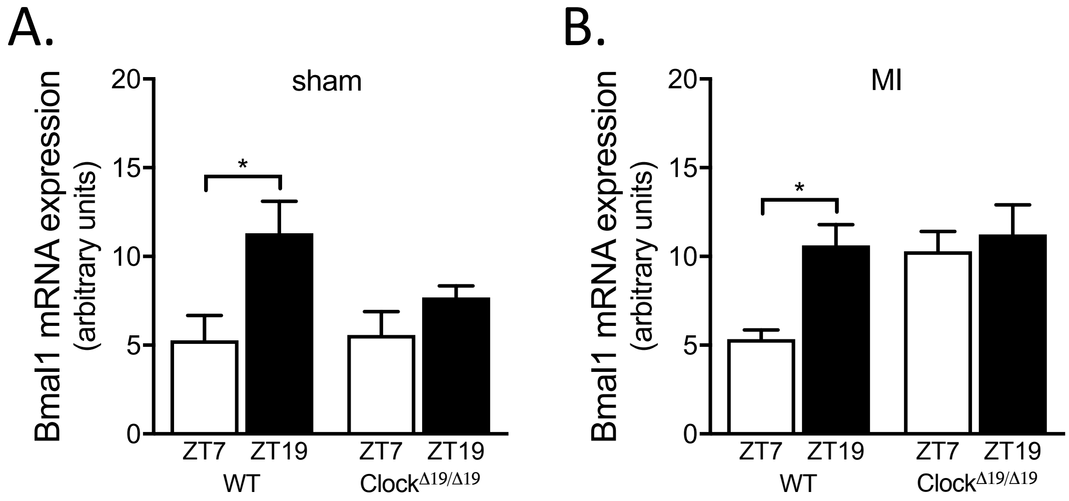


**Supplemental Figure 12: Bmal1 mRNA expression in cremaster arteries isolated from wild-type and ClockΔ19/Δ19 mice with myocardial infarction.**

**(A)** Bmal1 mRNA expression in cremaster arteries isolated at Zeitgeber time 7 (ZT7) and Zeitgeber time 19 (ZT19) from wild-type (WT) and Clock$^{\Delta19/\Delta19}$ mutant mice following **(A)** sham surgery (WT n=8; Clock$^{\Delta19/\Delta19}$ n=4-5) and **(B)** myocardial infarction (MI; WT n=7; Clock$^{\Delta19/\Delta19}$ n=3-4). * denotes $P<0.05$ for ZT7 versus ZT19 in the respective genotype/intervention groupings.

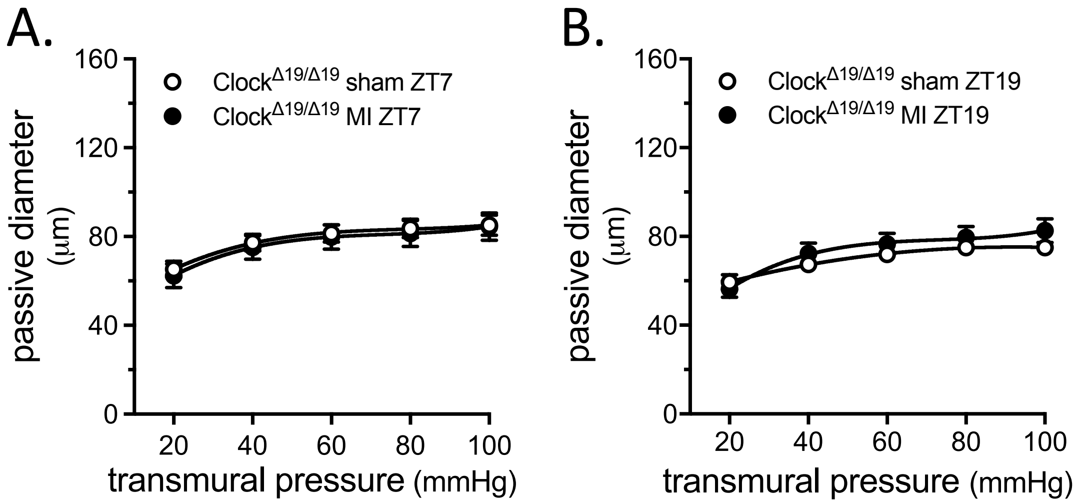


**Supplemental Figure 13: Passive diameter measurements for cremaster arteries from ClockΔ19/Δ19 mice with myocardial infarction.**

*Panels A-B* display passive diameter measurements for cremaster arteries isolated at **(A)** Zeitgeber time 7 (ZT7; n=7-8) and **(B)** Zeitgeber time 19 (ZT19; n=7-8) from Clock$^{\Delta19/\Delta19}$ mice following myocardial infarction (MI) or sham procedure. P=N.S. for all sham versus MI comparisons at a given transmural pressure.

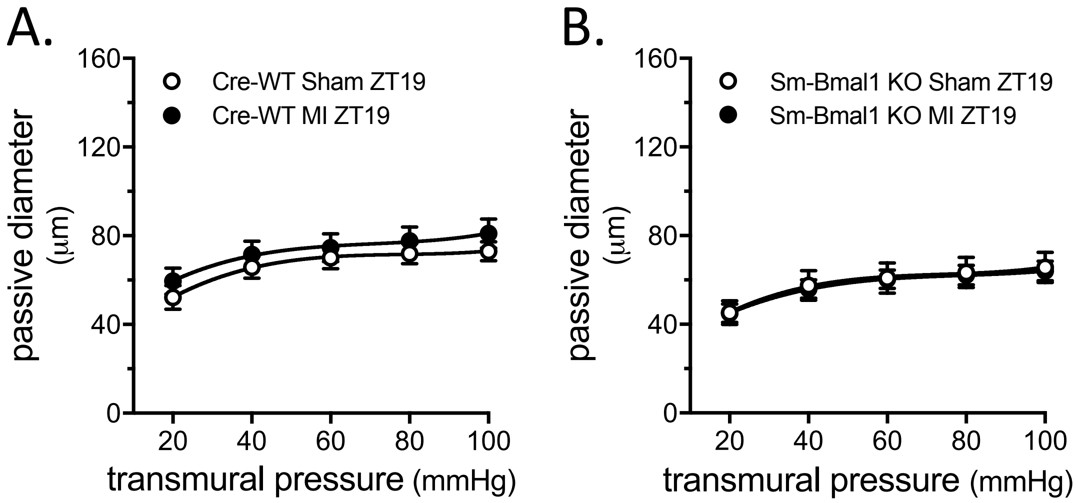


**Supplemental Figure 14: Passive diameter measurements for cremaster arteries from Cre-WT and Sm-Bmal1 KO mice with myocardial infarction.**

*Panels A-B* display passive diameter measurements for cremaster arteries isolated at Zeitgeber time 19 (ZT19) from **(A)** tamoxifen-treated, Cre-expressing wild-type control mice (Cre-WT) following myocardial infarction (MI) or sham procedure (n=7) and **(B)** smooth muscle cell-specific Bmal1 knockout (Sm-Bmal1 KO) mice following MI or sham procedure (n=7). P=N.S. for all sham versus MI comparisons at a given transmural pressure.

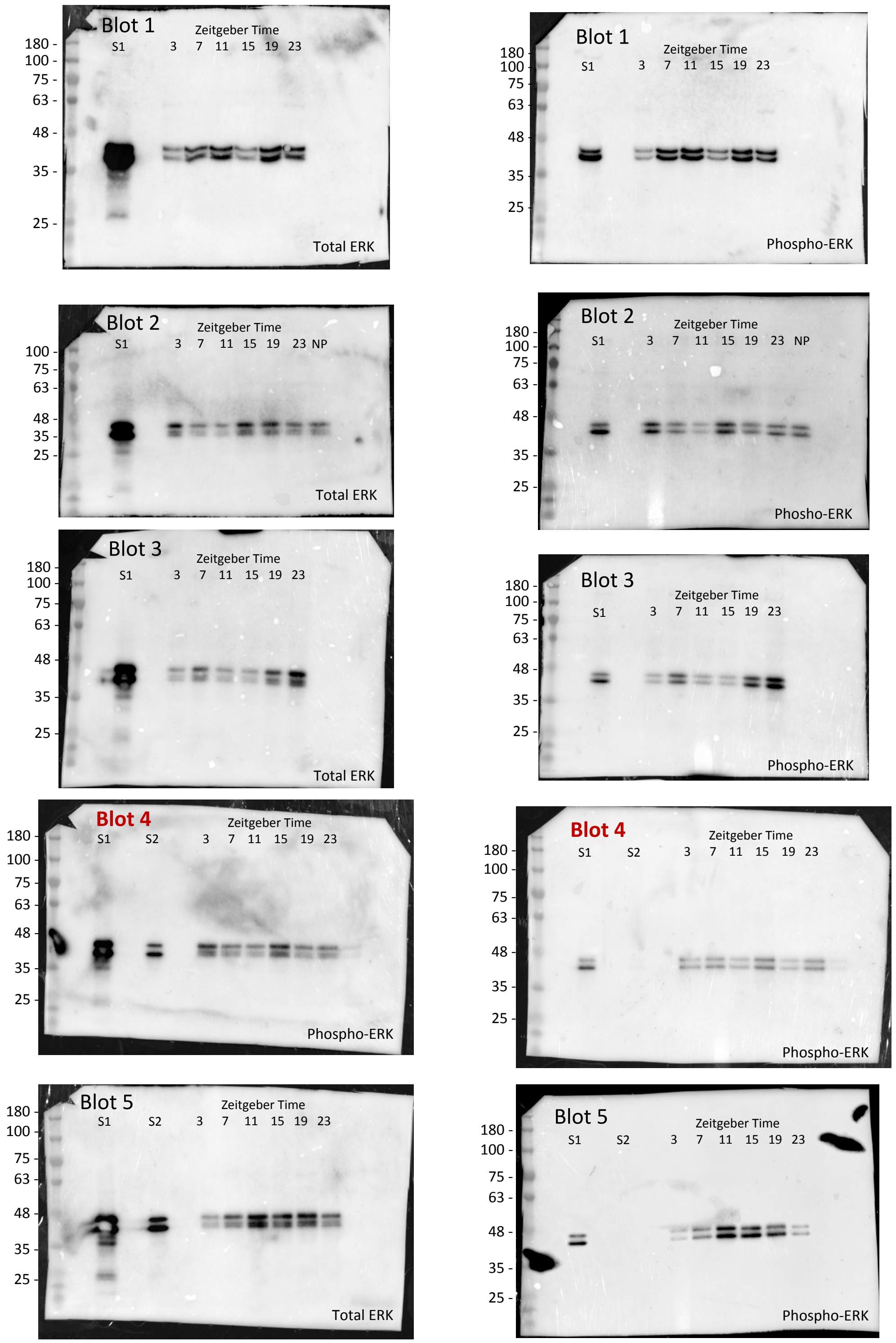


**Supplemental Figure 15: Uncropped western blot images – Circadian ERK phosphorylation.**

Shown are the uncropped, annotated western blots probing wild-type cremaster skeletal muscle artery lysates for total ERK1/2 (p42/p44) and phosphorylated ERK1/2. The artery lysates were prepared at Zeitgeber times 3, 7, 11, 15, 19 and 23 following a pressure step from 40 mmHg to 100 mmHg (5 minutes duration). All blots contain a standard (S1) to ensure similar detection; blots 4-5 possess a diluted standard (S2). Data from these blots are incorporated into Figures 2M and 2N; data from Blot 4 are displayed as a cropped image in **Figure 2M** (sample lanes 3-8; all data shown, excluding standards).

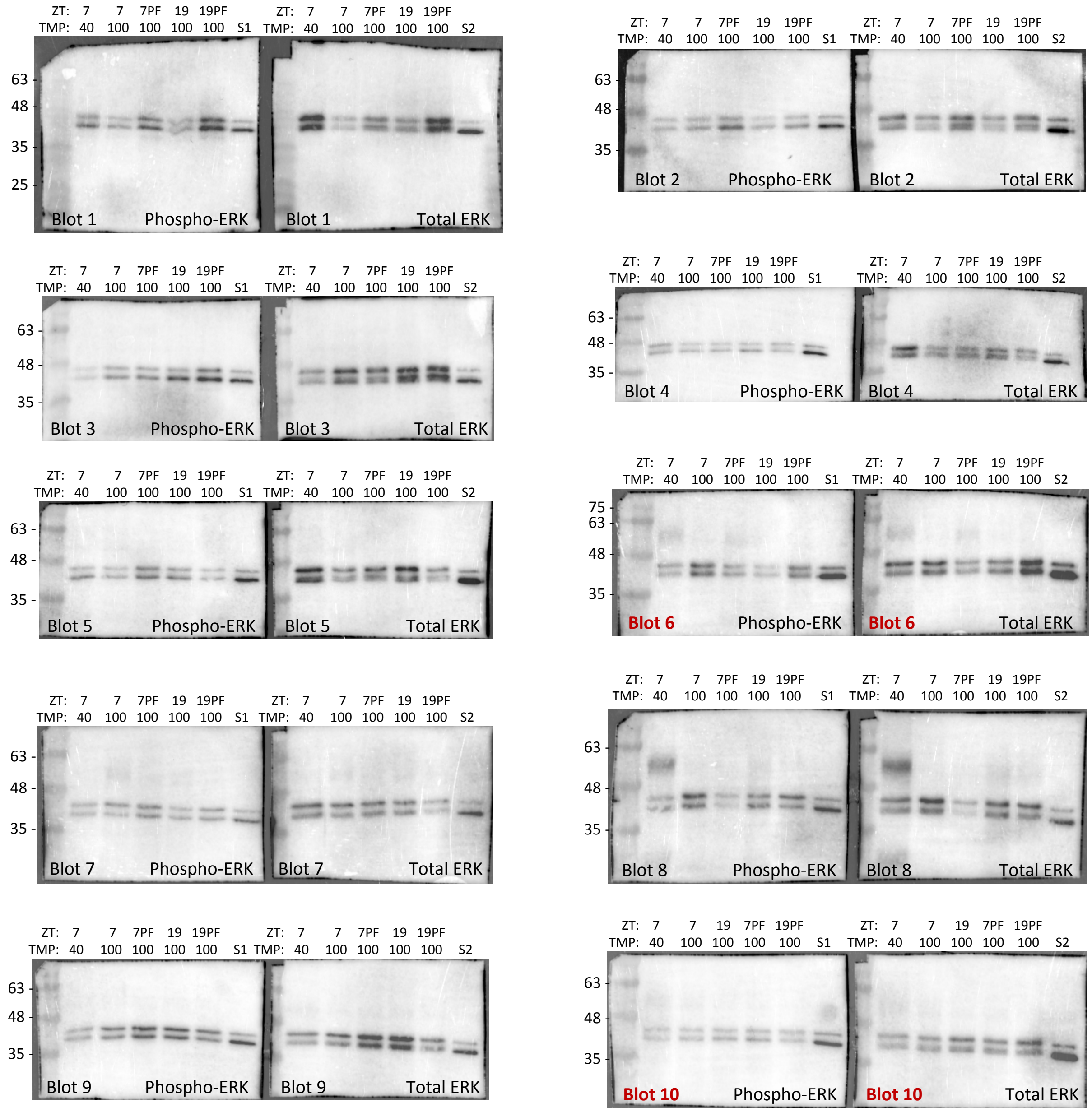


**Supplemental Figure 16: Uncropped western blot images – ERK phosphorylation (wild-type).**

Shown are the uncropped, annotated western blots probing wild-type cremaster skeletal muscle artery lysates for total ERK1/2 (p42/p44) and phosphorylated ERK1/2. The artery lysates were prepared at Zeitgeber times (ZT) 7 and 19, either without a pressure step (transmural pressure 40 mmHg; TMP: 40) or five minutes following a pressure step from 40 mmHg to 100 mmHg (TMP: 100). In certain experiments, arteries were pretreated with 10 µmol/L PF670462 (30 minutes) prior to the pressure step (denoted with a "PF" beside the Zeitgeber time). All blots contain a standard to ensure similar detection (S1 and S2; S2 is diluted in relation to S1). Data from these blots are incorporated into Figure 2N and Supplemental Figures 8-9; data from Blot 6 are displayed as a cropped image in **Figure 2N** (sample lanes 2 and 4) and **Supplemental Figure 8** (sample lanes 1 and 2); data from Blot 10 are displayed as a cropped image in **Supplemental Figure 9** (sample lanes 4-5).

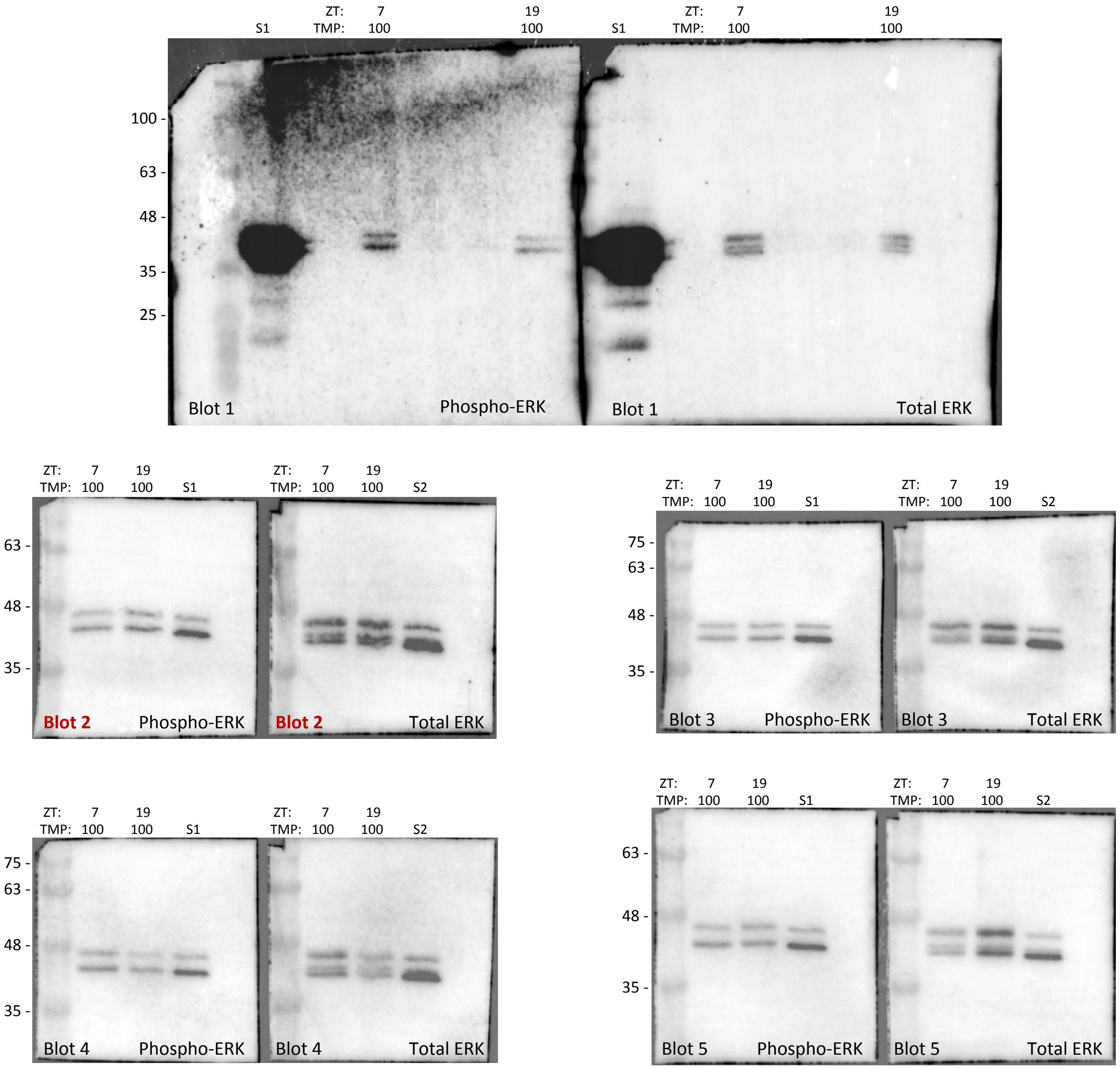


**Supplemental Figure 17: Uncropped western blot images – ERK phosphorylation (TNF KO).**

Shown are the uncropped, annotated western blots probing cremaster skeletal muscle artery lysates for total ERK1/2 (p42/p44) and phosphorylated ERK1/2. The artery lysates were prepared from tumor necrosis factor knockout mice (TNF KO) at Zeitgeber times (ZT) 7 and 19 five minutes following a pressure step from 40 mmHg to 100 mmHg (TMP: 100). All blots contain a standard to ensure similar detection (S1 and/or S2; S2 is diluted in relation to S1). Data from these blots are incorporated into Figure 2O; data from Blot 2 are displayed as a cropped image in **Figure 2O** (sample lanes 1 and 2).

**Supplemental Table 1: Quantitative PCR primer information**

| Gene | Primer Sequences (5' to 3') | Product Size (bp) | Efficiency | Accession no. |
|---|---|---|---|---|
| **Bmal1** | GCCACCAACCCATACACAGA<br>TCTTCCCTCGGTCACATCCT | 124 | 1.09 | NM_007489.4 |
| **Per2** | CACACTGCTGCCCTGAGTTC<br>ATCTGAGGACCAGCAGCACA | 120 | 0.98 | NM_011066.3 |
| **Clock** | TGCAGGTACCTTGCTCTGGA<br>GGTTTAACGCCAGCCTCAAG | 108 | 1.00 | NM_007715.6 |
| **Rev-Erbα** | GAAGTGTCTCTCCGTTGGCA<br>CTGCTCAGTTGGTTGTTGGC | 126 | 0.97 | NM_145434.4 |
| **GAPDH** | AGGTCGGTGTGAACGGATTTG<br>TGTAGACCATGTAGTTGAGGTCA | 123 | 0.94 | NM_008084.2 |
| **G6PD** | CACAGTGGACGACATCCGAAA<br>AGCTACATAGGAATTACGGGCAA | 103 | 1.02 | NM_008062 |
| **HMBS** | CCCGTAACATTCCAAGAGGA<br>CCTGTGCCCTACAGACCAGT | 147 | 1.08 | NM_013551.2 |

Abbreviations: Bmal1 = Brain and muscle aryl hydrocarbon receptor nuclear translocator-like protein 1, Per2 = Period circadian regulator 2, Clock = Circadian locomotor output cycles kaput, Rev-Erbα = Reverse strand of ERBA, GAPDH = glyceraldehyde 3-phosphate dehydrogenase, G6PD = glucose-6-phosphate dehydrogenase, HMBS = hydroxymethylbilane synthase.

## Supplemental Table 2: ZT7 / ZT19 statistical comparisons

| Figure | Genotype | Parameter | Level | ZT7 | n | N | ZT19 | n | N | Comparison Test | Statistical Outcome |
|---|---|---|---|---|---|---|---|---|---|---|---|
| 1B | Wild-Type | Myogenic | 20 mmHg | 37.0 ± 3.13 | 4 | 3 | 24.7 ± 6.49 | 6 | 3 | t-test | t(8) = 1.45, p = 0.186 |
| 1B | Wild-Type | Myogenic | 40 mmHg | 39.1 ± 4.26 | 4 | 3 | 34.0 ± 4.47 | 6 | 3 | t-test | t(8) = 0.794, p = 0.450 |
| 1B | Wild-Type | Myogenic | 60 mmHg | 48.0 ± 2.62 | 4 | 3 | 35.6 ± 4.19 | 6 | 3 | t-test | t(8) = 2.19, p = 0.060 |
| 1B | Wild-Type | Myogenic | 80 mmHg | 53.8 ± 2.09 | 4 | 3 | 39.7 ± 2.52 | 6 | 3 | Mann-Whitney | U($N_{ZT7}$ = 4, $N_{ZT19}$ = 6) = 0, **p = 0.010** |
| 1B | Wild-Type | Myogenic | 100 mmHg | 54.4 ± 2.77 | 4 | 3 | 40.7 ± 3.08 | 6 | 3 | t-test | t(8) = 3.10, **p = 0.015** |
| 1D | Wild-Type | Phenylephrine | Basal | 35.8 ± 3.11 | 4 | 3 | 24.6 ± 5.14 | 6 | 3 | t-test | t(8) = 1.64, p = 0.140 |
| 1D | Wild-Type | Phenylephrine | 1 nmol/L | 42.5 ± 3.42 | 4 | 3 | 27.8 ± 4.62 | 6 | 3 | t-test | t(8) = 2.431, **p = 0.049** |
| 1D | Wild-Type | Phenylephrine | 10 nmol/L | 50.8 ± 2.64 | 4 | 3 | 35.1 ± 5.33 | 6 | 3 | Mann-Whitney | U($N_{ZT7}$ = 4, $N_{ZT19}$ = 6) = 6, p = 0.257 |
| 1D | Wild-Type | Phenylephrine | 100 nmol/L | 62.9 ± 2.17 | 4 | 3 | 50.6 ± 6.47 | 6 | 3 | t-test | t(8) = 1.49, p = 0.174 |
| 1D | Wild-Type | Phenylephrine | 1 µmol/L | 72.7 ± 2.28 | 4 | 3 | 67.9 ± 4.45 | 6 | 3 | t-test | t(8) = 0.83, p = 0.430 |
| 1D | Wild-Type | Phenylephrine | 10 µmol/L | 75.1 ± 3.25 | 4 | 3 | 76.0 ± 3.47 | 6 | 3 | t-test | t(8) = 0.19, p = 0.853 |
| 1F | Clock$^{\Delta19/\Delta19}$ | Myogenic | 20 mmHg | 14.9 ± 3.8 | 6 | 3 | 16.5 ± 5.5 | 5 | 3 | t-test | t(9) = 0.25, p = 0.811 |
| 1F | Clock$^{\Delta19/\Delta19}$ | Myogenic | 40 mmHg | 27.9 ± 3.4 | 6 | 3 | 26.1 ± 6.1 | 5 | 3 | t-test | t(9) = 0.27, p = 0.793 |
| 1F | Clock$^{\Delta19/\Delta19}$ | Myogenic | 60 mmHg | 39.8 ± 4.3 | 6 | 3 | 35.0 ± 4.3 | 5 | 3 | t-test | t(9) = 0.78, p = 0.456 |
| 1F | Clock$^{\Delta19/\Delta19}$ | Myogenic | 80 mmHg | 44.1 ± 2.6 | 6 | 3 | 37.1 ± 3.8 | 5 | 3 | t-test | t(9) = 1.55, p = 0.156 |
| 1F | Clock$^{\Delta19/\Delta19}$ | Myogenic | 100 mmHg | 45.4 ± 2.0 | 6 | 3 | 39.3 ± 6.0 | 5 | 3 | t-test | t(9) = 1.03, p = 0.330 |
| 1H | Sm-Bmal1 KO | Myogenic | 20 mmHg | 15.4 ± 4.5 | 6 | 3 | 13.3 ± 6.5 | 5 | 3 | Mann-Whitney | U($N_{ZT7}$ = 6, $N_{ZT19}$ = 5) = 13, p = 0.792 |
| 1H | Sm-Bmal1 KO | Myogenic | 40 mmHg | 32.0 ± 3.7 | 6 | 3 | 29.5 ± 5.7 | 5 | 3 | t-test | t(9) = 0.38, p = 0.709 |
| 1H | Sm-Bmal1 KO | Myogenic | 60 mmHg | 43.5 ± 3.3 | 6 | 3 | 43.0 ± 4.6 | 5 | 3 | Mann-Whitney | U($N_{ZT7}$ = 6, $N_{ZT19}$ = 5) = 15, p = 1.000 |
| 1H | Sm-Bmal1 KO | Myogenic | 80 mmHg | 48.3 ± 3.3 | 6 | 3 | 48.2 ± 4.7 | 5 | 3 | t-test | t(9) = 0.02, p = 0.980 |
| 1H | Sm-Bmal1 KO | Myogenic | 100 mmHg | 50.4 ± 3.3 | 6 | 3 | 50.3 ± 4.6 | 5 | 3 | t-test | t(9) = 0.02, p = 0.985 |
| 2B | TNF KO | Myogenic | 20 mmHg | 8.1 ± 2.7 | 5 | 3 | 11.3 ± 3.0 | 5 | 3 | t-test | t(8) = 0.78, p = 0.459 |
| 2B | TNF KO | Myogenic | 40 mmHg | 11.4 ± 3.1 | 5 | 3 | 18.5 ± 4.3 | 5 | 3 | t-test | t(8) = 1.34, p = 0.216 |
| 2B | TNF KO | Myogenic | 60 mmHg | 21.6 ± 3.9 | 5 | 3 | 28.8 ± 2.7 | 5 | 3 | t-test | t(8) = 1.51, p = 0.169 |
| 2B | TNF KO | Myogenic | 80 mmHg | 26.1 ± 2.3 | 5 | 3 | 36.0 ± 1.0 | 5 | 3 | t-test | t(8) = 3.95, **p = 0.004** |
| 2B | TNF KO | Myogenic | 100 mmHg | 30.2 ± 2.6 | 5 | 3 | 40.4 ± 0.9 | 5 | 3 | t-test | t(8) = 3.75, **p = 0.006** |
| 2D | TNF KO | Phenylephrine | 1 nmol/L | 19.5 ± 3.0 | 5 | 3 | 25.0 ± 4.5 | 5 | 3 | t-test | t(8) = 1.04, p = 0.323 |
| 2D | TNF KO | Phenylephrine | 10 nmol/L | 19.8 ± 3.4 | 5 | 3 | 25.6 ± 4.5 | 5 | 3 | t-test | t(8) = 1.03, p = 0.335 |
| 2D | TNF KO | Phenylephrine | 100 nmol/L | 22.0 ± 4.2 | 5 | 3 | 29.0 ± 4.2 | 5 | 3 | t-test | t(8) = 1.19, p = 0.268 |
| 2D | TNF KO | Phenylephrine | 1 µmol/L | 37.8 ± 3.1 | 5 | 3 | 48.2 ± 1.6 | 5 | 3 | t-test | t(8) = 2.95, **p = 0.019** |
| 2D | TNF KO | Phenylephrine | 10 µmol/L | 70.3 ± 4.0 | 5 | 3 | 72.4 ± 3.1 | 5 | 3 | Mann-Whitney | U($N_{ZT7}$ = 5, $N_{ZT19}$ = 5) = 11, p = 0.841 |
| 2N | Wild-Type | ERK Phosphorylation | -- | 1.00 ± 0.08 | 15 | 19 | 0.78 ± 0.06 | 15 | 19 | paired t-test | t(14) = 3.47, **p = 0.004** |
| 2O | TNF KO | ERK Phosphorylation | -- | 1.00 ± 0.11 | 5 | 6 | 0.90 ± 0.10 | 5 | 6 | paired t-test | t(4) = 0.78, p = 0.480 |
| 3A | Wild-Type | Sham Myogenic | 20 mmHg | 13.0 ± 3.2 | 9 | 5 | 9.3 ± 3.2 | 6 | 3 | t-test | t(13) = 0.78, p = 0.450 |
| 3A | Wild-Type | Sham Myogenic | 40 mmHg | 25.0 ± 4.2 | 9 | 5 | 22.0 ± 2.0 | 6 | 3 | t-test | t(13) = 0.57, p = 0.580 |
| 3A | Wild-Type | Sham Myogenic | 60 mmHg | 37.4 ± 2.2 | 9 | 5 | 29.8 ± 2.4 | 6 | 3 | t-test | t(13) = 2.30, **p = 0.039** |
| 3A | Wild-Type | Sham Myogenic | 80 mmHg | 42.7 ± 1.8 | 9 | 5 | 34.5 ± 3.6 | 6 | 3 | t-test | t(13) = 2.26, **p = 0.042** |
| 3A | Wild-Type | Sham Myogenic | 100 mmHg | 44.4 ± 2.2 | 9 | 5 | 34.8 ± 4.5 | 6 | 3 | t-test | t(13) = 2.13, p = 0.053 |
| 3A | Wild-Type | MI Myogenic | 20 mmHg | 24.4 ± 5.7 | 7 | 4 | 23.9 ± 3.7 | 7 | 4 | t-test | t(12) = 0.07, p = 0.949 |
| 3A | Wild-Type | MI Myogenic | 40 mmHg | 32.0 ± 5.9 | 7 | 4 | 35.9 ± 3.3 | 7 | 4 | t-test | t(12) = 0.58, p = 0.570 |
| 3A | Wild-Type | MI Myogenic | 60 mmHg | 40.5 ± 3.0 | 7 | 4 | 42.5 ± 2.2 | 7 | 4 | t-test | t(12) = 0.53, p = 0.600 |
| 3A | Wild-Type | MI Myogenic | 80 mmHg | 46.2 ± 2.2 | 7 | 4 | 46.7 ± 2.0 | 7 | 4 | t-test | t(12) = 0.18, p = 0.864 |
| 3A | Wild-Type | MI Myogenic | 100 mmHg | 46.9 ± 1.9 | 7 | 4 | 48.0 ± 1.9 | 7 | 4 | t-test | t(12) = 0.42, p = 0.686 |
| 3B | Wild-Type | Sham Phenylephrine | Basal | 19.7 ± 2.9 | 9 | 5 | 16.7 ± 4.4 | 6 | 3 | t-test | t(13) = 0.60, p = 0.561 |
| 3B | Wild-Type | Sham Phenylephrine | 1 nmol/L | 23.0 ± 3.1 | 9 | 5 | 18.4 ± 3.6 | 6 | 3 | t-test | t(13) = 0.97, p = 0.352 |
| 3B | Wild-Type | Sham Phenylephrine | 10 nmol/L | 24.6 ± 3.7 | 9 | 5 | 19.8 ± 3.8 | 6 | 3 | t-test | t(13) = 0.87, p = 0.400 |
| 3B | Wild-Type | Sham Phenylephrine | 100 nmol/L | 37.8 ± 2.0 | 9 | 5 | 27.6 ± 4.0 | 6 | 3 | Mann-Whitney | U($N_{ZT7}$ = 9, $N_{ZT19}$ = 6) = 10, **p = 0.049** |
| 3B | Wild-Type | Sham Phenylephrine | 1 µmol/L | 59.1 ± 1.7 | 9 | 5 | 49.5 ± 6.3 | 6 | 3 | Mann-Whitney | U($N_{ZT7}$ = 9, $N_{ZT19}$ = 6) = 17.5, p = 0.285 |
| 3B | Wild-Type | Sham Phenylephrine | 10 µmol/L | 72.1 ± 2.0 | 9 | 5 | 70.4 ± 2.3 | 6 | 3 | t-test | t(13) = 0.57, p = 0.577 |
| 3C | Wild-Type | MI Phenylephrine | Basal | 27.7 ± 3.9 | 7 | 4 | 23.1 ± 4.8 | 7 | 4 | t-test | t(12) = 0.74, p = 0.473 |
| 3C | Wild-Type | MI Phenylephrine | 1 nmol/L | 30.9 ± 3.6 | 7 | 4 | 25.5 5.2 | 7 | 4 | t-test | t(12) = 0.85, p = 0.411 |
| 3C | Wild-Type | MI Phenylephrine | 10 nmol/L | 30.0 ± 2.3 | 7 | 4 | 28.5 4.8 | 7 | 4 | t-test | t(12) = 0.27, p = 0.791 |
| 3C | Wild-Type | MI Phenylephrine | 100 nmol/L | 37.0 ± 3.0 | 7 | 4 | 41.1 3.8 | 7 | 4 | t-test | t(12) = 0.84, p = 0.419 |
| 3C | Wild-Type | MI Phenylephrine | 1 µmol/L | 59.2 ± 1.8 | 7 | 4 | 64.4 2.3 | 7 | 4 | t-test | t(12) = 1.76, p = 0.104 |
| 3C | Wild-Type | MI Phenylephrine | 10 µmol/L | 72.4 ± 2.3 | 7 | 4 | 76.3 1.1 | 7 | 4 | t-test | t(12) = 1.54, p = 0.150 |
| Sup 1A | Wild-Type | Passive Diameter | 20 mmHg | 56.5 ± 4.9 | 4 | 3 | 53.0 ± 3.1 | 6 | 3 | t-test | t(8) = 0.64, p = 0.541 |
| Sup 1A | Wild-Type | Passive Diameter | 40 mmHg | 63.9 ± 4.2 | 4 | 3 | 67.4 ± 3.3 | 6 | 3 | Mann-Whitney | U($N_{ZT7}$ = 4, $N_{ZT19}$ = 6) = 7, p = 0.352 |
| Sup 1A | Wild-Type | Passive Diameter | 60 mmHg | 72.9 ± 3.5 | 4 | 3 | 72.5 ± 3.5 | 6 | 3 | t-test | t(8) = 0.08, p = 0.941 |
| Sup 1A | Wild-Type | Passive Diameter | 80 mmHg | 76.5 ± 2.3 | 4 | 3 | 75.6 ± 3.7 | 6 | 3 | t-test | t(8) = 0.17, p = 0.869 |
| Sup 1A | Wild-Type | Passive Diameter | 100 mmHg | 79.1 ± 2.9 | 4 | 3 | 77.3 ± 3.7 | 6 | 3 | t-test | t(8) = 0.35, p = 0.739 |

*...Supplemental Table 2 continues on the next page*

| Figure | Genotype | Parameter | Level | ZT7 | n | N | ZT19 | n | N | Comparison Test | Statistical Outcome |
|---|---|---|---|---|---|---|---|---|---|---|---|
| Sup 1B | $Clock^{\Delta19/\Delta19}$ | Passive Diameter | 20 mmHg | 58.8 ± 3.5 | 6 | 3 | 60.1 ± 3.0 | 5 | 3 | Mann-Whitney | $U(N_{ZT7} = 6, N_{ZT19} = 5) = 15, p = 1.000$ |
| Sup 1B | $Clock^{\Delta19/\Delta19}$ | Passive Diameter | 40 mmHg | 71.0 ± 4.4 | 6 | 3 | 72.8 ± 3.3 | 5 | 3 | t-test | $t(9) = 0.32, p = 0.753$ |
| Sup 1B | $Clock^{\Delta19/\Delta19}$ | Passive Diameter | 60 mmHg | 77.1 ± 5.7 | 6 | 3 | 77.9 ± 2.4 | 5 | 3 | t-test | $t(8) = 0.12, p = 0.905$ |
| Sup 1B | $Clock^{\Delta19/\Delta19}$ | Passive Diameter | 80 mmHg | 79.8 ± 5.6 | 6 | 3 | 81.1 ± 2.3 | 5 | 3 | t-test | $t(8) = 0.19, p = 0.854$ |
| Sup 1B | $Clock^{\Delta19/\Delta19}$ | Passive Diameter | 100 mmHg | 82.1 ± 6.1 | 6 | 3 | 84.2 ± 3.5 | 5 | 3 | t-test | $t(8) = 0.28, p = 0.783$ |
| Sup 1C | Sm-Bmal1 KO | Passive Diameter | 20 mmHg | 79.8 ± 3.8 | 6 | 3 | 78.8 ± 6.2 | 5 | 3 | t-test | $t(9) = 0.15, p = 0.886$ |
| Sup 1C | Sm-Bmal1 KO | Passive Diameter | 40 mmHg | 97.0 ± 5.4 | 6 | 3 | 91.8 ± 7.9 | 5 | 3 | Mann-Whitney | $U(N_{ZT7} = 6, N_{ZT19} = 5) = 12, p = 0.628$ |
| Sup 1C | Sm-Bmal1 KO | Passive Diameter | 60 mmHg | 103.7 ± 6.5 | 6 | 3 | 95.4 ± 8.2 | 5 | 3 | t-test | $t(9) = 0.80, p = 0.442$ |
| Sup 1C | Sm-Bmal1 KO | Passive Diameter | 80 mmHg | 107.0 ± 6.9 | 6 | 3 | 98.0 ± 8.1 | 5 | 3 | t-test | $t(9) = 0.85, p = 0.416$ |
| Sup 1C | Sm-Bmal1 KO | Passive Diameter | 100 mmHg | 108.5 ± 6.9 | 6 | 3 | 99.4 ± 8.2 | 5 | 3 | t-test | $t(9) = 0.86, p = 0.414$ |
| Sup 1D | TNF KO | Passive Diameter | 20 mmHg | 54.8 ± 1.6 | 5 | 3 | 58.4 ± 2.7 | 5 | 3 | t-test | $t(8) = 1.14, p = 0.286$ |
| Sup 1D | TNF KO | Passive Diameter | 40 mmHg | 68.8 ± 3.1 | 5 | 3 | 73.8 ± 2.1 | 5 | 3 | t-test | $t(8) = 1.35, p = 0.213$ |
| Sup 1D | TNF KO | Passive Diameter | 60 mmHg | 75.2 ± 3.9 | 5 | 3 | 80.2 ± 2.8 | 5 | 3 | t-test | $t(8) = 1.05, p = 0.323$ |
| Sup 1D | TNF KO | Passive Diameter | 80 mmHg | 78.4 ± 4.3 | 5 | 3 | 82.2 ± 3.0 | 5 | 3 | t-test | $t(8) = 0.73, p = 0.486$ |
| Sup 1D | TNF KO | Passive Diameter | 100 mmHg | 80.2 ± 4.5 | 5 | 3 | 84.0 ± 3.2 | 5 | 3 | t-test | $t(8) = 0.68, p = 0.513$ |
| Sup 3C | Sm-Bmal1 KO | Phenylephrine | Basal | 36.2 ± 4.1 | 6 | 3 | 33.1 ± 6.2 | 5 | 3 | Mann-Whitney | $U(N_{ZT7} = 6, N_{ZT19} = 5) = 14, p = 0.931$ |
| Sup 3C | Sm-Bmal1 KO | Phenylephrine | 1 nmol/L | 36.9 ± 3.2 | 6 | 3 | 29.9 ± 5.7 | 5 | 3 | t-test | $t(9) = 1.13, p = 0.289$ |
| Sup 3C | Sm-Bmal1 KO | Phenylephrine | 10 nmol/L | 38.4 ± 3.8 | 6 | 3 | 33.3 ± 4.7 | 5 | 3 | t-test | $t(9) = 0.84, p = 0.424$ |
| Sup 3C | Sm-Bmal1 KO | Phenylephrine | 100 nmol/L | 51.8 ± 3.5 | 6 | 3 | 46.6 ± 4.9 | 5 | 3 | Mann-Whitney | $U(N_{ZT7} = 6, N_{ZT19} = 5) = 11, p = 0.537$ |
| Sup 3C | Sm-Bmal1 KO | Phenylephrine | 1 µmol/L | 68.9 ± 3.3 | 6 | 3 | 63.0 ± 3.1 | 5 | 3 | Mann-Whitney | $U(N_{ZT7} = 6, N_{ZT19} = 5) = 6, p = 0.126$ |
| Sup 3C | Sm-Bmal1 KO | Phenylephrine | 10 µmol/L | 77.5 ± 1.7 | 6 | 3 | 72.2 ± 2.2 | 5 | 3 | t-test | $t(9) = 1.93, p = 0.085$ |
| Sup 3D | $Clock^{\Delta19/\Delta19}$ | Phenylephrine | Basal | 28.2 ± 4.7 | 6 | 3 | 26.1 ± 4.0 | 5 | 3 | t-test | $t(9) = 0.33, p = 0.748$ |
| Sup 3D | $Clock^{\Delta19/\Delta19}$ | Phenylephrine | 1 nmol/L | 31.2 ± 3.3 | 6 | 3 | 27.9 ± 4.3 | 5 | 3 | Mann-Whitney | $U(N_{ZT7} = 6, N_{ZT19} = 5) = 14, p = 0.931$ |
| Sup 3D | $Clock^{\Delta19/\Delta19}$ | Phenylephrine | 10 nmol/L | 39.7 ± 3.2 | 6 | 3 | 33.2 ± 3.4 | 5 | 3 | t-test | $t(9) = 1.39, p = 0.199$ |
| Sup 3D | $Clock^{\Delta19/\Delta19}$ | Phenylephrine | 100 nmol/L | 53.5 ± 2.3 | 6 | 3 | 51.8 ± 4.9 | 5 | 3 | t-test | $t(9) = 0.32, p = 0.757$ |
| Sup 3D | $Clock^{\Delta19/\Delta19}$ | Phenylephrine | 1 µmol/L | 68.0 ± 3.0 | 6 | 3 | 68.0 ± 3.8 | 5 | 3 | t-test | $t(9) = 0.001, p = 0.999$ |
| Sup 3D | $Clock^{\Delta19/\Delta19}$ | Phenylephrine | 10 µmol/L | 75.8 ± 2.4 | 6 | 3 | 74.4 ± 3.8 | 5 | 3 | t-test | $t(9) = 0.34, p = 0.740$ |
| Sup 4C | Cre-WT | Myogenic | 20 mmHg | 9.5 ± 4.4 | 6 | 3 | 10.6 ± 4.0 | 6 | 3 | t-test | $t(10) = 0.18, p = 0.861$ |
| Sup 4C | Cre-WT | Myogenic | 40 mmHg | 32.3 ± 5.5 | 6 | 3 | 17.5 ± 4.6 | 6 | 3 | t-test | $t(10) = 2.07, p = 0.065$ |
| Sup 4C | Cre-WT | Myogenic | 60 mmHg | 46.9 ± 2.0 | 6 | 3 | 31.9 ± 4.1 | 6 | 3 | Mann-Whitney | $U(N_{ZT7} = 6, N_{ZT19} = 6) = 0$, **$p = 0.002$** |
| Sup 4C | Cre-WT | Myogenic | 80 mmHg | 49.9 ± 2.4 | 6 | 3 | 37.6 ± 2.6 | 6 | 3 | t-test | $t(10) = 3.49$, **$p = 0.006$** |
| Sup 4C | Cre-WT | Myogenic | 100 mmHg | 50.8 ± 2.4 | 6 | 3 | 38.2 ± 3.7 | 6 | 3 | t-test | $t(10) = 2.86$, **$p = 0.017$** |
| Sup 4D | Cre-WT | Phenylephrine | Basal | 34.6 ± 1.9 | 6 | 3 | 30.3 ± 5.2 | 6 | 3 | t-test | $t(10) = 0.78, p = 0.456$ |
| Sup 4D | Cre-WT | Phenylephrine | 1 nmol/L | 36.7 ± 1.5 | 6 | 3 | 28.5 ± 5.3 | 6 | 3 | t-test | $t(10) = 1.48, p = 0.170$ |
| Sup 4D | Cre-WT | Phenylephrine | 10 nmol/L | 38.2 ± 2.6 | 6 | 3 | 27.9 ± 5.5 | 6 | 3 | t-test | $t(10) = 1.68, p = 0.124$ |
| Sup 4D | Cre-WT | Phenylephrine | 100 nmol/L | 48.1 ± 4.0 | 6 | 3 | 38.1 ± 5.1 | 6 | 3 | Mann-Whitney | $U(N_{ZT7} = 6, N_{ZT19} = 6) = 10, p = 0.240$ |
| Sup 4D | Cre-WT | Phenylephrine | 1 µmol/L | 65.2 ± 2.9 | 6 | 3 | 58.9 ± 5.5 | 6 | 3 | t-test | $t(10) = 1.02, p = 0.332$ |
| Sup 4D | Cre-WT | Phenylephrine | 10 µmol/L | 75.9 ± 2.3 | 6 | 3 | 74.0 ± 2.6 | 6 | 3 | t-test | $t(10) = 0.54, p = 0.604$ |
| Sup 4E | Cre-WT | Passive Diameter | 20 mmHg | 79.0 ± 4.4 | 6 | 3 | 83.2 ± 2.3 | 6 | 3 | t-test | $t(10) = 0.84, p = 0.422$ |
| Sup 4E | Cre-WT | Passive Diameter | 40 mmHg | 96.5 ± 3.7 | 6 | 3 | 102.2 ± 5.1 | 6 | 3 | Mann-Whitney | $U(N_{ZT7} = 6, N_{ZT19} = 6) = 12.5, p = 0.420$ |
| Sup 4E | Cre-WT | Passive Diameter | 60 mmHg | 102.7 ± 4.2 | 6 | 3 | 111.2 ± 6.4 | 6 | 3 | t-test | $t(10) = 1.12, p = 0.291$ |
| Sup 4E | Cre-WT | Passive Diameter | 80 mmHg | 105.8 ± 3.9 | 6 | 3 | 115.2 ± 6.7 | 6 | 3 | t-test | $t(10) = 1.20, p = 0.259$ |
| Sup 4E | Cre-WT | Passive Diameter | 100 mmHg | 107.7 ± 4.0 | 6 | 3 | 117.0 ± 6.8 | 6 | 3 | t-test | $t(10) = 1.19, p = 0.262$ |
| Sup 9 | Wild-Type | ERK Phosphorylation | PF670462 | 1.00 ± 0.08 | 10 | 13 | 0.99 ± 0.09 | 10 | 13 | Wilcoxon test | $W(N_{ZT7} = 10, N_{ZT19} = 10) = -7, p = 0.770$ |
| Sup 10A | Wild-Type | Sham Passive Diameter | 20 mmHg | 50.8 ± 2.1 | 9 | 5 | 50.5 ± 2.8 | 6 | 3 | t-test | $t(13) = 0.10, p = 0.922$ |
| Sup 10A | Wild-Type | Sham Passive Diameter | 40 mmHg | 65.1 ± 2.4 | 9 | 5 | 67.9 ± 3.0 | 6 | 3 | t-test | $t(13) = 0.72, p = 0.486$ |
| Sup 10A | Wild-Type | Sham Passive Diameter | 60 mmHg | 70.9 ± 2.6 | 9 | 5 | 74.1 ± 2.7 | 6 | 3 | t-test | $t(13) = 0.83, p = 0.422$ |
| Sup 10A | Wild-Type | Sham Passive Diameter | 80 mmHg | 74.1 ± 2.7 | 9 | 5 | 78.5 ± 2.7 | 6 | 3 | t-test | $t(13) = 1.09, p = 0.297$ |
| Sup 10A | Wild-Type | Sham Passive Diameter | 100 mmHg | 76.3 ± 2.9 | 9 | 5 | 79.2 ± 2.4 | 6 | 3 | t-test | $t(13) = 0.70, p = 0.496$ |
| Sup 10B | Wild-Type | MI Passive Diameter | 20 mmHg | 52.8 ± 4.3 | 7 | 4 | 56.4 ± 4.4 | 7 | 4 | t-test | $t(12) = 0.57, p = 0.577$ |
| Sup 10B | Wild-Type | MI Passive Diameter | 40 mmHg | 64.0 ± 2.6 | 7 | 4 | 69.5 ± 5.1 | 7 | 4 | t-test | $t(12) = 0.95, p = 0.359$ |
| Sup 10B | Wild-Type | MI Passive Diameter | 60 mmHg | 69.9 ± 2.6 | 7 | 4 | 74.8 ± 5.3 | 7 | 4 | Mann-Whitney | $U(N_{ZT7} = 7, N_{ZT19} = 7) = 17, p = 0.383$ |
| Sup 10B | Wild-Type | MI Passive Diameter | 80 mmHg | 73.0 ± 2.9 | 7 | 4 | 78.1 ± 5.6 | 7 | 4 | Mann-Whitney | $U(N_{ZT7} = 7, N_{ZT19} = 7) = 14, p = 0.209$ |
| Sup 10B | Wild-Type | MI Passive Diameter | 100 mmHg | 75.2 ± 2.2 | 7 | 4 | 80.4 ± 5.3 | 7 | 4 | Mann-Whitney | $U(N_{ZT7} = 7, N_{ZT19} = 7) = 14, p = 0.209$ |
| Sup 12A | Wild-Type | Sham Bmal1 mRNA | -- | 5.3 ± 1.4 | 8 | 8 | 11.3 ± 1.8 | 8 | 8 | Mann-Whitney | $U(N_{ZT7} = 8, N_{ZT19} = 8) = 9$, **$p = 0.015$** |
| Sup 12A | $Clock^{\Delta19/\Delta19}$ | Sham Bmal1 mRNA | -- | 5.6 ± 1.3 | 5 | 5 | 7.7 ± 0.6 | 4 | 4 | t-test | $t(7) = 1.33, p = 0.225$ |
| Sup 12B | Wild-Type | MI Bmal1 mRNA | -- | 5.3 ± 0.5 | 7 | 7 | 10.6 ± 1.2 | 7 | 7 | t-test | $t(12) = 4.14$, **$p = 0.001$** |
| Sup 12B | $Clock^{\Delta19/\Delta19}$ | MI Bmal1 mRNA | -- | 10.3 ± 1.1 | 3 | 3 | 11.2 ± 1.7 | 4 | 4 | Mann-Whitney | $U(N_{ZT7} = 3, N_{ZT19} = 4) = 3, p = 0.343$ |

Data are means ± standard error measurements, where n equals the number of measurements (i.e., vessels, western blot samples or RNA samples) and N equals the number of mice used to generate those samples. Statistical significance is defined at $p < 0.05$ (significant p values are highlighted).

*Abbreviations:* Cre-WT = tamoxifen-treated, Cre-expressing wild-type control arteries; MI = myocardial infarction; Sm-Bmal1 KO = smooth muscle specific Bmal1 knockout arteries; TNF KO = tumor necrosis factor knockout arteries; ZT7 = Zeitgeber Time 7; ZT19 = Zeitgeber Time 19.

## Supplemental Table 3: Pre/Post and *in vivo* intervention statistical comparisons

| Figure | Genotype | Parameter | Level | Control | n | N | Intervention | n | N | Comparison Test | Statistics |
|---|---|---|---|---|---|---|---|---|---|---|---|
| 2E | Wild-Type | TNFR1-Fc | -- | 0.83 ± 0.02 | 5 | 5 | 0.95 ± 0.04 | 5 | 5 | t-test | t(8) = 2.55, **p = 0.034** |
| 2F | Wild-Type | Phenylephrine | -- | 0.55 ± 0.06 | 5 | 5 | 0.47 ± 0.10 | 5 | 5 | t-test | t(8) = 0.77, p = 0.464 |
| 2G | Wild-Type | ZT7 Myogenic | 20 mmHg | 10.2 ± 3.5 | 6 | 3 | 10.4 ± 2.5 | 6 | 3 | paired t-test | t(5) = 0.11, p = 0.919 |
| 2G | Wild-Type | ZT7 Myogenic | 40 mmHg | 21.2 ± 2.8 | 6 | 3 | 20.1 ± 3.2 | 6 | 3 | paired t-test | t(5) = 1.13, p = 0.316 |
| 2G | Wild-Type | ZT7 Myogenic | 60 mmHg | 34.9 ± 1.3 | 6 | 3 | 29.8 ± 1.8 | 6 | 3 | paired t-test | t(5) = 3.33, **p = 0.021** |
| 2G | Wild-Type | ZT7 Myogenic | 80 mmHg | 41.3 ± 0.8 | 6 | 3 | 36.0 ± 1.0 | 6 | 3 | paired t-test | t(5) = 9.56, **p < 0.001** |
| 2G | Wild-Type | ZT7 Myogenic | 100 mmHg | 44.6 ± 1.3 | 6 | 3 | 38.2 ± 2.2 | 6 | 3 | paired t-test | t(5) = 4.63, **p = 0.006** |
| 2G | Wild-Type | ZT19 Myogenic | 20 mmHg | 6.6 ± 2.6 | 5 | 3 | 2.9 ± 1.9 | 5 | 3 | Wilcoxon test | W($N_{Con}$ = 5, $N_{CKI-7}$ = 5) = -10, p = 0.125 |
| 2G | Wild-Type | ZT19 Myogenic | 40 mmHg | 15.5 ± 3.2 | 5 | 3 | 10.6 ± 3.7 | 5 | 3 | Wilcoxon test | W($N_{Con}$ = 5, $N_{CKI-7}$ = 5) = -15, p = 0.063 |
| 2G | Wild-Type | ZT19 Myogenic | 60 mmHg | 25.5 ± 3.0 | 5 | 3 | 25.6 ± 3.3 | 5 | 3 | paired t-test | t(4) = 0.61, p = 0.955 |
| 2G | Wild-Type | ZT19 Myogenic | 80 mmHg | 31.2 ± 3.7 | 5 | 3 | 31.6 ± 3.2 | 5 | 3 | paired t-test | t(4) = 0.52, p = 0.628 |
| 2G | Wild-Type | ZT19 Myogenic | 100 mmHg | 35.4 ± 3.8 | 5 | 3 | 36.1 ± 3.0 | 5 | 3 | paired t-test | t(4) = 0.51, p = 0.638 |
| 2H | TNF KO | ZT7 Myogenic | 20 mmHg | 12.9 ± 1.8 | 5 | 3 | 13.5 ± 3.2 | 5 | 3 | paired t-test | t(4) = 0.39, p = 0.717 |
| 2H | TNF KO | ZT7 Myogenic | 40 mmHg | 23.0 ± 4.4 | 5 | 3 | 22.2 ± 4.4 | 5 | 3 | paired t-test | t(4) = 0.62, p = 0.568 |
| 2H | TNF KO | ZT7 Myogenic | 60 mmHg | 35.1 ± 3.7 | 5 | 3 | 35.2 ± 3.1 | 5 | 3 | paired t-test | t(4) = 0.01, p = 0.990 |
| 2H | TNF KO | ZT7 Myogenic | 80 mmHg | 39.2 ± 3.0 | 5 | 3 | 39.7 ± 2.4 | 5 | 3 | paired t-test | t(4) = 0.47, p = 0.664 |
| 2H | TNF KO | ZT7 Myogenic | 100 mmHg | 41.5 ± 2.5 | 5 | 3 | 40.8 ± 1.9 | 5 | 3 | paired t-test | t(4) = 0.94, p = 0.401 |
| 2I | Wild-Type | ZT7 Myogenic | 20 mmHg | 6.9 ± 4.1 | 5 | 4 | 8.6 ± 1.8 | 5 | 4 | t-test | t(8) = 0.37, p = 0.712 |
| 2I | Wild-Type | ZT7 Myogenic | 40 mmHg | 21.8 ± 4.7 | 5 | 4 | 14.3 ± 3.9 | 5 | 4 | t-test | t(8) = 1.24, p = 0.252 |
| 2I | Wild-Type | ZT7 Myogenic | 60 mmHg | 35.1 ± 2.5 | 5 | 4 | 30.9 ± 2.3 | 5 | 4 | t-test | t(8) = 1.23, p = 0.254 |
| 2I | Wild-Type | ZT7 Myogenic | 80 mmHg | 44.4 ± 2.2 | 5 | 4 | 33.3 ± 2.3 | 5 | 4 | t-test | t(8) = 3.51, **p = 0.008** |
| 2I | Wild-Type | ZT7 Myogenic | 100 mmHg | 46.2 ± 1.1 | 5 | 4 | 34.3 ± 2.8 | 5 | 4 | t-test | t(8) = 4.01, **p = 0.004** |
| 2J | Wild-Type | ZT7 Phenylephrine | 1 nmol/L | 23.6 ± 5.9 | 5 | 4 | 23.2 ± 4.8 | 5 | 4 | t-test | t(8) = 0.05, p = 0.961 |
| 2J | Wild-Type | ZT7 Phenylephrine | 10 nmol/L | 28.8 ± 5.1 | 5 | 4 | 21.4 ± 5.2 | 5 | 4 | t-test | t(8) = 1.01, p = 0.342 |
| 2J | Wild-Type | ZT7 Phenylephrine | 100 nmol/L | 35.4 ± 3.9 | 5 | 4 | 30.3 ± 4.9 | 5 | 4 | t-test | t(8) = 0.81, p = 0.439 |
| 2J | Wild-Type | ZT7 Phenylephrine | 1 µmol/L | 56.5 ± 4.0 | 5 | 4 | 47.1 ± 2.1 | 5 | 4 | t-test | t(8) = 2.08, p = 0.071 |
| 2J | Wild-Type | ZT7 Phenylephrine | 10 µmol/L | 74.1 ± 2.3 | 5 | 4 | 68.4 ± 2.4 | 5 | 4 | t-test | t(8) = 1.73, p = 0.121 |
| Sup 6 | Wild-Type | Myogenic | 20 mmHg | 14.0 ± 3.5 | 6 | 3 | 8.7 ± 2.1 | 6 | 3 | paired t-test | t(5) = 2.42, p = 0.060 |
| Sup 6 | Wild-Type | Myogenic | 40 mmHg | 16.9 ± 3.0 | 6 | 3 | 12.4 ± 4.7 | 6 | 3 | paired t-test | t(5) = 2.02, p = 0.100 |
| Sup 6 | Wild-Type | Myogenic | 60 mmHg | 29.5 ± 3.9 | 6 | 3 | 22.6 ± 5.6 | 6 | 3 | paired t-test | t(5) = 1.98, p = 0.105 |
| Sup 6 | Wild-Type | Myogenic | 80 mmHg | 39.3 ± 3.3 | 6 | 3 | 30.7 ± 4.0 | 6 | 3 | paired t-test | t(5) = 2.63, **p = 0.046** |
| Sup 6 | Wild-Type | Myogenic | 100 mmHg | 43.9 ± 2.5 | 6 | 3 | 34.4 ± 3.8 | 6 | 3 | paired t-test | t(5) = 4.79, **p = 0.005** |
| Sup 8 | Wild-Type | ERK Phosphorylation | -- | 1.00 ± 0.10 | 10 | 13 | 1.63 ± 0.16 | 10 | 13 | paired t-test | t(9) = 5.36, **p = 0.001** |

Data are means ± standard error measurements, where n equals the number of measurements (i.e., vessels or western blot samples) and N equals the number of mice used to generate those samples. Statistical significance is defined as p < 0.05 (significant p values are highlighted).

Abbreviations: TNF KO = tumor necrosis factor knockout arteries; TNFR1-Fc = recombinant tumor necrosis factor receptor 1 / Fc fusion protein decoy construct; ZT7 = Zeitgeber Time 7; ZT19 = Zeitgeber Time 19.

## Supplemental Table 4: Sham / myocardial infarction statistical comparisons

| Figure | Genotype | Parameter | Level | Sham | n | N | MI | n | N | Comparison Test | Statistics |
|---|---|---|---|---|---|---|---|---|---|---|---|
| 4A | Clock$^{\Delta19/\Delta19}$ | ZT7 Myogenic | 20 mmHg | 14.3 ± 3.6 | 7 | 4 | 18.1 ± 3.0 | 6 | 4 | t-test | t(11) = 0.79, p = 0.448 |
| 4A | Clock$^{\Delta19/\Delta19}$ | ZT7 Myogenic | 40 mmHg | 22.7 ± 4.1 | 7 | 4 | 25.1 ± 3.5 | 6 | 4 | t-test | t(11) = 0.43, p = 0.674 |
| 4A | Clock$^{\Delta19/\Delta19}$ | ZT7 Myogenic | 60 mmHg | 28.5 ± 4.1 | 7 | 4 | 32.1 ± 2.8 | 6 | 4 | t-test | t(11) = 0.69, p = 0.505 |
| 4A | Clock$^{\Delta19/\Delta19}$ | ZT7 Myogenic | 80 mmHg | 32.7 ± 4.9 | 7 | 4 | 36.4 ± 1.9 | 6 | 4 | Mann-Whitney | U($N_{Sham}$ = 7, $N_{MI}$ = 6) = 18, p = 0.731 |
| 4A | Clock$^{\Delta19/\Delta19}$ | ZT7 Myogenic | 100 mmHg | 36.0 ± 2.8 | 7 | 4 | 38.9 ± 2.2 | 6 | 4 | t-test | t(11) = 0.80, p = 0.440 |
| 4B | Clock$^{\Delta19/\Delta19}$ | ZT19 Myogenic | 20 mmHg | 19.7 ± 3.1 | 7 | 4 | 20.1 ± 4.5 | 8 | 4 | t-test | t(13) = 0.57, p = 0.956 |
| 4B | Clock$^{\Delta19/\Delta19}$ | ZT19 Myogenic | 40 mmHg | 22.9 ± 3.6 | 7 | 4 | 28.9 ± 4.1 | 8 | 4 | t-test | t(13) = 1.09, p = 0.297 |
| 4B | Clock$^{\Delta19/\Delta19}$ | ZT19 Myogenic | 60 mmHg | 30.7 ± 3.6 | 7 | 4 | 34.0 ± 2.2 | 8 | 4 | t-test | t(13) = 0.82, p = 0.428 |
| 4B | Clock$^{\Delta19/\Delta19}$ | ZT19 Myogenic | 80 mmHg | 34.6 ± 2.7 | 7 | 4 | 37.8 ± 2.8 | 8 | 4 | t-test | t(13) = 0.80, p = 0.436 |
| 4B | Clock$^{\Delta19/\Delta19}$ | ZT19 Myogenic | 100 mmHg | 36.9 ± 2.6 | 7 | 4 | 38.3 ± 3.4 | 8 | 4 | t-test | t(13) = 0.33, p = 0.750 |
| 4C | Clock$^{\Delta19/\Delta19}$ | ZT7 Phenylephrine | Basal | 13.9 ± 4.5 | 7 | 4 | 18.6 ± 3.7 | 8 | 4 | t-test | t(13) = 0.80, p = 0.436 |
| 4C | Clock$^{\Delta19/\Delta19}$ | ZT7 Phenylephrine | 1 nmol/L | 23.0 ± 4.6 | 7 | 4 | 20.2 ± 2.8 | 8 | 4 | t-test | t(13) = 0.53, p = 0.605 |
| 4C | Clock$^{\Delta19/\Delta19}$ | ZT7 Phenylephrine | 10 nmol/L | 23.5 ± 4.4 | 7 | 4 | 24.2 ± 2.5 | 8 | 4 | t-test | t(13) = 0.13, p = 0.895 |
| 4C | Clock$^{\Delta19/\Delta19}$ | ZT7 Phenylephrine | 100 nmol/L | 34.7 ± 5.5 | 7 | 4 | 33.1 ± 3.4 | 8 | 4 | Mann-Whitney | U($N_{Sham}$ = 7, $N_{MI}$ = 8) = 21, p = 0.463 |
| 4C | Clock$^{\Delta19/\Delta19}$ | ZT7 Phenylephrine | 1 µmol/L | 62.9 ± 3.5 | 7 | 4 | 53.9 ± 3.2 | 8 | 4 | t-test | t(13) = 1.90, p = 0.080 |
| 4C | Clock$^{\Delta19/\Delta19}$ | ZT7 Phenylephrine | 10 µmol/L | 74.5 ± 0.9 | 7 | 4 | 69.6 ± 3.2 | 8 | 4 | Mann-Whitney | U($N_{Sham}$ = 7, $N_{MI}$ = 8) = 18, p = 0.281 |
| 4D | Clock$^{\Delta19/\Delta19}$ | ZT19 Phenylephrine | Basal | 16.4 ± 3.4 | 7 | 4 | 19.4 ± 3.2 | 8 | 4 | t-test | t(13) = 0.64, p = 0.535 |
| 4D | Clock$^{\Delta19/\Delta19}$ | ZT19 Phenylephrine | 1 nmol/L | 21.2 ± 3.4 | 7 | 4 | 24.7 ± 3.3 | 8 | 4 | Mann-Whitney | U($N_{Sham}$ = 7, $N_{MI}$ = 8) = 21, p = 0.463 |
| 4D | Clock$^{\Delta19/\Delta19}$ | ZT19 Phenylephrine | 10 nmol/L | 23.7 ± 3.1 | 7 | 4 | 25.3 ± 1.9 | 8 | 4 | t-test | t(13) = 0.45, p = 0.658 |
| 4D | Clock$^{\Delta19/\Delta19}$ | ZT19 Phenylephrine | 100 nmol/L | 32.8 ± 3.5 | 7 | 4 | 36.6 ± 3.0 | 8 | 4 | t-test | t(13) = 0.84, p = 0.417 |
| 4D | Clock$^{\Delta19/\Delta19}$ | ZT19 Phenylephrine | 1 µmol/L | 55.9 ± 2.1 | 7 | 4 | 59.2 ± 2.4 | 8 | 4 | t-test | t(13) = 1.01, p = 0.332 |
| 4D | Clock$^{\Delta19/\Delta19}$ | ZT19 Phenylephrine | 10 µmol/L | 72.2 ± 2.4 | 7 | 4 | 73.1 ± 1.9 | 8 | 4 | t-test | t(13) = 0.31, p = 0.759 |
| 5A | Cre-WT | ZT19 Myogenic | 20 mmHg | 5.3 ± 1.6 | 7 | 4 | 20.7 ± 4.4 | 7 | 5 | Mann-Whitney | U($N_{Sham}$ = 7, $N_{MI}$ = 7) = 6, **p = 0.017** |
| 5A | Cre-WT | ZT19 Myogenic | 40 mmHg | 15.1 ± 3.5 | 7 | 4 | 30.5 ± 4.1 | 7 | 5 | t-test | t(12) = 2.86, **p = 0.014** |
| 5A | Cre-WT | ZT19 Myogenic | 60 mmHg | 23.7 ± 2.6 | 7 | 4 | 35.2 ± 3.9 | 7 | 5 | t-test | t(12) = 2.46, **p = 0.030** |
| 5A | Cre-WT | ZT19 Myogenic | 80 mmHg | 29.1 ± 4.4 | 7 | 4 | 39.8 ± 3.5 | 7 | 5 | t-test | t(12) = 1.92, p = 0.079 |
| 5A | Cre-WT | ZT19 Myogenic | 100 mmHg | 27.3 ± 4.9 | 7 | 4 | 41.5 ± 3.1 | 7 | 5 | t-test | t(12) = 2.44, **p = 0.031** |
| 5B | Sm-Bmal1 KO | ZT19 Myogenic | 20 mmHg | 7.8 ± 2.4 | 7 | 4 | 11.3 ± 4.2 | 7 | 5 | t-test | t(12) = 0.73, p = 0.479 |
| 5B | Sm-Bmal1 KO | ZT19 Myogenic | 40 mmHg | 13.4 ± 5.5 | 7 | 4 | 17.9 ± 5.3 | 7 | 5 | t-test | t(12) = 0.59, p = 0.569 |
| 5B | Sm-Bmal1 KO | ZT19 Myogenic | 60 mmHg | 20.6 ± 3.8 | 7 | 4 | 22.1 ± 5.9 | 7 | 5 | Mann-Whitney | U($N_{Sham}$ = 7, $N_{MI}$ = 7) = 24, p = 1.000 |
| 5B | Sm-Bmal1 KO | ZT19 Myogenic | 80 mmHg | 27.0 ± 3.1 | 7 | 4 | 23.9 ± 6.0 | 7 | 5 | t-test | t(12) = 0.46, p = 0.655 |
| 5B | Sm-Bmal1 KO | ZT19 Myogenic | 100 mmHg | 23.8 ± 3.6 | 7 | 4 | 26.1 ± 5.7 | 7 | 5 | t-test | t(12) = 0.34, p = 0.739 |
| 5C | Cre-WT | ZT19 Phenylephrine | Basal | 24.7 ± 2.7 | 7 | 4 | 24.8 ± 7.1 | 7 | 5 | Mann-Whitney | U($N_{Sham}$ = 7, $N_{MI}$ = 7) = 22, p = 0.805 |
| 5C | Cre-WT | ZT19 Phenylephrine | 1 nmol/L | 25.6 ± 3.5 | 7 | 4 | 25.4 ± 6.6 | 7 | 5 | t-test | t(12) = 0.02, p = 0.988 |
| 5C | Cre-WT | ZT19 Phenylephrine | 10 nmol/L | 24.5 ± 3.8 | 7 | 4 | 25.7 ± 6.7 | 7 | 5 | t-test | t(12) = 0.16, p = 0.878 |
| 5C | Cre-WT | ZT19 Phenylephrine | 100 nmol/L | 29.6 ± 4.5 | 7 | 4 | 27.3 ± 7.0 | 7 | 5 | t-test | t(12) = 0.28, p = 0.787 |
| 5C | Cre-WT | ZT19 Phenylephrine | 1 µmol/L | 52.5 ± 5.2 | 7 | 4 | 39.2 ± 7.9 | 7 | 5 | t-test | t(12) = 1.40, p = 0.187 |
| 5C | Cre-WT | ZT19 Phenylephrine | 10 µmol/L | 69.5 ± 3.6 | 7 | 4 | 67.7 ± 4.3 | 7 | 5 | t-test | t(12) = 0.32, p = 0.756 |
| 5D | Sm-Bmal1 KO | ZT19 Phenylephrine | Basal | 14.6 ± 3.2 | 7 | 4 | 21.8 ± 5.0 | 7 | 5 | t-test | t(12) = 1.22, p = 0.248 |
| 5D | Sm-Bmal1 KO | ZT19 Phenylephrine | 1 nmol/L | 15.7 ± 3.9 | 7 | 4 | 19.1 ± 4.4 | 7 | 5 | t-test | t(12) = 0.58, p = 0.547 |
| 5D | Sm-Bmal1 KO | ZT19 Phenylephrine | 10 nmol/L | 17.6 ± 4.1 | 7 | 4 | 23.8 ± 4.2 | 7 | 5 | t-test | t(12) = 1.07, p = 0.308 |
| 5D | Sm-Bmal1 KO | ZT19 Phenylephrine | 100 nmol/L | 23.2 ± 5.9 | 7 | 4 | 35.2 ± 6.2 | 7 | 5 | t-test | t(12) = 1.39, p = 0.189 |
| 5D | Sm-Bmal1 KO | ZT19 Phenylephrine | 1 µmol/L | 46.9 ± 8.3 | 7 | 4 | 47.6 ± 8.9 | 7 | 5 | t-test | t(12) = 0.05, p = 0.959 |
| 5D | Sm-Bmal1 KO | ZT19 Phenylephrine | 10 µmol/L | 73.5 ± 3.2 | 7 | 4 | 72.4 ± 3.3 | 7 | 5 | t-test | t(12) = 0.24, p = 0.815 |
| Sup 11 | Wild-Type | TNF mRNA | -- | 1.00 ± 0.16 | 6 | 6 | 1.09 ± 0.25 | 6 | 6 | Mann-Whitney | U($N_{Sham}$ = 6, $N_{MI}$ = 6) = 17, p = 0.905 |
| Sup 11 | Wild-Type | TNFR1 mRNA | -- | 1.00 ± 0.06 | 24 | 24 | 1.06 ± 0.09 | 18 | 18 | t-test | t(40) = 0.60, p = 0.553 |
| Sup 11 | Wild-Type | TNFR2 mRNA | -- | 1.00 ± 0.06 | 24 | 24 | 0.90 ± 0.07 | 18 | 18 | t-test | t(40) = 1.15, p = 0.259 |
| Sup 11 | Wild-Type | CK1 delta mRNA | -- | 1.00 ± 0.04 | 12 | 12 | 0.98 ± 0.07 | 6 | 6 | t-test | t(16) = 0.28, p = 0.784 |
| Sup 11 | Wild-Type | CK1 epsilon mRNA | -- | 1.00 ± 0.06 | 12 | 12 | 0.99 ± 0.04 | 6 | 6 | t-test | t(16) = 0.14, p = 0.890 |
| Sup 13A | Clock$^{\Delta19/\Delta19}$ | ZT7 Passive Diameter | 20 mmHg | 65.2 ± 3.6 | 7 | 4 | 62.4 ± 5.4 | 6 | 4 | t-test | t(11) = 0.45, p = 0.663 |
| Sup 13A | Clock$^{\Delta19/\Delta19}$ | ZT7 Passive Diameter | 40 mmHg | 77.3 ± 3.7 | 7 | 4 | 75.1 ± 5.4 | 6 | 4 | t-test | t(11) = 0.34, p = 0.738 |
| Sup 13A | Clock$^{\Delta19/\Delta19}$ | ZT7 Passive Diameter | 60 mmHg | 81.4 ± 3.9 | 7 | 4 | 79.7 ± 5.6 | 6 | 4 | t-test | t(11) = 0.25, p = 0.810 |
| Sup 13A | Clock$^{\Delta19/\Delta19}$ | ZT7 Passive Diameter | 80 mmHg | 83.7 ± 4.2 | 7 | 4 | 81.5 ± 5.9 | 6 | 4 | t-test | t(11) = 0.32, p = 0.757 |
| Sup 13A | Clock$^{\Delta19/\Delta19}$ | ZT7 Passive Diameter | 100 mmHg | 85.1 ± 4.6 | 7 | 4 | 84.5 ± 6.1 | 6 | 4 | t-test | t(11) = 0.08, p = 0.936 |
| Sup 13B | Clock$^{\Delta19/\Delta19}$ | ZT19 Passive Diameter | 20 mmHg | 59.5 ± 3.4 | 7 | 4 | 56.4 ± 3.7 | 8 | 4 | t-test | t(13) = 0.61, p = 0.555 |
| Sup 13B | Clock$^{\Delta19/\Delta19}$ | ZT19 Passive Diameter | 40 mmHg | 67.5 ± 3.0 | 7 | 4 | 72.3 ± 4.7 | 8 | 4 | t-test | t(13) = 0.85, p = 0.411 |
| Sup 13B | Clock$^{\Delta19/\Delta19}$ | ZT19 Passive Diameter | 60 mmHg | 71.9 ± 2.9 | 7 | 4 | 76.6 ± 4.9 | 8 | 4 | t-test | t(13) = 0.79, p = 0.441 |
| Sup 13B | Clock$^{\Delta19/\Delta19}$ | ZT19 Passive Diameter | 80 mmHg | 75.0 ± 2.8 | 7 | 4 | 79.5 ± 5.0 | 8 | 4 | t-test | t(13) = 0.75, p = 0.468 |
| Sup 13B | Clock$^{\Delta19/\Delta19}$ | ZT19 Passive Diameter | 100 mmHg | 75.0 ± 3.1 | 7 | 4 | 82.6 ± 5.3 | 8 | 4 | Mann-Whitney | U($N_{Sham}$ = 7, $N_{MI}$ = 8) = 14, p = 0.121 |

*...Supplemental Table 4 continues on the next page*

| Figure | Genotype | Parameter | Level | Sham | n | N | MI | n | N | Comparison Test | Statistics |
|---|---|---|---|---|---|---|---|---|---|---|---|
| Sup 14A | Cre-WT | ZT19 Passive Diameter | 20 mmHg | 52.2 ± 5.4 | 7 | 4 | 59.5 ± 5.9 | 7 | 5 | t-test | t(12) = 0.92, p = 0.377 |
| Sup 14A | Cre-WT | ZT19 Passive Diameter | 40 mmHg | 65.8 ± 4.9 | 7 | 4 | 71.5 ± 6.0 | 7 | 5 | t-test | t(12) = 0.74, p = 0.475 |
| Sup 14A | Cre-WT | ZT19 Passive Diameter | 60 mmHg | 70.0 ± 5.0 | 7 | 4 | 74.6 ± 6.2 | 7 | 5 | t-test | t(12) = 0.57, p = 0.579 |
| Sup 14A | Cre-WT | ZT19 Passive Diameter | 80 mmHg | 71.9 ± 4.5 | 7 | 4 | 77.7 ± 6.3 | 7 | 5 | t-test | t(12) = 0.74, p = 0.471 |
| Sup 14A | Cre-WT | ZT19 Passive Diameter | 100 mmHg | 73.1 ± 4.3 | 7 | 4 | 81.0 ± 6.6 | 7 | 5 | t-test | t(12) = 1.00, p = 0.335 |
| Sup 14B | Sm-Bmal1 KO | ZT19 Passive Diameter | 20 mmHg | 45.2 ± 5.4 | 7 | 4 | 45.0 ± 4.2 | 7 | 5 | t-test | t(12) = 0.03, p = 0.979 |
| Sup 14B | Sm-Bmal1 KO | ZT19 Passive Diameter | 40 mmHg | 57.5 ± 6.7 | 7 | 4 | 55.9 ± 4.2 | 7 | 5 | t-test | t(12) = 0.21, p = 0.840 |
| Sup 14B | Sm-Bmal1 KO | ZT19 Passive Diameter | 60 mmHg | 60.8 ± 6.8 | 7 | 4 | 60.3 ± 4.2 | 7 | 5 | t-test | t(12) = 0.06, p = 0.950 |
| Sup 14B | Sm-Bmal1 KO | ZT19 Passive Diameter | 80 mmHg | 63.4 ± 6.8 | 7 | 4 | 62.2 ± 4.4 | 7 | 5 | t-test | t(12) = 0.15, p = 0.883 |
| Sup 14B | Sm-Bmal1 KO | ZT19 Passive Diameter | 100 mmHg | 65.6 ± 6.8 | 7 | 4 | 64.0 ± 4.5 | 7 | 5 | t-test | t(12) = 0.20, p = 0.843 |

Data are means ± standard error measurements, where n equals the number of measurements (i.e., vessels) and N equals the number of mice used to generate those samples. Statistical significance is defined as $p < 0.05$ (significant p values are highlighted).

*Abbreviations:* CK1 = casein kinase 1; Cre-WT = tamoxifen-treated, Cre-expressing wild-type control arteries; MI = myocardial infarction; Sm-Bmal1 KO = smooth muscle specific Bmal1 knockout arteries; TNF = tumor necrosis factor; TNFR = tumor necrosis factor receptor; ZT7 = Zeitgeber Time 7; ZT19 = Zeitgeber Time 19.

## Supplemental Table 5: Wild-type / mutant mouse statistical comparisons

| Figure | Genotype | Parameter | Time Point | WT | n | N | Mutant | n | N | Comparison Test | Statistics |
|---|---|---|---|---|---|---|---|---|---|---|---|
| 4E | WT / Clock$^{\Delta19/\Delta19}$ | Sham TPR | 8 weeks | 5.49 ± 0.22 | 8 | 8 | 5.46 ± 0.17 | 8 | 8 | t-test | t(14) = 0.11, p = 0.912 |
| 4E | WT / Clock$^{\Delta19/\Delta19}$ | MI TPR | 8 weeks | 6.88 ± 0.24 | 8 | 8 | 5.48 ± 0.19 | 8 | 8 | t-test | t(14) = 4.56, **p < 0.001** |
| 4F | WT / Clock$^{\Delta19/\Delta19}$ | Sham MAP | 8 weeks | 74.6 ± 0.4 | 8 | 8 | 73.3 ± 0.7 | 8 | 8 | t-test | t(14) = 1.42, p = 0.179 |
| 4F | WT / Clock$^{\Delta19/\Delta19}$ | MI MAP | 8 weeks | 68.7 ± 0.5 | 8 | 8 | 66.6 ± 0.7 | 8 | 8 | t-test | t(14) = 2.43, **p = 0.029** |
| 4G | WT / Clock$^{\Delta19/\Delta19}$ | Sham CO | 8 weeks | 13.7 ± 0.5 | 8 | 8 | 13.5 ± 0.4 | 8 | 8 | t-test | t(14) = 0.31, p = 0.761 |
| 4G | WT / Clock$^{\Delta19/\Delta19}$ | MI CO | 8 weeks | 10.1 ± 0.4 | 8 | 8 | 12.3 ± 0.4 | 8 | 8 | t-test | t(14) = 4.08, **p = 0.001** |
| 4H | WT / Clock$^{\Delta19/\Delta19}$ | Sham EF | 8 weeks | 76.2 ± 0.9 | 8 | 8 | 76.5 ± 0.4 | 8 | 8 | t-test | t(14) = 0.27, p = 0.790 |
| 4H | WT / Clock$^{\Delta19/\Delta19}$ | MI EF | 1 week | 59.7 ± 1.6 | 8 | 8 | 60.8 ± 1.6 | 8 | 8 | t-test | t(14) = 0.51, p = 0.618 |
| 4H | WT / Clock$^{\Delta19/\Delta19}$ | MI EF | 8 weeks | 49.9 ± 1.2 | 8 | 8 | 57.7 ± 1.7 | 8 | 8 | t-test | t(14) = 3.81, **p = 0.002** |
| 4I | WT / Clock$^{\Delta19/\Delta19}$ | Sham LVIDs | 8 weeks | 2.46 ± 0.05 | 8 | 8 | 2.44 ± 0.01 | 8 | 8 | Mann-Whitney | U($N_{WT}$ = 8, $N_{Clock}$ = 8) = 31, p = 0.934 |
| 4I | WT / Clock$^{\Delta19/\Delta19}$ | MI LVIDs | 1 week | 3.41 ± 0.09 | 8 | 8 | 3.35 ± 0.09 | 8 | 8 | t-test | t(14) = 0.49, p = 0.635 |
| 4I | WT / Clock$^{\Delta19/\Delta19}$ | MI LVIDs | 8 weeks | 4.18 ± 0.11 | 8 | 8 | 3.62 ± 0.10 | 8 | 8 | t-test | t(14) = 3.71, **p = 0.002** |
| 4J | WT / Clock$^{\Delta19/\Delta19}$ | Sham LVIDd | 8 weeks | 4.05 ± 0.05 | 8 | 8 | 4.04 ± 0.02 | 8 | 8 | t-test | t(14) = 0.14, p = 0.894 |
| 4J | WT / Clock$^{\Delta19/\Delta19}$ | MI LVIDd | 1 week | 4.70 ± 0.07 | 8 | 8 | 4.65 ± 0.07 | 8 | 8 | t-test | t(14) = 0.46, p = 0.653 |
| 4J | WT / Clock$^{\Delta19/\Delta19}$ | MI LVIDd | 8 weeks | 5.31 ± 0.10 | 8 | 8 | 4.90 ± 0.08 | 8 | 8 | t-test | t(14) = 3.30, **p = 0.005** |
| 4K | WT / Clock$^{\Delta19/\Delta19}$ | Infarct Expansion | 8 weeks | 47.8 ± 3.1 | 4 | 4 | 37.1 ± 2.9 | 4 | 4 | t-test | t(6) = 2.50, **p = 0.047** |
| 5E | WT / Sm-Bmal1 KO | Sham TPR | 8 weeks | 5.9 ± 0.3 | 5 | 5 | 5.8 ± 0.3 | 5 | 5 | Mann-Whitney | U($N_{WT}$ = 5, $N_{KO}$ = 5) = 9, p = 0.548 |
| 5E | WT / Sm-Bmal1 KO | MI TPR | 8 weeks | 7.4 ± 0.3 | 6 | 6 | 6.3 ± 0.2 | 9 | 6 | t-test | t(13) = 3.58, **p = 0.003** |
| 5F | WT / Sm-Bmal1 KO | Sham MAP | 8 weeks | 76.5 ± 1.7 | 5 | 5 | 77.8 ± 2.2 | 5 | 5 | t-test | t(8) = 0.45, p = 0.664 |
| 5F | WT / Sm-Bmal1 KO | MI MAP | 8 weeks | 70.9 ± 0.7 | 6 | 6 | 74.5 ± 1.0 | 9 | 6 | t-test | t(13) = 2.63, **p = 0.021** |
| 5G | WT / Sm-Bmal1 KO | Sham CO | 8 weeks | 13.1 ± 0.5 | 5 | 5 | 13.5 ± 0.4 | 5 | 5 | t-test | t(8) = 0.64, p = 0.537 |
| 5G | WT / Sm-Bmal1 KO | MI CO | 8 weeks | 9.7 ± 0.4 | 6 | 6 | 11.9 ± 0.4 | 9 | 6 | t-test | t(13) = 4.12, **p = 0.001** |
| 5H | WT / Sm-Bmal1 KO | Sham EF | 8 weeks | 69.4 ± 2.4 | 8 | 8 | 72.3 ± 1.2 | 9 | 8 | t-test | t(15) = 1.13, p = 0.276 |
| 5H | WT / Sm-Bmal1 KO | MI EF | 1 week | 45.1 ± 5.4 | 6 | 6 | 57.1 ± 3.8 | 10 | 6 | t-test | t(14) = 1.85, p = 0.085 |
| 5H | WT / Sm-Bmal1 KO | MI EF | 8 weeks | 43.0 ± 4.1 | 6 | 6 | 55.1 ± 3.2 | 12 | 6 | t-test | t(16) = 2.24, **p = 0.039** |
| 5I | WT / Sm-Bmal1 KO | Sham LVIDs | 8 weeks | 2.6 ± 0.1 | 8 | 8 | 2.6 ± 0.1 | 9 | 8 | Mann-Whitney | U($N_{WT}$ = 8, $N_{KO}$ = 9) = 29, p = 0.523 |
| 5I | WT / Sm-Bmal1 KO | MI LVIDs | 1 week | 3.8 ± 0.3 | 6 | 6 | 3.1 ± 0.2 | 10 | 6 | t-test | t(14) = 2.21, **p = 0.044** |
| 5I | WT / Sm-Bmal1 KO | MI LVIDs | 8 weeks | 4.1 ± 0.2 | 6 | 6 | 3.3 ± 0.2 | 12 | 6 | t-test | t(16) = 3.09, **p = 0.007** |
| 5J | WT / Sm-Bmal1 KO | Sham LVIDd | 8 weeks | 3.9 ± 0.1 | 8 | 8 | 4.0 ± 0.1 | 9 | 8 | Mann-Whitney | U($N_{WT}$ = 8, $N_{KO}$ = 9) = 24, p = 0.264 |
| 5J | WT / Sm-Bmal1 KO | MI LVIDd | 1 week | 4.7 ± 0.2 | 6 | 6 | 4.2 ± 0.1 | 10 | 6 | t-test | t(14) = 2.14, p = 0.051 |
| 5J | WT / Sm-Bmal1 KO | MI LVIDd | 8 weeks | 5.0 ± 0.2 | 6 | 6 | 4.3 ± 0.1 | 12 | 6 | Mann-Whitney | U($N_{WT}$ = 6, $N_{KO}$ = 12) = 8.5, **p = 0.007** |
| 5K | WT / Sm-Bmal1 KO | Infarct Expansion | 8 weeks | 43.9 ± 3.7 | 3 | 3 | 20.8 ± 3.5 | 3 | 3 | t-test | t(4) = 4.58, **p = 0.010** |

Data are means ± standard error measurements, where n equals the number of measurements from N mice. Statistical significance is defined as $p < 0.05$ (significant p values are highlighted).

*Abbreviations:* CO = cardiac output; EF = ejection fraction; LVIDd = left ventricle internal dimensions at diastole; LVIDs = left ventricle internal dimensions at systole MAP = mean arterial pressure; MI = myocardial infarction; Sm-Bmal1 KO = smooth muscle specific Bmal1 knockout arteries; TPR = total peripheral resistance.

## Supplemental Table 6: JTK_CYCLE analysis - wild-type artery pressure myography data

| Figure | Parameter | Level | ZT3 | n | N | ZT7 | n | N | ZT11 | n | N | ZT15 | n | N | ZT19 | n | N | ZT23 | n | N | JTK P | Period | Acrophase | Amplitude |
|---|---|---|---|---|---|---|---|---|---|---|---|---|---|---|---|---|---|---|---|---|---|---|---|---|
| 1A | Myogenic | 20 mmHg | 19.2 ± 5.6 | 5 | 5 | 37.0 ± 3.1 | 4 | 3 | 32.2 ± 3.1 | 4 | 3 | 19.4 ± 4.4 | 6 | 3 | 24.7 ± 6.5 | 6 | 3 | 22.3 ± 5.6 | 6 | 4 | 0.626 | 24 | 6 | 5.09 |
| 1A | Myogenic | 40 mmHg | 35.4 ± 2.1 | 5 | 5 | 39.1 ± 4.3 | 4 | 3 | 39.3 ± 2.3 | 4 | 3 | 32.4 ± 3.5 | 6 | 3 | 34.0 ± 4.5 | 6 | 3 | 31.3 ± 3.8 | 6 | 4 | 0.541 | 24 | 6 | 3.92 |
| 1A | Myogenic | 60 mmHg | 44.0 ± 2.4 | 5 | 5 | 48.0 ± 2.6 | 4 | 3 | 47.8 ± 2.3 | 4 | 3 | 38.3 ± 2.2 | 6 | 3 | 35.6 ± 4.2 | 6 | 3 | 35.6 ± 3.5 | 6 | 4 | **0.002** * | 24 | 6 | 6.97 |
| 1A | Myogenic | 80 mmHg | 46.0 ± 2.4 | 5 | 5 | 53.8 ± 2.1 | 4 | 3 | 50.3 ± 1.8 | 4 | 3 | 42.8 ± 1.8 | 6 | 3 | 39.7 ± 2.5 | 6 | 3 | 37.2 ± 3.9 | 6 | 4 | **< 0.001** * | 24 | 6 | 6.63 |
| 1A | Myogenic | 100 mmHg | 46.4 ± 2.6 | 5 | 5 | 54.4 ± 2.8 | 4 | 3 | 51.0 ± 2.1 | 4 | 3 | 44.8 ± 2.4 | 6 | 3 | 40.7 ± 3.1 | 6 | 3 | 36.9 ± 5.2 | 6 | 4 | **0.003** * | 24 | 6 | 6.31 |
| 1C | Phenylephrine | 1 nmol/L | 40.8 ± 2.8 | 5 | 5 | 42.5 ± 3.4 | 4 | 3 | 30.6 ± 8.7 | 4 | 4 | 32.1 ± 2.7 | 6 | 3 | 27.8 ± 4.6 | 6 | 3 | 37.9 ± 3.8 | 6 | 4 | 0.132 | 24 | 6 | 5.09 |
| 1C | Phenylephrine | 10 nmol/L | 46.6 ± 3 | 5 | 5 | 50.8 ± 2.6 | 4 | 3 | 36.6 ± 10 | 4 | 4 | 38.1 ± 2.8 | 6 | 3 | 35.1 ± 5.3 | 6 | 3 | 40.0 ± 4.5 | 6 | 4 | 0.451 | 24 | 6 | 5.94 |
| 1C | Phenylephrine | 100 nmol/L | 57.2 ± 3.8 | 5 | 5 | 62.9 ± 2.2 | 4 | 3 | 55.5 ± 7.7 | 4 | 4 | 44.1 ± 2.6 | 6 | 3 | 50.6 ± 6.5 | 6 | 3 | 46.4 ± 5.1 | 6 | 4 | 0.238 | 24 | 6 | 8.98 |
| 1C | Phenylephrine | 1 µmol/L | 65.1 ± 3.8 | 5 | 5 | 72.7 ± 2.3 | 4 | 3 | 73.0 ± 2 | 4 | 4 | 62.5 ± 3.1 | 6 | 3 | 67.9 ± 4.5 | 6 | 3 | 58.5 ± 7.1 | 6 | 4 | 0.368 | 24 | 6 | 3.75 |
| 1C | Phenylephrine | 10 µmol/L | 77.8 ± 3.4 | 5 | 5 | 75.1 ± 3.3 | 4 | 3 | 77.9 ± 2.9 | 4 | 4 | 70.6 ± 3.6 | 6 | 3 | 76.0 ± 3.5 | 6 | 3 | 64.9 ± 6.6 | 6 | 4 | 1.000 | 24 | 6 | 4.24 |
| Sup 2 | KCl | 60 mmol/L | 76.7 ± 2.9 | 4 | 4 | 78.1 ± 2.9 | 4 | 3 | 77.0 ± 2.5 | 6 | 4 | 73.6 ± 4 | 6 | 3 | 70.8 ± 2.9 | 6 | 3 | 85.6 ± 1.8 | 4 | 3 | 0.397 | 24 | 6 | 4.74 |

Data are means ± standard error measurements, where n equals the number of measurements (i.e., vessels) and N equals the number of mice used to generate those samples. *JTK P* is the Bonferroni-adjusted minimal P value calculated by JTK_CYCLE. *Acrophase* is the Zeitgeber time of the sinusoidal rhythm peak calculated by JTK_CYCLE. * denotes a statistically significant circadian rhythm ($P<0.05$).

*Abbreviations:* KCl = potassium chloride; ZT = Zeitgeber time.

## Supplemental Table 7: JTK_CYCLE analysis - mutant mouse artery pressure myography data

| Figure | Genotype | Parameter | Level | ZT3 | n | N | ZT7 | n | N | ZT11 | n | N | ZT15 | n | N | ZT19 | n | N | ZT23 | n | N | JTK P | Period | Acrophase | Amplitude |
|---|---|---|---|---|---|---|---|---|---|---|---|---|---|---|---|---|---|---|---|---|---|---|---|---|---|
| Sup 4A | Cre-WT | Myogenic | 20 mmHg | 14.8 ± 5.0 | 6 | 3 | 9.5 ± 4.4 | 6 | 3 | 4.9 ± 1.3 | 6 | 3 | 2.0 ± 1.0 | 6 | 3 | 10.6 ± 4.0 | 6 | 3 | 9.5 ± 3.3 | 6 | 3 | 0.624 | 24 | 0 | 3.57 |
| Sup 4A | Cre-WT | Myogenic | 40 mmHg | 27.2 ± 5.7 | 6 | 3 | 32.3 ± 5.5 | 6 | 3 | 24.9 ± 2.8 | 6 | 3 | 16.6 ± 3.2 | 6 | 3 | 17.5 ± 4.6 | 6 | 3 | 20.9 ± 5.7 | 6 | 3 | 0.167 | 24 | 4 | 7.22 |
| Sup 4A | Cre-WT | Myogenic | 60 mmHg | 41.0 ± 2.7 | 6 | 3 | 46.9 ± 2.0 | 6 | 3 | 40.2 ± 3.5 | 6 | 3 | 34.2 ± 2.1 | 6 | 3 | 31.9 ± 4.1 | 6 | 3 | 36.3 ± 2.9 | 6 | 3 | **0.014** * | 24 | 4 | 5.73 |
| Sup 4A | Cre-WT | Myogenic | 80 mmHg | 47.5 ± 2.7 | 6 | 3 | 49.9 ± 2.4 | 6 | 3 | 44.4 ± 4.0 | 6 | 3 | 39.9 ± 2.5 | 6 | 3 | 37.6 ± 2.6 | 6 | 3 | 43.3 ± 2.8 | 6 | 3 | **0.011** * | 24 | 4 | 5.14 |
| Sup 4A | Cre-WT | Myogenic | 100 mmHg | 49.9 ± 1.9 | 6 | 3 | 50.8 ± 2.4 | 6 | 3 | 47.1 ± 3.9 | 6 | 3 | 42.6 ± 2.8 | 6 | 3 | 38.2 ± 3.7 | 6 | 3 | 45.6 ± 2.7 | 6 | 3 | 0.052 | 24 | 4 | 4.51 |
| Sup 4B | Cre-WT | Phenylephrine | 1 nmol/L | 30.2 ± 3.0 | 6 | 3 | 36.7 ± 1.5 | 6 | 3 | 35.4 ± 3.2 | 6 | 3 | 19.4 ± 5.3 | 6 | 3 | 28.5 ± 5.3 | 6 | 3 | 31.7 ± 2.4 | 6 | 3 | 0.147 | 20 | 6 | 6.02 |
| Sup 4B | Cre-WT | Phenylephrine | 10 nmol/L | 32.3 ± 3.2 | 6 | 3 | 38.2 ± 2.6 | 6 | 3 | 34.9 ± 2.9 | 6 | 3 | 21.1 ± 6.3 | 6 | 3 | 27.9 ± 5.5 | 6 | 3 | 32.5 ± 2.5 | 6 | 3 | 0.718 | 24 | 4 | 4.75 |
| Sup 4B | Cre-WT | Phenylephrine | 100 nmol/L | 40.2 ± 3.8 | 6 | 3 | 48.1 ± 4.0 | 6 | 3 | 43.8 ± 3.2 | 6 | 3 | 27.7 ± 8.0 | 6 | 3 | 38.1 ± 5.1 | 6 | 3 | 41.6 ± 2.8 | 6 | 3 | 0.403 | 20 | 4 | 3.98 |
| Sup 4B | Cre-WT | Phenylephrine | 1 µmol/L | 60.7 ± 3.8 | 6 | 3 | 65.2 ± 2.9 | 6 | 3 | 63.0 ± 1.8 | 6 | 3 | 61.9 ± 2.7 | 6 | 3 | 58.9 ± 5.5 | 6 | 3 | 61.7 ± 2.6 | 6 | 3 | 1.000 | 20 | 6 | 1.23 |
| Sup 4B | Cre-WT | Phenylephrine | 10 µmol/L | 72.9 ± 2.1 | 6 | 3 | 75.9 ± 2.3 | 6 | 3 | 71.7 ± 1.7 | 6 | 3 | 73.0 ± 2.6 | 6 | 3 | 74.0 ± 2.6 | 6 | 3 | 72.8 ± 1.8 | 6 | 3 | 1.000 | 20 | 0 | 1.45 |
| 1E | Clock$^{\Delta19/\Delta19}$ | Myogenic | 20 mmHg | 24.3 ± 4.4 | 7 | 4 | 14.9 ± 3.8 | 6 | 3 | 13.9 ± 2.2 | 6 | 3 | 17.4 ± 4.2 | 5 | 3 | 16.5 ± 5.5 | 5 | 3 | 21.5 ± 6.1 | 6 | 4 | 1.000 | 24 | 6 | 2.33 |
| 1E | Clock$^{\Delta19/\Delta19}$ | Myogenic | 40 mmHg | 29.3 ± 3.9 | 7 | 4 | 27.9 ± 3.4 | 6 | 3 | 28.4 ± 1.9 | 6 | 3 | 28.2 ± 4.4 | 5 | 3 | 26.1 ± 6.1 | 5 | 3 | 27.3 ± 4.5 | 6 | 4 | 1.000 | 24 | 6 | 2.97 |
| 1E | Clock$^{\Delta19/\Delta19}$ | Myogenic | 60 mmHg | 33.8 ± 3.8 | 7 | 4 | 39.8 ± 4.3 | 6 | 3 | 36.7 ± 3.6 | 6 | 3 | 35.7 ± 4.7 | 5 | 3 | 35.0 ± 4.3 | 5 | 3 | 36.3 ± 2.7 | 6 | 4 | 0.205 | 24 | 6 | 5.02 |
| 1E | Clock$^{\Delta19/\Delta19}$ | Myogenic | 80 mmHg | 35.7 ± 3.8 | 7 | 4 | 44.1 ± 2.6 | 6 | 3 | 40.7 ± 3.5 | 6 | 3 | 38.8 ± 4.0 | 5 | 3 | 37.1 ± 3.8 | 5 | 3 | 40.4 ± 3.1 | 6 | 4 | 0.067 | 24 | 6 | 5.20 |
| 1E | Clock$^{\Delta19/\Delta19}$ | Myogenic | 100 mmHg | 34.4 ± 4.3 | 7 | 4 | 45.4 ± 2.0 | 6 | 3 | 41.6 ± 2.7 | 6 | 3 | 39.1 ± 4.6 | 5 | 3 | 39.3 ± 6.0 | 5 | 3 | 42.4 ± 3.2 | 6 | 4 | 0.294 | 24 | 6 | 4.47 |
| Sup 3A | Clock$^{\Delta19/\Delta19}$ | Phenylephrine | 1 nmol/L | 34.0 ± 2.0 | 7 | 4 | 31.2 ± 3.3 | 6 | 3 | 27.9 ± 4.0 | 6 | 3 | 27.2 ± 3.7 | 5 | 3 | 27.9 ± 4.3 | 5 | 3 | 27.4 ± 5.0 | 6 | 4 | 0.446 | 24 | 4 | 5.16 |
| Sup 3A | Clock$^{\Delta19/\Delta19}$ | Phenylephrine | 10 nmol/L | 39.6 ± 1.8 | 7 | 4 | 39.7 ± 3.2 | 6 | 3 | 31.8 ± 3.5 | 6 | 3 | 33.6 ± 3.4 | 5 | 3 | 33.2 ± 3.4 | 5 | 3 | 31.6 ± 4.8 | 6 | 4 | 0.050 | 24 | 4 | 6.63 |
| Sup 3A | Clock$^{\Delta19/\Delta19}$ | Phenylephrine | 100 nmol/L | 48.8 ± 2.3 | 7 | 4 | 53.5 ± 2.3 | 6 | 3 | 47.7 ± 2.8 | 6 | 3 | 49.0 ± 3.7 | 5 | 3 | 51.8 ± 4.9 | 5 | 3 | 49.0 ± 6.4 | 6 | 4 | 0.109 | 24 | 6 | 4.81 |
| Sup 3A | Clock$^{\Delta19/\Delta19}$ | Phenylephrine | 1 µmol/L | 66.4 ± 2.3 | 7 | 4 | 68.0 ± 3.0 | 6 | 3 | 66.1 ± 2.1 | 6 | 3 | 69.9 ± 3.2 | 5 | 3 | 68.0 ± 3.8 | 5 | 3 | 74.5 ± 3.8 | 6 | 4 | 1.000 | 24 | 8 | 1.34 |
| Sup 3A | Clock$^{\Delta19/\Delta19}$ | Phenylephrine | 10 µmol/L | 70.9 ± 2.8 | 7 | 4 | 75.8 ± 2.4 | 6 | 3 | 69.8 ± 3.8 | 6 | 3 | 75.9 ± 1.4 | 5 | 3 | 74.4 ± 3.8 | 5 | 3 | 78.7 ± 2.9 | 6 | 4 | 1.000 | 24 | 18 | 2.14 |
| -- | Clock$^{\Delta19/\Delta19}$ | KCl | 60 mmol/L | 67.9 ± 5.3 | 6 | 3 | 57.0 ± 3.8 | 6 | 3 | 71.5 ± 4.8 | 6 | 3 | 57.0 ± 4.9 | 6 | 3 | 41.9 ± 4.9 | 4 | 3 | 62.0 ± 7.3 | 6 | 4 | 0.893 | 24 | 6 | 7.50 |
| 1G | Sm-Bmal1 KO | Myogenic | 20 mmHg | 11.3 ± 4.0 | 6 | 3 | 15.4 ± 4.5 | 6 | 3 | 16.5 ± 4.3 | 6 | 3 | 17.3 ± 5.1 | 6 | 3 | 13.3 ± 6.5 | 5 | 3 | 21.5 ± 5.3 | 6 | 3 | 1.000 | 20 | 8 | 3.31 |
| 1G | Sm-Bmal1 KO | Myogenic | 40 mmHg | 34.6 ± 4.1 | 6 | 3 | 32.0 ± 3.7 | 6 | 3 | 31.2 ± 5.5 | 6 | 3 | 37.8 ± 4.3 | 6 | 3 | 29.5 ± 5.7 | 5 | 3 | 39.5 ± 4.4 | 6 | 3 | 1.000 | 24 | 18 | 1.73 |
| 1G | Sm-Bmal1 KO | Myogenic | 60 mmHg | 45.4 ± 3.0 | 6 | 3 | 43.5 ± 3.3 | 6 | 3 | 42.5 ± 3.1 | 6 | 3 | 47.9 ± 3.4 | 6 | 3 | 43.0 ± 4.6 | 5 | 3 | 46.3 ± 3.8 | 6 | 3 | 1.000 | 20 | 16 | 1.14 |
| 1G | Sm-Bmal1 KO | Myogenic | 80 mmHg | 49.2 ± 3.0 | 6 | 3 | 48.3 ± 3.3 | 6 | 3 | 46.4 ± 2.1 | 6 | 3 | 52.1 ± 3.0 | 6 | 3 | 48.2 ± 4.7 | 5 | 3 | 49.8 ± 3.8 | 6 | 3 | 1.000 | 20 | 16 | 1.93 |
| 1G | Sm-Bmal1 KO | Myogenic | 100 mmHg | 51.3 ± 3.0 | 6 | 3 | 50.4 ± 3.3 | 6 | 3 | 48.5 ± 2.2 | 6 | 3 | 54.9 ± 3.0 | 6 | 3 | 50.3 ± 4.6 | 5 | 3 | 50.6 ± 3.6 | 6 | 3 | 1.000 | 20 | 16 | 1.87 |
| Sup 3B | Sm-Bmal1 KO | Phenylephrine | 1 nmol/L | 36.5 ± 5.4 | 6 | 3 | 36.9 ± 3.2 | 6 | 3 | 37.5 ± 3.1 | 6 | 3 | 41.5 ± 4.3 | 6 | 3 | 29.9 ± 5.7 | 5 | 3 | 38.8 ± 4.1 | 6 | 3 | 1.000 | 20 | 8 | 3.04 |
| Sup 3B | Sm-Bmal1 KO | Phenylephrine | 10 nmol/L | 39.3 ± 5.2 | 6 | 3 | 38.4 ± 3.8 | 6 | 3 | 40.2 ± 3.2 | 6 | 3 | 42.4 ± 4.3 | 6 | 3 | 33.3 ± 4.7 | 5 | 3 | 40.8 ± 3.9 | 6 | 3 | 1.000 | 20 | 10 | 2.15 |
| Sup 3B | Sm-Bmal1 KO | Phenylephrine | 100 nmol/L | 47.7 ± 4.2 | 6 | 3 | 51.8 ± 3.5 | 6 | 3 | 49.4 ± 2.9 | 6 | 3 | 52.3 ± 4.2 | 6 | 3 | 46.6 ± 4.9 | 5 | 3 | 55.1 ± 3.8 | 6 | 3 | 1.000 | 24 | 0 | 1.98 |
| Sup 3B | Sm-Bmal1 KO | Phenylephrine | 1 µmol/L | 67.9 ± 4.2 | 6 | 3 | 68.9 ± 3.3 | 6 | 3 | 68.2 ± 1.8 | 6 | 3 | 70.8 ± 2.5 | 6 | 3 | 63.0 ± 3.1 | 5 | 3 | 71.3 ± 2.7 | 6 | 3 | 1.000 | 20 | 8 | 2.17 |
| Sup 3B | Sm-Bmal1 KO | Phenylephrine | 10 µmol/L | 78.7 ± 2.3 | 6 | 3 | 77.5 ± 1.7 | 6 | 3 | 75.3 ± 2.7 | 6 | 3 | 76.0 ± 2.6 | 6 | 3 | 72.2 ± 2.2 | 5 | 3 | 80.9 ± 1.4 | 6 | 3 | 0.347 | 20 | 2 | 2.78 |
| 2A | TNF KO | Myogenic | 20 mmHg | 16.5 ± 1.9 | 6 | 3 | 8.1 ± 2.7 | 5 | 3 | 10.3 ± 3.2 | 6 | 3 | 9.7 ± 3.4 | 6 | 3 | 11.3 ± 3.0 | 5 | 3 | 6.7 ± 1.9 | 6 | 3 | 1.000 | 20 | 18 | 3.46 |
| 2A | TNF KO | Myogenic | 40 mmHg | 19.1 ± 3.0 | 6 | 3 | 11.4 ± 3.1 | 5 | 3 | 16.0 ± 3.6 | 6 | 3 | 18.8 ± 4.6 | 6 | 3 | 18.5 ± 4.3 | 5 | 3 | 13.1 ± 5.3 | 6 | 3 | 1.000 | 20 | 16 | 3.65 |
| 2A | TNF KO | Myogenic | 60 mmHg | 33.5 ± 2.7 | 6 | 3 | 21.6 ± 3.9 | 5 | 3 | 28.7 ± 1.9 | 6 | 3 | 31.7 ± 2.6 | 6 | 3 | 28.8 ± 2.7 | 5 | 3 | 21.8 ± 4.8 | 6 | 3 | 0.568 | 20 | 14 | 0.91 |
| 2A | TNF KO | Myogenic | 80 mmHg | 39.4 ± 2.7 | 6 | 3 | 26.1 ± 2.3 | 5 | 3 | 33.9 ± 1.9 | 6 | 3 | 37.1 ± 2.9 | 6 | 3 | 36.0 ± 1.0 | 5 | 3 | 31.5 ± 3.6 | 6 | 3 | 0.158 | 20 | 16 | 4.30 |
| 2A | TNF KO | Myogenic | 100 mmHg | 40.7 ± 2.8 | 6 | 3 | 30.2 ± 2.6 | 5 | 3 | 37.5 ± 2.0 | 6 | 3 | 40.6 ± 2.9 | 6 | 3 | 40.4 ± 0.9 | 5 | 3 | 34.9 ± 3.3 | 6 | 3 | 0.259 | 20 | 16 | 3.70 |
| 2C | TNF KO | Phenylephrine | 1 nmol/L | 31.8 ± 2.5 | 6 | 3 | 19.5 ± 3.0 | 5 | 3 | 27.1 ± 2.5 | 6 | 3 | 28.9 ± 3.6 | 6 | 3 | 25.0 ± 4.5 | 5 | 3 | 23.9 ± 2.5 | 6 | 3 | 1.000 | 20 | 16 | 3.57 |
| 2C | TNF KO | Phenylephrine | 10 nmol/L | 32.0 ± 2.7 | 6 | 3 | 19.8 ± 3.4 | 5 | 3 | 27.9 ± 2.6 | 6 | 3 | 31.3 ± 3.7 | 6 | 3 | 25.6 ± 4.5 | 5 | 3 | 23.0 ± 2.7 | 6 | 3 | 0.663 | 20 | 14 | 1.05 |
| 2C | TNF KO | Phenylephrine | 100 nmol/L | 39.0 ± 2.4 | 6 | 3 | 22.0 ± 4.2 | 5 | 3 | 30.6 ± 3.3 | 6 | 3 | 33.9 ± 3.5 | 6 | 3 | 29.0 ± 4.2 | 5 | 3 | 26.8 ± 2.7 | 6 | 3 | 1.000 | 20 | 16 | 5.02 |
| 2C | TNF KO | Phenylephrine | 1 µmol/L | 58.3 ± 2.4 | 6 | 3 | 37.8 ± 3.1 | 5 | 3 | 45.9 ± 4.6 | 6 | 3 | 49.8 ± 4.5 | 6 | 3 | 48.2 ± 1.6 | 5 | 3 | 46.0 ± 4.2 | 6 | 3 | 0.800 | 20 | 0 | 0.96 |
| 2C | TNF KO | Phenylephrine | 10 µmol/L | 76.9 ± 3.3 | 6 | 3 | 70.3 ± 4.0 | 5 | 3 | 69.5 ± 6.1 | 6 | 3 | 74.3 ± 4.6 | 6 | 3 | 72.4 ± 3.1 | 5 | 3 | 70.8 ± 5.0 | 6 | 3 | 1.000 | 20 | 16 | 1.91 |

Data are means ± standard error measurements, where n equals the number of measurements (i.e., vessels) and N equals the number of mice used to generate those samples. *JTK P* is the Bonferroni-adjusted minimal P value calculated by JTK_CYCLE. *Acrophase* is the Zeitgeber time of the sinusoidal rhythm peak calculated by JTK_CYCLE. * denotes a statistically significant circadian rhythm ($P<0.05$).

*Abbreviations:* Cre-WT = tamoxifen-treated, Cre-expressing wild-type controls; KCl = potassium chloride; Sm-Bmal1 KO = smooth muscle specific Bmal1 knockout. TNF KO = tumor necrosis factor knockout.

**Supplemental Table 8: JTK_CYCLE analysis of gene expression in cremaster arteries isolated from naïve wild-type and tumor necrosis factor knockout mice.**

| Figure | Genotype | Gene | ZT3 | n | ZT7 | n | ZT11 | n | ZT15 | n | ZT19 | n | ZT23 | n | JTK P | Period | Acrophase | Amplitude |
|---|---|---|---|---|---|---|---|---|---|---|---|---|---|---|---|---|---|---|
| Sup 5A | Wild-Type | Bmal1 | 11.02 ± 2.46 | 3 | 2.35 ± 0.36 | 3 | 1.00 ± 0.22 | 3 | 2.52 ± 0.35 | 3 | 7.08 ± 0.41 | 3 | 15.41 ± 2.42 | 3 | < 0.001 * | 24 | 22 | 5.30 |
| Sup 5B | Wild-Type | Per2 | 1.00 ± 0.04 | 3 | 2.47 ± 0.33 | 3 | 5.96 ± 1.36 | 3 | 5.27 ± 0.26 | 3 | 2.85 ± 0.21 | 3 | 2.25 ± 0.62 | 3 | < 0.001 * | 20 | 10 | 2.18 |
| Sup 5C | Wild-Type | Clock | 1.79 ± 0.17 | 3 | 1.09 ± 0.10 | 3 | 1.00 ± 0.14 | 3 | 1.13 ± 0.07 | 3 | 1.30 ± 0.05 | 3 | 1.95 ± 0.34 | 3 | 0.001 * | 24 | 22 | 0.37 |
| 2K | Wild-Type | TNF | 1.23 ± 0.15 | 5 | 1.06 ± 0.13 | 5 | 1.11 ± 0.21 | 4 | 1.06 ± 0.14 | 5 | 1.00 ± 0.12 | 5 | 1.59 ± 0.24 | 5 | 0.765 | 20 | 2 | 0.06 |
| 2K | Wild-Type | TNFR1 | 1.22 ± 0.16 | 6 | 1.18 ± 0.12 | 7 | 1.00 ± 0.11 | 7 | 1.14 ± 0.13 | 6 | 1.16 ± 0.10 | 6 | 1.18 ± 0.19 | 6 | 1.000 | 20 | 0 | 0.07 |
| 2K | Wild-Type | TNFR2 | 1.44 ± 0.05 | 6 | 1.25 ± 0.12 | 7 | 1.01 ± 0.10 | 7 | 1.24 ± 0.17 | 6 | 1.11 ± 0.07 | 6 | 1.05 ± 0.06 | 6 | 0.874 | 20 | 0 | 0.13 |
| 2L | Wild-Type | CK1 delta | 1.13 ± 0.22 | 2 | 1.00 ± 0.11 | 2 | 1.11 ± 0.14 | 2 | 1.04 ± 0.04 | 2 | 1.25 ± 0.05 | 2 | 1.01 ± 0.12 | 2 | 1.000 | 20 | 18 | 0.11 |
| 2L | Wild-Type | CK1 epsilon | 1.00 ± 0.15 | 2 | 1.07 ± 0.13 | 2 | 1.36 ± 0.02 | 2 | 1.16 ± 0.34 | 2 | 1.39 ± 0.02 | 2 | 1.05 ± 0.05 | 2 | 0.903 | 24 | 14 | 0.24 |
| Sup 5D | TNF KO | Bmal1 | 22.69 ± 3.98 | 3 | 2.22 ± 0.40 | 3 | 1.00 ± 0.26 | 3 | 3.28 ± 0.36 | 3 | 11.63 ± 1.04 | 3 | 20.98 ± 1.92 | 3 | < 0.001 * | 20 | 0 | 6.96 |
| Sup 5E | TNF KO | Per2 | 1.00 ± 0.08 | 3 | 4.56 ± 0.22 | 3 | 9.92 ± 0.90 | 3 | 9.95 ± 0.92 | 3 | 8.89 ± 0.95 | 3 | 4.57 ± 0.90 | 3 | < 0.001 * | 24 | 12 | 4.42 |
| Sup 5F | TNF KO | Clock | 1.50 ± 0.06 | 3 | 1.25 ± 0.18 | 3 | 1.00 ± 0.13 | 3 | 1.48 ± 0.12 | 3 | 2.14 ± 0.22 | 3 | 1.79 ± 0.25 | 3 | 0.006 * | 20 | 18 | 0.34 |

Data are means ± standard error measurements, where n equals the number of samples (each sample generated from 1 mouse). *JTK P* is the Bonferroni-adjusted minimal P value calculated by JTK_CYCLE. *Acrophase* is the Zeitgeber time of the sinusoidal rhythm peak calculated by JTK_CYCLE. * denotes a statistically significant circadian rhythm ($P<0.05$).

*Abbreviations:* CK1 = casein kinase 1; TNF = tumor necrosis factor; TNFR = tumor necrosis factor receptor; TNF KO = tumor necrosis factor knockout.

**Supplemental Table 9: JTK_CYCLE analysis of clock gene expression in arteries isolated from mice with myocardial infarction or sham surgical procedure.**

| Figure | Condition | Gene | ZT3 | n | ZT7 | n | ZT11 | n | ZT15 | n | ZT19 | n | ZT23 | n | JTK P | Period | Acrophase | Amplitude |
|---|---|---|---|---|---|---|---|---|---|---|---|---|---|---|---|---|---|---|
| 3E | Sham | Bmal1 | 8.67 ± 0.60 | 4 | 3.12 ± 0.43 | 4 | 1.00 ± 0.13 | 4 | 1.94 ± 0.19 | 4 | 7.13 ± 0.81 | 4 | 14.13 ± 1.24 | 4 | **< 0.001 *** | 24 | 22 | 5.23 |
| 3E | Sham | Per2 | 1.00 ± 0.10 | 4 | 3.25 ± 0.55 | 4 | 6.75 ± 1.13 | 4 | 5.63 ± 0.98 | 4 | 3.25 ± 0.39 | 4 | 1.88 ± 0.38 | 4 | **< 0.001 *** | 20 | 10 | 2.33 |
| 3E | Sham | Rev-Erb | 7.19 ± 1.23 | 4 | 9.97 ± 0.72 | 4 | 5.84 ± 0.57 | 4 | 1.98 ± 0.32 | 4 | 1.00 ± 0.12 | 4 | 3.16 ± 0.37 | 4 | **< 0.001 *** | 24 | 4 | 3.78 |
| 3E | Sham | Clock | 1.22 ± 1.23 | 4 | 1.09 ± 0.13 | 4 | 0.99 ± 0.07 | 4 | 1.00 ± 0.08 | 4 | 1.17 ± 0.12 | 4 | 1.52 ± 0.14 | 4 | **0.025 *** | 24 | 22 | 0.18 |
| 3E | MI | Bmal1 | 9.63 ± 0.82 | 3 | 5.51 ± 0.82 | 3 | 1.02 ± 0.23 | 3 | 2.49 ± 0.48 | 3 | 7.77 ± 1.35 | 3 | 13.75 ± 1.43 | 3 | **< 0.001 *** | 24 | 22 | 5.18 |
| 3E | MI | Per2 | 1.24 ± 0.35 | 3 | 3.26 ± 0.73 | 3 | 6.13 ± 0.61 | 3 | 5.96 ± 0.61 | 3 | 3.76 ± 0.67 | 3 | 1.53 ± 0.01 | 3 | **< 0.001 *** | 24 | 12 | 2.52 |
| 3E | MI | Rev-Erb | 7.20 ± 0.15 | 3 | 9.11 ± 0.55 | 3 | 6.08 ± 1.13 | 3 | 2.76 ± 0.43 | 3 | 0.95 ± 0.08 | 3 | 3.76 ± 1.27 | 3 | **< 0.001 *** | 24 | 4 | 3.71 |
| 3E | MI | Clock | 1.43 ± 0.15 | 3 | 1.18 ± 0.07 | 3 | 0.82 ± 0.04 | 3 | 1.05 ± 0.10 | 3 | 1.25 ± 0.10 | 3 | 1.40 ± 0.08 | 3 | **< 0.001 *** | 20 | 0 | 0.25 |

Data are means ± standard error measurements, where n equals the number of samples (each sample generated from 1 mouse). *JTK P* is the Bonferroni-adjusted minimal P value calculated by JTK_CYCLE. *Acrophase* is the Zeitgeber time of the sinusoidal rhythm peak calculated by JTK_CYCLE. * denotes a statistically significant circadian rhythm ($P<0.05$).

*Abbreviation:* MI = myocardial infarction.

**Supplemental Table 10: Hemodynamic and cardiac parameters in $Clock^{\Delta19/\Delta19}$ and control mice following myocardial infarction or sham surgical procedure.**

| Parameter | $Clock^{\Delta19/\Delta19}$ MI | Wild Type MI | $Clock^{\Delta19/\Delta19}$ Sham | Wild Type Sham |
|---|---|---|---|---|
| n | 8 | 8 | 8 | 8 |
| **Echocardiography (1 week)** | | | | |
| LVIDd (mm) | 4.65±0.07 | 4.69±0.07 | 4.06±0.02 | 4.00±0.04 |
| LVIDs (mm) | 3.35±0.09 | 3.42±0.09 | 2.44±0.02 | 2.41±0.04 |
| EF (%) | 60.83±1.56 | 59.71±1.56 | 76.19±0.84 | 76.73±0.74 |
| FS (%) | 28.02±0.98 | 27.42±0.98 | 39.69±0.51 | 39.77±0.65 |
| HR (bpm) | 438±4 | 440±7 | 455±7 | 458±7 |
| **Echocardiography (8 week)** | | | | |
| LVIDd (mm) | **4.90±0.08*** | 5.31±0.10 | 4.05±0.02 | 4.05±0.02 |
| LVIDs (mm) | **3.62±0.10*** | 4.17±0.11 | 2.44±0.01 | 2.45±0.05 |
| EF (%) | **57.69±1.67*** | 49.93±1.16 | 76.51±0.43 | 76.23±0.94 |
| FS (%) | **26.18±1.00*** | 21.70±0.63 | 39.56±0.37 | 39.40±0.82 |
| HR (bpm) | 448±6 | 461±4 | 446±4 | 462±9 |
| **Morphometry (8 week)** | | | | |
| HW (mg) | 158.50±1.61 | 157.38±2.68 | 141.00±2.47 | 131.13±0.93 |
| HW:BW (mg/g) | 4.64±0.16 | 5.09±0.19 | 4.16±0.18 | 4.12±0.08 |
| HW:TL (mg/mm) | 7.76±0.08 | 7.79±0.14 | 6.96±0.13 | 6.50±0.09 |
| **Pressure-volume Hemodynamics (8 week)** | | | | |
| LVESP (mmHg) | 88.17±1.05 | 90.59±1.22 | 99.61±0.89 | 98.77±0.50 |
| LVEDP (mmHg) | 0.30±0.69 | 0.72±0.39 | -0.35±0.17 | 0.27±0.43 |
| LVESV (µl) | **27.48±1.97*** | 39.00±2.37 | 9.08±1.16 | 8.92±0.74 |
| LVEDV (µl) | **49.95±2.03*** | 56.75±2.07 | 34.68±1.46 | 34.28±1.44 |
| SV (µl) | **22.47±0.66*** | 17.74±0.44 | 25.09±1.05 | 24.58±0.90 |
| CO (mL/min) | **12.24±0.39*** | 10.08±0.36 | 13.52±0.43 | 13.73±0.54 |
| $+dP/dt_{max}$ | 7954±392 | 7862±472 | 10173±542 | 9617±512 |
| $-dP/dt_{min}$ | 6771±689 | 6442±392 | 9970±668 | 9354±629 |
| SBP (mmHg) | **85.98±0.81*** | 90.73±0.91 | 96.84±0.84 | 97.01±0.38 |
| DBP (mmHg) | 57.88±1.08 | 58.76±0.80 | 62.69±1.08 | 66.44±0.73 |
| MAP (mmHg) | **66.58±0.74*** | 68.72±0.48 | 73.33±0.74 | 74.55±0.44 |
| TPR (mmHg/mL/min) | **5.48±0.19*** | 6.88±0.24 | 5.46±0.17 | 5.49±0.22 |
| HR (bpm) | 546±16 | 567±20 | 543±20 | 559±14 |

Data are means ± standard error measurements, where n equals the number of mice. * denotes $P<0.05$ for $Clock^{\Delta19/\Delta19}$ MI versus wild-type MI.

*Abbreviations:* CO = cardiac output; DBP = diastolic blood pressure; $dP/dt_{max}$ = maximum first derivative of left ventricular pressure; and $dP/dt_{min}$ = minimum first derivative of left ventricular pressure; EF = ejection fraction; FS = fractional shortening; HR = heart rate; HW = heart weight; HW:BW = HW:body weight ratio; HW:TL = HW:tibia length ratio; LV = left ventricle; LVEDP = LV end diastolic pressure; LVESP = LV end systolic pressure; LVEDV = LV end diastolic volume; LVESV = LV end systolic volume; LVIDd = LV internal dimensions at diastole; LVIDs = LV internal dimensions at systole; MAP = mean arterial pressure; MI = myocardial infarction; SBP = systolic blood pressure; SV = stroke volume; TPR = total peripheral resistance.

**Supplemental Table 11: Hemodynamic and cardiac parameters in Sm-Bmal1 KO and control mice following myocardial infarction or sham surgical procedure.**

| Parameter | Sm-Bmal1-KO MI | Cre-WT MI | Sm-Bmal1-KO Sham | Cre-WT Sham |
|---|---|---|---|---|
| **Echocardiography (4 week)** | | | | |
| n | 10 | 6 | 9 | 7 |
| LVIDd (mm) | 4.18±0.13 | 4.66±0.19 | 3.85±0.07 | 3.75±0.09 |
| LVIDs (mm) | 3.14±0.17 | 3.81±0.28 | 2.62±0.05 | 2.45±0.09 |
| EF (%) | 57.05±3.82 | 45.10±5.39 | 68.27±1.06 | 71.64±2.26 |
| FS (%) | 25.18±2.31 | 18.58±2.77 | 31.85±0.73 | 34.61±1.85 |
| HR (bpm) | 445±17 | 462±33 | 443±21 | 474±20 |
| **Echocardiography (8 week)** | | | | |
| n | 12 | 5 | 9 | 8 |
| LVIDd (mm) | **4.33±0.11*** | 4.99±0.18 | 3.96±0.08 | 3.88±0.08 |
| LVIDs (mm) | **3.31±0.16*** | 4.13±0.19 | 2.58±0.07 | 2.61±0.10 |
| EF (%) | **55.14±3.24*** | 42.96±4.09 | 72.29±1.20 | 69.38±2.39 |
| FS (%) | **23.94±1.85*** | 17.30±1.97 | 34.92±0.95 | 32.91±1.74 |
| HR (bpm) | 463±19 | 458±38 | 478±24 | 451±32 |
| **Morphometry (8 week)** | | | | |
| n | 12 | 5 | 7 | 8 |
| HW (mg) | 133.00±3.43 | 139.50±6.24 | 112.71±3.39 | 124.00±2.83 |
| HW:BW (mg/g) | 5.04±0.12 | 4.96±0.21 | 4.67±0.13 | 4.75±0.13 |
| HW:TL (mg/mm) | 6.22±0.19 | 6.57±0.20 | 5.39±0.18 | 6.01±0.16 |
| **Pressure-volume Hemodynamics (8 week)** | | | | |
| n | 9 | 6 | 5 | 5 |
| LVESP (mmHg) | **93.58±0.96*** | 88.87±1.44 | 98.56±2.13 | 100.55±2.67 |
| LVEDP (mmHg) | 4.68±0.74 | 6.26±1.51 | 2.47±0.49 | 2.29±0.31 |
| LVESV (µl) | **20.11±1.23*** | 30.65±2.55 | 15.11±1.23 | 13.71±1.10 |
| LVEDV (µl) | **44.21±1.05*** | 51.61±2.12 | 43.00±1.73 | 40.42±1.46 |
| SV (µl) | **24.10±0.53*** | 20.96±0.83 | 27.89±1.71 | 26.71±1.26 |
| CO (mL/min) | **11.93±0.37*** | 9.67±0.36 | 13.51±0.43 | 13.09±0.48 |
| $+dP/dt_{max}$ | 7120±209 | 5976±580 | 8196±194 | 9260±114 |
| $-dP/dt_{min}$ | 6220±339 | 5506±589 | 8152±769 | 9038±381 |
| SBP (mmHg) | **94.31±1.02*** | 88.34±1.55 | 99.69±2.39 | 98.01±1.52 |
| DBP (mmHg) | 65.67±1.09 | 63.20±0.92 | 67.98±2.22 | 66.91±2.02 |
| MAP (mmHg) | **74.47±1.01*** | 70.86±0.68 | 77.76±2.20 | 76.50±1.72 |
| TPR (mmHg/mL/min) | **6.28±0.18*** | 7.38±0.25 | 5.78±0.28 | 5.88±0.29 |
| HR (bpm) | 496±16 | 464±22 | 488±18 | 492±14 |

Data are means ± standard error measurements, where n equals the number of mice. * denotes $P<0.05$ for Sm-Bmal1 -KO MI versus control MI.

*Abbreviations:* CO = cardiac output; DBP = diastolic blood pressure; $dP/dt_{max}$ = maximum first derivative of left ventricular pressure; and $dP/dt_{min}$ = minimum first derivative of left ventricular pressure; EF = ejection fraction; FS = fractional shortening; HR = heart rate; HW = heart weight; HW:BW = HW:body weight ratio; HW:TL = HW:tibia length ratio; LV = left ventricle; LVEDP = LV end diastolic pressure; LVESP = LV end systolic pressure; LVEDV = LV end diastolic volume; LVESV = LV end systolic volume; LVIDd = LV internal dimensions at diastole; LVIDs = LV internal dimensions at systole; MAP = mean arterial pressure; MI = myocardial infarction; SBP = systolic blood pressure; SV = stroke volume; TPR = total peripheral resistance.
